# A novel framework for inferring parameters of transmission from viral sequence data

**DOI:** 10.1371/journal.pgen.1007718

**Published:** 2018-10-16

**Authors:** Casper K. Lumby, Nuno R. Nene, Christopher J. R. Illingworth

**Affiliations:** 1 Department of Genetics, University of Cambridge, Cambridge, United Kingdom; 2 Department of Applied Mathematics and Theoretical Physics, University of Cambridge, Cambridge, United Kingdom; Imperial College London, UNITED KINGDOM

## Abstract

Transmission between hosts is a critical part of the viral lifecycle. Recent studies of viral transmission have used genome sequence data to evaluate the number of particles transmitted between hosts, and the role of selection as it operates during the transmission process. However, the interpretation of sequence data describing transmission events is a challenging task. We here present a novel and comprehensive framework for using short-read sequence data to understand viral transmission events, designed for influenza virus, but adaptable to other viral species. Our approach solves multiple shortcomings of previous methods for this purpose; for example, we consider transmission as an event involving whole viruses, rather than sets of independent alleles. We demonstrate how selection during transmission and noisy sequence data may each affect naive inferences of the population bottleneck, accounting for these in our framework so as to achieve a correct inference. We identify circumstances in which selection for increased viral transmission may or may not be identified from data. Applying our method to experimental data in which transmission occurs in the presence of strong selection, we show that our framework grants a more quantitative insight into transmission events than previous approaches, inferring the bottleneck in a manner that accounts for selection, both for within-host virulence, and for inherent viral transmissibility. Our work provides new opportunities for studying transmission processes in influenza, and by extension, in other infectious diseases.

## Author summary

In order to spread, pathogens must not only be able to grow within an infected host, but also transmit to found new infections. Population genetics can exploit genome sequence data to provide a great deal of insight into transmission processes. For example, the number of particles which found a new infection determine the extent to which genetic diversity is passed from host to host. The identification of genetic variants which increase the propensity of a pathogen to transmit from host to host is a valuable step in understanding how an infection might spread. Here we set out a new population genetic framework for understanding transmission events from genome sequence data collected before and after transmission. Our approach corrects for the shortcomings of existing methods for this purpose, setting out a new baseline for the statistical analysis of transmission events. We demonstrate the ability of our method to draw novel quantitative insights by application to data from simulated and real transmission events.

## Introduction

Understanding viral transmission is a key task for viral epidemiology. The extent to which a virus is able to transmit between hosts determines whether it is likely to cause sporadic, local outbreaks, or spread to cause a global pandemic [1, 2]. In a transmission event, the transmission bottleneck, which specifies the number of viral particles founding a new infection, influences the amount of genetic diversity that is retained upon transmission, with important consequences for the evolutionary dynamics of the virus [3, 4].

Recent studies have used genome sequencing approaches to study transmission bottlenecks in influenza populations. In small animal studies, the use of neutral genetic markers has shown that the transmission bottleneck is dependent upon the route of transmission, whether by contact or aerosol transmission [5, 6]. In natural human influenza populations, where modification of the virus is not possible, population genetic methods have been used to analyse bottleneck sizes. Analyses of transmission have employed different approaches, exploiting the observation or non-observation of variant alleles [7] or using changes in allele frequencies to characterise the bottleneck under a model of genetic drift [8–11]. A recent publication improved this latter model, incorporating the uncertainty imposed upon allele frequencies by the process of within-host growth [12]. Two studies of within-household influenza transmission have provided strikingly different outcomes in the number of viruses involved in transmission, with estimates of 1-2 [13] and 100-200 [14] respectively, albeit that the veracity of the data used to generate the latter result has recently been challenged [15].

Another focus of research has been the role of selection during a transmission event; this is important in the context of the potential for new influenza strains to become transmissible between mammalian hosts [16, 17]. Studies examining transmissibility have assessed the potential for different strains of influenza to achieve droplet transmission between ferrets under laboratory conditions [18–21]; ferrets provide a useful, if imperfect, model for transmission between humans [22, 23]. The application of bioinformatic techniques to data from these experiments has identified ‘selective bottlenecks’ in the experimental evolution of these viruses [24, 25], whereby some genetic variants appear to be more transmissible than others. In these studies, selection has been considered in terms of the population diversity statistic *π*; changes in *π*_*N*_/*π*_*S*_, the ratio between non-synonymous and synonymous diversity, have been used to evaluate patterns of selection across different viral segments.

We here note the need for a greater clarity of thinking in the analysis of viral transmission events. For example, analysis of genetic variants in viral populations shows that synonymous and non-synonymous mutations both have fitness consequences for viruses [26, 27]; the use of synonymous variants as a neutral reference set may not hold. More fundamentally, in an event where the effective population size is small, the influences of selection and genetic drift may be of similar magnitude [28]. However selection is assessed, this implies a need to separate stochastic changes in a population from selection, especially where a transmission bottleneck may include only a small number of viruses [5, 13, 29]. It is possible for the attribution of a change in diversity to the action of selection, or the attribution of allele frequency change to genetic drift to be flawed. Given the increasing availability of sequence data, more sophisticated tools for the analysis of viral transmission are required.

**Fig 1:**
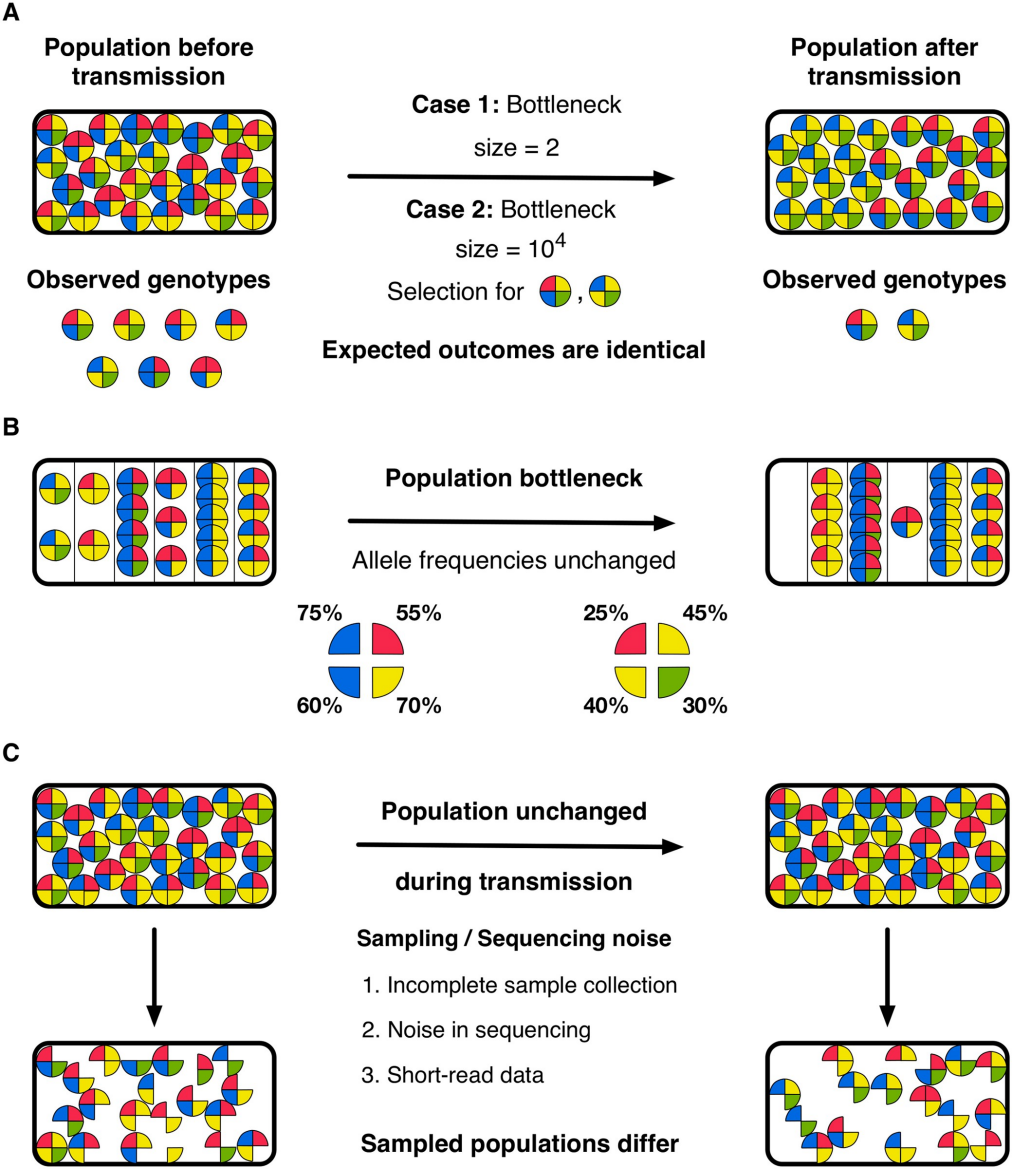
Challenges arising in the inference of transmission bottlenecks from viral sequence data. Circles represent idealised viral particles characterised by four distinct alleles. **A.** Reductions in population diversity cannot necessarily be attributed unambiguously to either a population bottleneck, or the action of selection. In the illustrated case, either a tight bottleneck without selection or a large bottleneck with strong selection could explain the change in the population during transmission. **B.** Straightforward statistics describing a population may generate misleading inferences of population bottleneck size. In the illustrated case, the genetic structure of a population is changed by a population bottleneck during transmission, but the frequency of each allele within the population does not change; an inference of bottleneck size derived from single-locus statistics would incorrectly be very large. **C.** Noise arising from the process of collecting and sequencing data is likely to produce differences between the observed populations, even in the event that the composition of the viral population was entirely unchanged during transmission.

Here we note three challenges in the analysis of data from viral transmission events. Firstly, selection can produce changes in a population equivalent to those arising through a neutral population bottleneck [30] (Fig 1A), making it necessary to distinguish between the two scenarios. A broad literature has considered the simultaneous inference of the magnitude of selection acting upon a variant along with an effective population size [31–36]. However, such approaches rely on the observation of an allele frequency at more than two time points so as to distinguish a deterministic model of selection (with an implied infinite effective population size) from a combined model of selection with genetic drift; such approaches cannot be directly applied to the analysis of viral transmission.

Secondly, inferences of transmission events need to account for the haplotype structure of viral populations, whereby whole viruses, rather than sets of independent alleles, are transmitted (Fig 1B). The low rate of homologous recombination in segments of the influenza virus [37, 38] implies that viral evolution proceeds at the haplotype level [39]; competition occurs between collections of linked alleles, or segments, rather than the individual alleles themselves. Under such circumstances, fitter variants do not always increase in frequency within a population [40–42]. Calculations of genetic drift, which are often derived from the evolution of independent variants [43], need to be adjusted to account for this more complex dynamics.

Thirdly, noise in the measurement of a population may influence the inferred size of a transmission bottleneck (Fig 1C). A broad range of studies have examined the effect of noise in variant calling and genome sequence analysis [44–51]; more recently formulae have been proposed to measure the precision with which allele frequencies can be defined given samples from a population [52–54]. Where small changes in allele frequencies are used to assess a population bottleneck, it is important to separate the effects of noise in the measurement of populations from genuine changes in a population.

We here describe a novel method for the inference of population bottlenecks in influenza which addresses the above issues. Our approach correctly evaluates changes in a population even where the data describing that change are affected by noise. It explicitly accounts for the haplotype structure of a population, utilising the data present within short sequence reads. Further, where these factors can be discriminated, our method distinguishes between the influences on the population of selection and the transmission bottleneck. Studies of viral evolution have highlighted the potential for payoffs between within-host viral growth and transmissibility [55]; given sufficient data we can evaluate how selection operates upon each of these phenotypes. Our model extends previous population genetic work on bottleneck inference to provide a more generalised model for the analysis of data spanning viral transmission events. Whilst the work presented here is modelled upon influenza viruses, the methods may be readily adapted to other viral species given minor modifications.

## Results

In the recent literature, the term ‘bottleneck’ has been applied to describe a reduction in the genetic diversity of a population (e.g. [56]), whether arising from selection or a numerical reduction in the size of a population. Here, we define a ‘bottleneck’ more strictly as a neutral process whereby a finite number of viral particles from one population found a subsequent generation of the population, either within the same host, or across a transmission event from host to recipient. Selection then constitutes a modification to this process whereby some viruses, because of their genotype, have a higher or lower probability of making it through the bottleneck to found the next generation.

**Fig 2:**
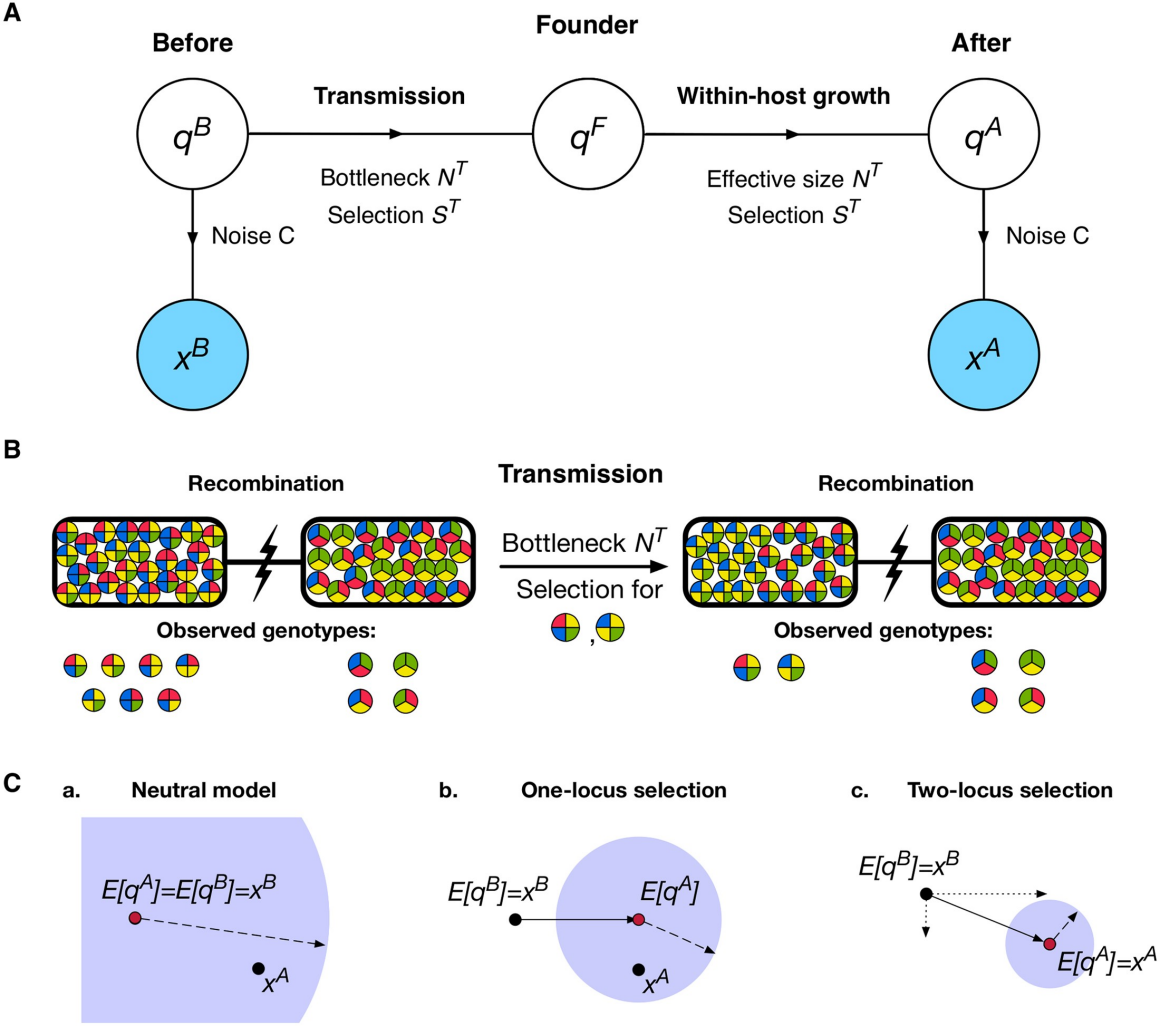
**A.** Basic model of transmission. A set of haplotypes exists at frequencies ***q***^*B*^ from which a noisy observation ***x***^*B*^ is made. During a transmission event, a total of *N*^*T*^ viruses are transferred under the influence of selection *S*^*T*^, establishing an infection in the next host described by ***q***^*F*^. Growth of the viral population within the host then occurs to produce the population ***q***^*A*^, influenced by genetic drift (characterised by the effective population size *N*^*G*^) and selection *S*^*G*^. Sampling of the final population gives the second observation ***x***^*A*^. **B.** Regions of the genome which are separated by recombination or reassortment are used to distinguish the effects of selection and a population bottleneck. Prior to transmission, the first region contains seven different genotypes spanning four variant loci whilst the second region harbours four genotypes covering three loci. As recombination between these two regions leaves them unlinked, selection acting on genotypes in one region has no impact on the fate of genotypes in the other region. Thus, where genetic diversity is reduced in the first region, the preservation of diversity in the second region attributes this change to the action of selection on the first, rather than a shared, and narrow, population bottleneck. **C.** Models of neutrality and selection are compared, as illustrated in this simplified diagram. Black dots represent observations ***x***^*B*^ and ***x***^*A*^ while the red dot indicates the inferred expected position of ***q***^*A*^. The solid line joining these (b,c) indicates the inferred action of selection, with dotted lines showing components of this vector (c). The blue circle represents the optimised variance in the position of ***q***^*A*^; the length of its radius, shown as a dashed line, is inversely related to the bottleneck size. In the neutral case, the difference between observations is explained by the bottleneck alone. More complex models of selection fit ***q***^*A*^ more closely to ***x***^*A*^ and with reduced variance, giving higher inferred values of *N*^*T*^.

We applied a population genetic method to make a joint inference of the bottleneck size and the extent of selection acting during a transmission event. We consider a scenario in which a viral population is transmitted from one host to another, with samples being collected before and after the transmission event (Fig 2A). In our model viruses are categorised as haplotypes, according to the alleles they harbour at polymorphic sites in the genome. Such haplotypes are not directly observable from short-read sequencing data. However, after identifying polymorphisms in the data it is possible to use short-read data to identify a set of haplotypes which collectively explain the observed sequence data across the course of a transmission event [52, 57]. The viral population is then represented as a vector of frequencies of haplotypes in this set; the population before transmission is represented by the vector ***q***^*B*^ (*B* denoting ‘Before transmission’). During transmission, a random sample of *N*^*T*^ viruses are passed on to the second host to give the founder population ***q***^*F*^. Selection for transmissibility, whereby genetic variants cause some viruses to be more transmissible than others, is described by the function *S*^*T*^. The potentially small size of the founder population means that the population evolves within the host under the influence of genetic drift to create the large post-transmission population ***q***^*A*^ (*A* denoting ‘After transmission’); this process is approximated in our model by a Wright-Fisher sampling process (representing genetic drift) with effective population size *N*^*G*^. Selection acting for within-host growth may further alter the genetic composition of the population; this effect is described by the function *S*^*G*^. Our method thus allows for a discrimination to be made between selection for increased within-host replication and selection for increased viral transmissibility. Observations of the population are collected before and after transmission via a noisy sequencing process to give the datasets ***x***^*B*^ and ***x***^*A*^. The extent of noise in the sampling and sequencing is characterised by the parameter *C* [52, 57]. Noise in our study was considered in terms of the precision with which the frequency of a variant can be specified by viral sequence data. Variant frequencies are measured in terms of the number of reads which report a given allele; in the absence of noise the uncertainty in the frequency would be that arising from a binomial distribution. Our noise parameter *C* describes the extent to which this uncertainty is increased. Smaller values of *C* increase the variance, reaching that of a non-informative uniform distribution at *C* = 0 whilst larger values represent lesser additional uncertainty, tending towards the binomial limit as *C* → ∞ (S1 Fig). Elsewhere we have noted that the parameter *C* and the absolute read depth of a sample can be converted into an ‘effective depth’ of sequencing [54]. In the limit of very deep sequencing the variance of an allele frequency tends towards that of a binomial distribution with sampling depth *C* + 1.

To summarise our approach, we note that both the transmission and within-host growth events can be represented as sampling processes, which may each be biased by the effect of selection. As such, given an estimate of the noise inherent to the sequencing process, and externally-derived estimates for *N*^*G*^ and *S*^*G*^, we can calculate an approximate likelihood for the parameters *N*^*T*^ and *S*^*T*^ given the observations ***x***^*B*^ and ***x***^*A*^. Maximising this likelihood gives an estimate for the size of the transmission bottleneck and the extent to which specific genetic variants within the pre-transmission population confer increased transmissibility upon viruses.

In our model we discriminate between changes in a population arising from selection and those arising due to the population bottleneck. This is achieved by considering regions of the genome between which recombination or reassortment has removed linkage disequilibrium between alleles (Fig 2B; compare with Fig 1A). As transmission involves whole viruses, the bottleneck *N*^*T*^ is preserved between regions. Meanwhile, in the absence of epistasis, selection acting upon one region of the virus does not influence the composition of the population in other parts of the genome. As such, a calculation encompassing multiple parts of the genome can estimate both *N*^*T*^ and the influence of selection; in the figure the case of a loose population bottleneck, with selection acting upon the first region is preferred. A model selection process [58] is used to distinguish models of neutral transmission from evolution under selection (Fig 2C). A full exposition of the model is given in the Methods section.

Here we used simulated data to evaluate the performance of our model under different circumstances. Having established the effect of sequencing noise on the inference of population bottlenecks, we demonstrate the ability of our method to correctly infer population bottlenecks from sequence data in the presence or absence of selection, and its ability to correctly identify variants conferring a benefit for viral transmissibility. We then applied our model to evaluate selection and population bottlenecks in a recently published transmission experiment [25], involving two sets of viral transmissions. Here Moncla et al. identified a ‘loose’ bottleneck in the first set of transmissions which became more stringent and selective in the second set. We here infer bottlenecks of around 2-6 viruses for each set of transmission events and identify a number of sites under selection for within-host adaptation. However, no evidence was found for the presence of selection for enhanced transmissibility. As we go on to show, where few viruses are transmitted, inferring selection for increased transmissibility is an inherently difficult task.

### Application to simulated data

#### Sequencing noise limits the maximum inferrable bottleneck

**Fig 3:**
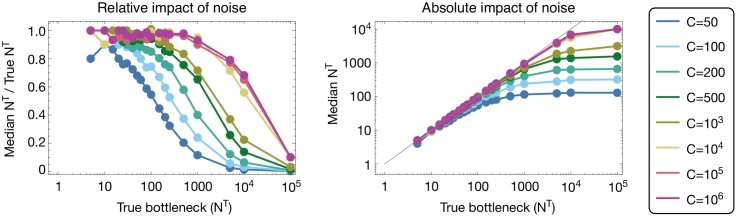
Influence of sequencing noise upon the ability to infer a population bottleneck size from genome sequence data. Median inferred bottlenecks are shown, calculated on the basis of 200 replicate simulations for each point. In the left-hand plot, a value of 1 indicates a correct bottleneck inference; in the right-hand plot, the absolute inferred bottleneck size is shown. Simulations were conducted under the assumption of selective neutrality, with no attempt to infer selection from the data.

Application of our model to simulated data describing neutral population bottlenecks showed that a lack of sequencing noise is critical for the correct inference of large population bottlenecks (Fig 3). Inferences of bottleneck sizes showed a limit on the inferred bottleneck size governed by noise in sequencing; where there was little noise in the data (i.e. at values of C greatly in excess of the bottleneck), a correct inference of the true population bottleneck was generally made. However, as noise increases, the inferred bottleneck reaches a plateau above which increases in the true bottleneck no longer affect the inferred bottleneck size. This result can be understood in terms of the extent to which the population bottleneck and noise contribute to the change in the viral population; where large numbers of viruses are transmitted, most of this signal is likely to result from noise. Here we note failures in the inferred bottleneck size even with very high *C*; these occur due to the finite read depth in our simulations, which was of order 10^4^. In these calculations a neutral method, in which selection was assumed to have no effect on the population, was used to make inferences from neutral simulations. A consistent value of *C* was used for simulation and inference purposes.

In a real dataset the extent of noise may be unknown. Further investigation showed bottleneck estimation to be relatively robust to an incorrect estimate of the extent of noise in a dataset, except where the extent of noise was substantially overestimated (S2 Fig). In general, an underestimate of the extent of noise in a dataset led to an inferred bottleneck size that was marginally lower than the value obtained given the true amount of noise; for example where the value *C* = 10^6^ was used to infer a bottleneck from data with *C* = 50, a bottleneck of true size *N*^*T*^ = 50 was inferred as *N*^*T*^ = 33. An overestimate of the extent of noise led to an overestimate of the size of the bottleneck with severe overestimation resulting in dramatically incorrect inferences. Therefore, while noise limits the potential of a method to identify large bottleneck sizes, underestimating the extent of noise in the data is generally the safer approach.

#### Variance in inferred transmission bottlenecks

**Fig 4:**
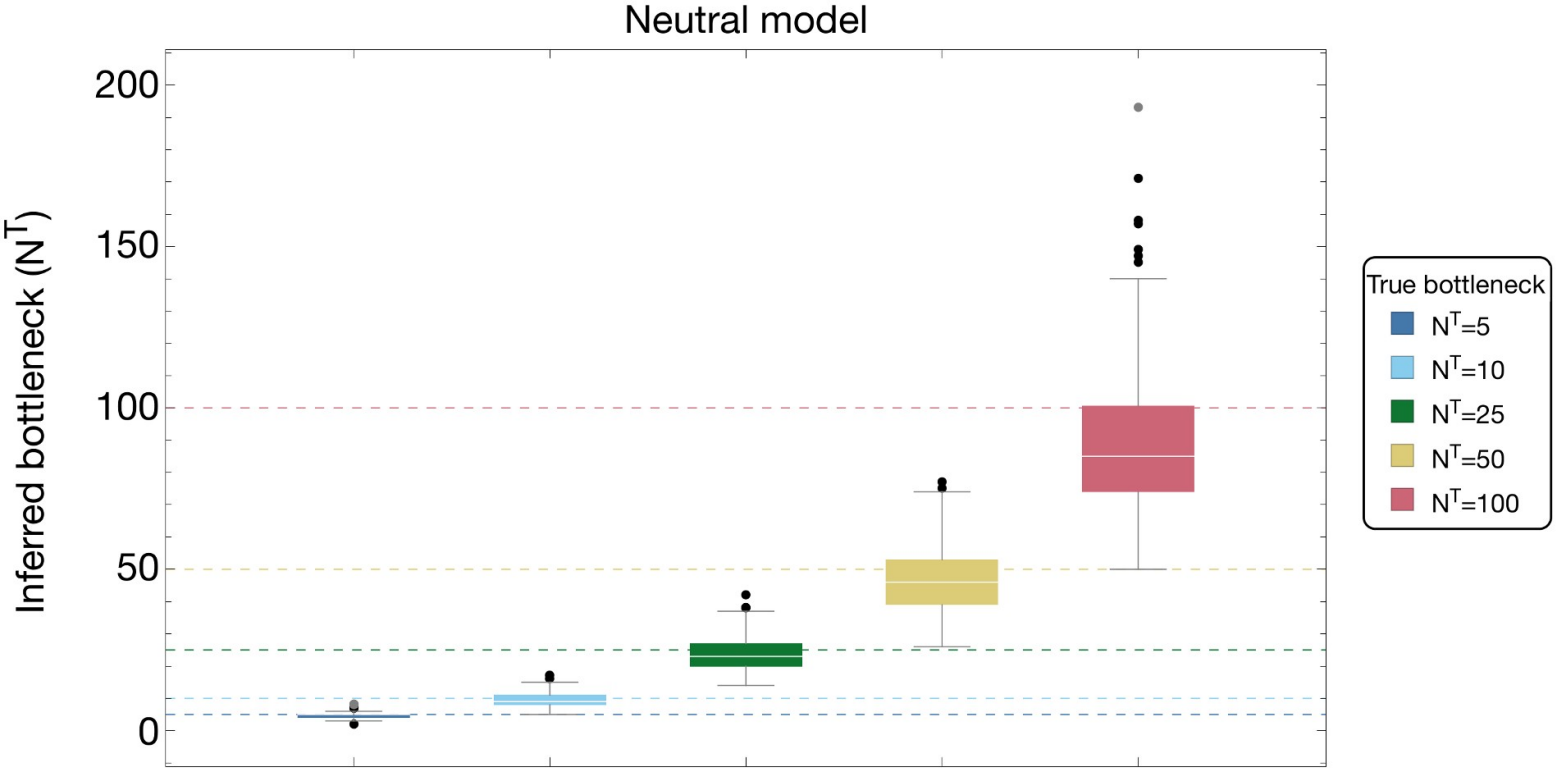
Inferred bottleneck sizes (*N*^*T*^) for true bottlenecks *N*^*T*^ = {5, 10, 25, 50, 100}. Results were generated by applying a neutral inference model to neutral simulated data. Inferences are shown for 200 simulations at each bottleneck size.

Results from individual simulations showed that our method could discriminate between bottleneck sizes that differ by a factor of three or above (Fig 4 and S4 Fig). Obtaining precision in an estimated bottleneck or effective population size is inherently a difficult task, relying on the estimate of the extent of a stochastic effect from limited data [33]. Across 200 simulations, the interquartile range in an inferred bottleneck spanned close to 28% of the true bottleneck size, with inferred values spanning a range of approximately 130% of the correct bottleneck size. A slight underestimate in the bottleneck size for the case *N*^*T*^ = 100 was consistent with the extent of noise in sequencing; here and in all subsequent simulations a value of *C* = 200 was used, representing an extent of noise that is readily achievable from short read sequence data [52, 54]. In our inferences, while gross differences in bottleneck size can be identified, a high level of precision is difficult to obtain from sequence data alone.

#### Inference of population bottleneck sizes under selection for transmissibility

**Fig 5:**
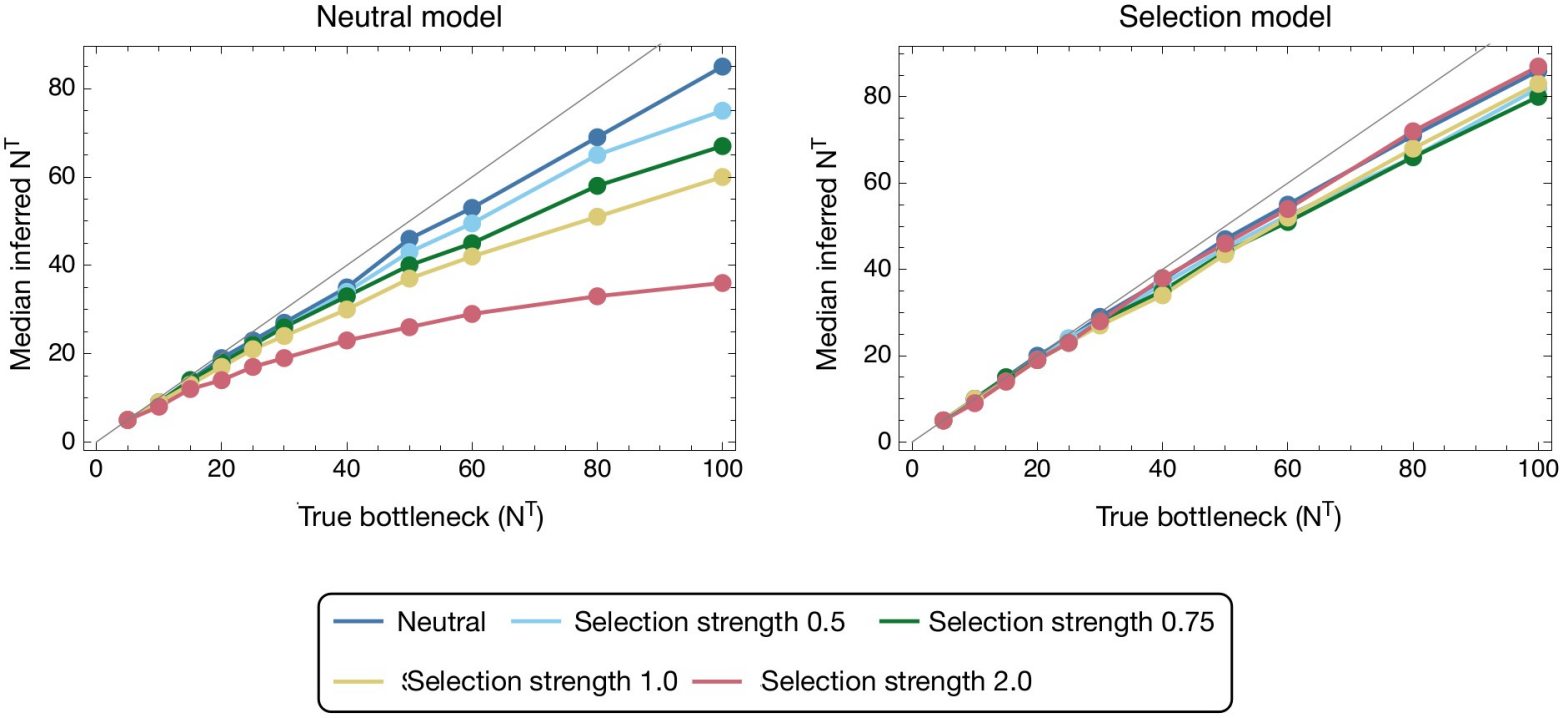
Median inferred bottleneck size from data simulating transmission with a single locus under selection of magnitude *σ* ∈ {0, 0.5, 0.75, 1.0, 2.0}. Inferences were made using either a neutral inference model, in which the effect of selection was assumed to be zero, or a model incorporating selection, which allowed the presence of selection to be inferred. Median inferences are shown from 200 simulations for each data point.

Inferences of bottleneck size showed a systematic underestimate of the bottleneck when selection affected a transmission event, but a method neglecting selection was used in the inference procedure (Fig 5). Simulations were conducted in which an allele at the third of five polymorphic loci in the HA segment of a simulated influenza virus increased the transmissibility of the virus according to a selection coefficient *σ*; this model of selection was applied for all subsequent simulations. In our simulations a value of *σ* = 1 is equivalent to a change in the frequency of a variant from 50% to 73% in a single transmission event. The relatively strong magnitudes of selection considered reflect the short period of time (a single generation) over which selection for increased transmissibility can act and the relatively small number of viruses likely to be involved in a transmission event.

Inferences of population bottleneck were conducted using a neutral inference method, and with a model in which selection was not constrained to be zero. In the first case, ignoring selection led to an underestimation of the true bottleneck size by an amount which increased according to the magnitude of selection for transmissibility. Selection during transmission produces a shift in the expected composition of the viral population; if this shift is interpreted as occurring solely due to a finite bottleneck a tighter bottleneck, inducing a larger stochastic change in the population, is inferred. This understanding explains the more pronounced underestimates achieved at larger bottleneck sizes; larger bottlenecks produce smaller stochastic changes in the population relative to the change induced by selection. When the full version of our model was run, allowing for a consideration of selection effects, the median bottleneck inferred from data under selection resembled that inferred from neutral data; the small shortfalls in the inference from neutral data are here explained by the influence of noise.

Calculations performed for data describing multiple replicate transmission events gave similar inferred transmission bottlenecks to those obtained from single replicates. In each case sets of three replicate transmission events were simulated, each event involving the transmission of virus between a distinct pair of hosts. Simulating the use of a consistent inoculum, our transmitted populations shared a common set of polymorphic loci in each segment. Median inferred values are shown in S3 Fig. Full results describing the range of inferred bottleneck sizes from both one- and three-replicate populations are shown in S4 to S7 Figs.

#### Identification of variants under selection

**Fig 6:**
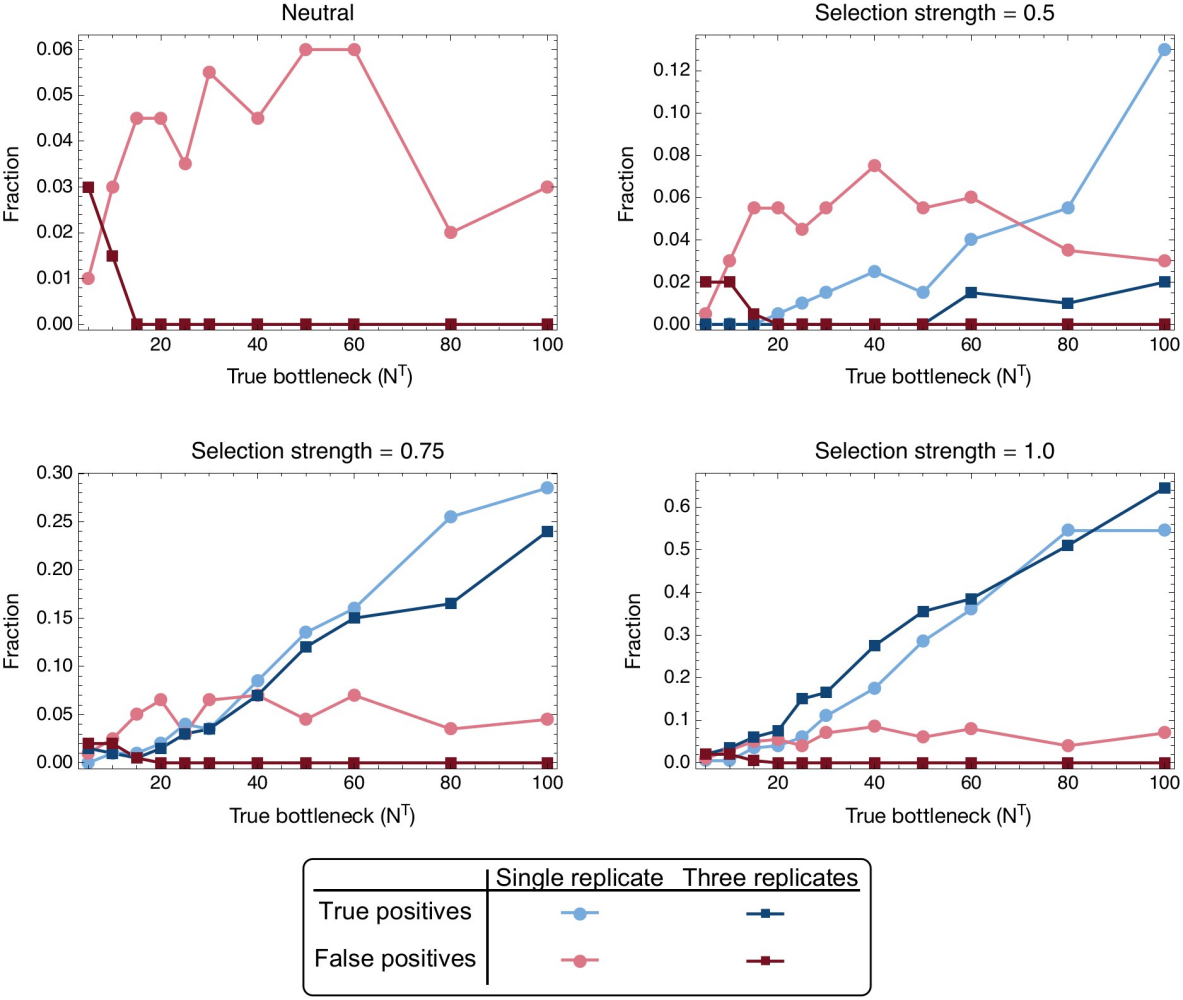
True and false positive rates of selection inference from 200 simulations of transmission events from single- and three-replicate systems in which a single variant was under selective pressure for increased transmissibility of *σ* ∈ {0, 0.5, 0.75.1.0}. True positives were defined as inferences for which selection was inferred for the selected locus in a system; false positives were defined as inferences for which selection was inferred at any neutral locus or for multiple neutral loci in the system.

In contrast to measures of diversity, which attempt to associate selection with a gene or segment of a virus, our method was able to correctly identify specific variants conferring increased transmissibility. Success was more often achieved in cases for which selection was relatively strong and the transmission bottleneck was relatively large (Fig 6). Our process for distinguishing selection from neutrality (Fig 2C) can be tuned to identify a greater number of true variants under selection at the cost of making a greater number of false positive calls; here a conservative approach to identifying selection was applied. We evaluated populations in which a single locus was under selection, evaluating our potential to identify the variant. Under this approach we retained a false positive rate (inference of selection at an unselected locus) of 8% or less across the systems tested. Where a single variant was under a lower magnitude of selection (*σ* ≤ 0.5), correctly identifying sites under selection was very difficult, though as selection became stronger (*σ* ≥ 1) loci under selection could be identified with greater accuracy. Where selection existed the potential for it to be identified was greater at larger bottleneck sizes. These results can again be understood with respect to the dynamics of the system. The bottleneck has a stochastic effect on the population of a magnitude inversely related to the number of viruses transmitted. Inferring the presence of selection requires the identification of changes in the population going beyond what would be expected under neutrality, biasing the population in the direction of the selected allele or alleles. However, stochastic effects can by chance distort the population in one direction or another by more than the expectation; this leads to false inferences of selection. Genuine changes resulting from selection become easier to identify when the changes are themselves larger (stronger selection) or where the magnitude of the stochastic effect is reduced (higher *N*^*T*^). While data from multiple replicate simulations made little difference to the inferred bottleneck size (see above), such data led to a more dramatic change in these results, with the false positive rate falling to zero for bottlenecks with *N*^*T*^ ≥ 20. The power of replicate experiments arises from the lower probability that stochastic effects will impose a consistent pattern of change upon multiple populations. While a larger-than-expected stochastic change in the frequency of a variant may occur in one system, leading to a false positive inference of selection, it is unlikely that the same pattern would recur across multiple replicates. While the inference of selection for transmissibility is not easy, the use of replicate experiments is of considerable value in this task; while, under our conservative approach, not all variants truly under selection were identified, those which were identified from replicate data were almost universally true positive calls.

#### Estimating the magnitude of a selected variant

**Fig 7:**
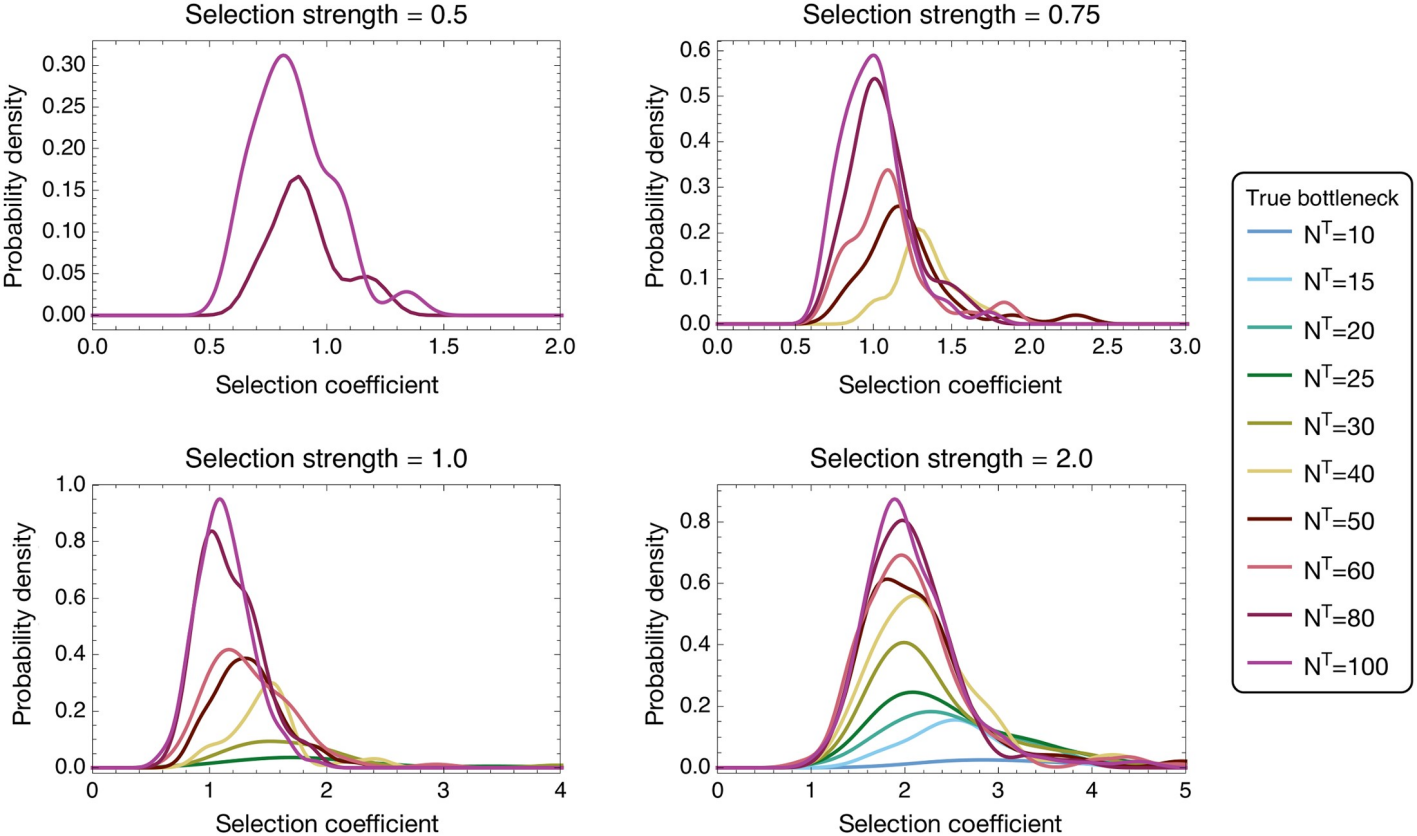
Probability distributions of inferred selection coefficients from 200 simulations of transmission events with selective pressures *σ* ∈ {0.5, 0.75, 1.0, 2.0}. Distributions were constructed for bottleneck values where the inference of selection resulted in a true positive rate for identifying selected variants of above 5%. Smooth kernel distributions were computed using a Gaussian kernel function defined on (0, 10) and Silverman’s rule of thumb [59, p. 48] for the bandwidth size. Distributions were scaled such that their integral across the kernel range equalled the true positive rate.

Given the correct identification of selection acting for a specific variant, the inferred magnitude of selection was marginally overestimated, with an increased overestimate at smaller values of the transmission bottleneck *N*^*T*^ (Fig 7). The mixture of deterministic and stochastic changes in the population explains this phenomenon; the population after transmission is equal to its expected value plus some stochastic change. In the event that the stochastic change is aligned with the direction of selection, the presence of selection is more likely to be inferred, while the additional change in that direction will give an overestimate of selection. Conversely, if the stochastic change is in a direction opposed to the influence of selection, the presence of selection is less likely to be inferred. Thus, selection was disproportionately inferred to exist when stochastic changes in the population led to an overestimate of its magnitude. Inferences conducted on sets of replicate transmission events produced more accurate and more precise estimates of selection. For example given a bottleneck of *N*^*T*^ = 100 and a true strength of selection of 0.75, the mean inferred selection from a single replicate was 1.00 with variance 0.040, while the mean inferred selection from three replicates was 0.90 with variance 0.010. (S8 Fig).

#### The biology of within-host viral growth may affect the inference of a transmission bottleneck

Comparing our approach with a previous inference method, we found that the biology underlying within-host viral growth can significantly affect the inferred population bottleneck. In so far as previous population genetic models have not accounted for the presence of selection or noise in sequencing data (beyond binomial variance) we applied methods to data describing neutral transmission between a single pair of hosts with error-free sequencing of samples. The method of Poon et al. [14] is explicitly defined across multiple transmission events so cannot be used to evaluate single transmission events. For this reason comparison was performed with the method described by Leonard et al. [12]; this recent and well-cited approach, which infers a transmission bottleneck based on allele frequency change in a manner that accounts for within-host viral growth, provides a useful benchmark for comparison.

**Fig 8:**
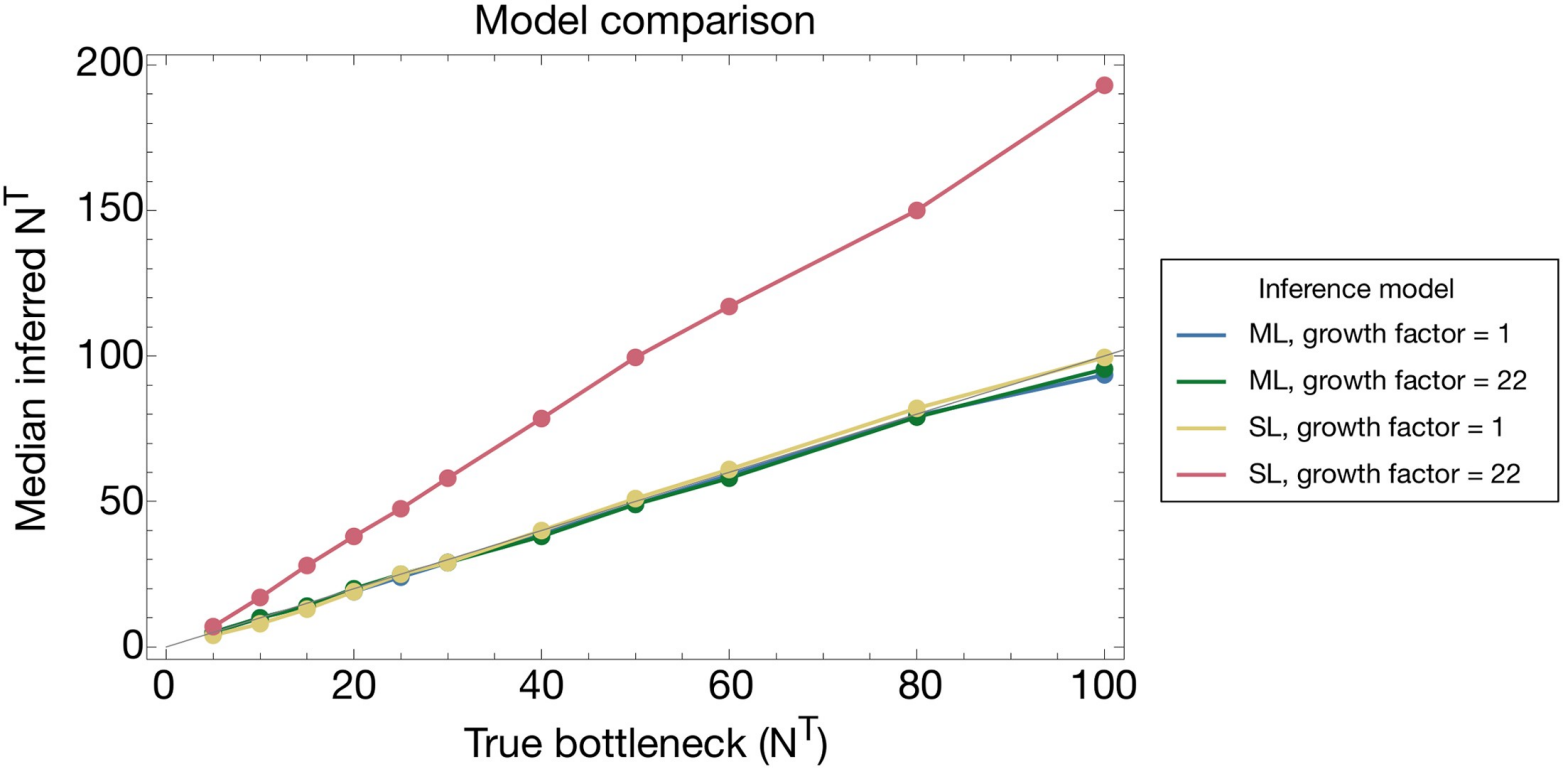
Median inferred bottleneck size from data simulating neutral transmission with the viral population undergoing either a single- or 22-fold increase in population size during within-host replication. Inferences were made using our approach (termed ML, for multi-locus method), which allows for specifying different growth factors, and the method of Leonard et al. [12], (termed SL, for single-locus method). Each datapoint represents the median bottleneck, calculated over 200 replicate simulations.

Comparison of the two methods showed our approach to have an increased flexibility to obtain correct inferences of population bottleneck size across a range of biological models of within-host growth. By default, our simulation model describes genetic drift during the within-host growth of the viral population as a single generation of replication, according to a Wright-Fisher population model with effective population size *gN*^*T*^, where *g* is nominally the growth rate of the population; our inference framework was set to match the generative model (Fig 8). At a growth factor of 1, both methods correctly inferred the size of the population bottleneck. However, at our default growth factor of 22 (based upon experimental results in influenza [60]), the method of Leonard et al., inferred a bottleneck size roughly double the correct value while our model was close to being correct.

This result highlights the need to correctly account for within-host growth during the inference of a transmission bottleneck. If too much of the difference between the populations observed before and after transmission is accounted for by within-host genetic drift, the inferred bottleneck will be too high. By contrast, if not enough of this difference is accounted for as drift, the inferred bottleneck will be too low. In the approach of Leonard et al., the accounting made for genetic drift accounts for a variance equivalent to that incurred in a Wright-Fisher step of size *N*^*T*^, that is, with *g* = 1 (personal correspondance, Daniel Weissman). Their method thus obtains a correct inference under these circumstances but reports false results if the assumed within-host growth model is not correct. Further details of the derivation of our within-host growth model are presented in the Methods section.

### Application to an experimental dataset

We applied our approach to an influenza transmission dataset obtained by Watanabe et al. [61] and subsequently analysed by Moncla et al. [25]. This dataset provides high-resolution, whole-genome sequence data describing both the within-host evolution, and airborne transmission, of a 1918-like influenza virus, that became transmissible upon introduction of three key mutations, PB2 E627K, HA E190D and G225D. This three-mutant strain was denoted ‘HA190D225D’ and successfully transmitted in one of three ferret transmission pairs. Isolation and subsequent growth in MDCK cells of viruses from the contact ferret of the successful transmission led to the generation of the ‘Mut’ strain, which transmitted in two of three instances. A previous analysis of these data using linked variants on the HA segment identified an increase in the diversity of the viral population during within-host growth, and respectively ‘loose’ and ‘stringent’ bottlenecks in the transmission of the two strains. In the transmission of the Mut strain, the fixation of sequence variants, potentially due to selection, was observed, while the observation of two out of three, rather than one out of three, successful transmissions suggested that the Mut virus may have evolved increased fitness for infection. Within and between hosts, segment-wide and localised measures of synonymous and non-synonymous sequence diversity *π* were used to assess the presence or absence of selection, leading to the conclusion that selection affected the system during transmission of the ‘Mut’ strain.

**Fig 9:**
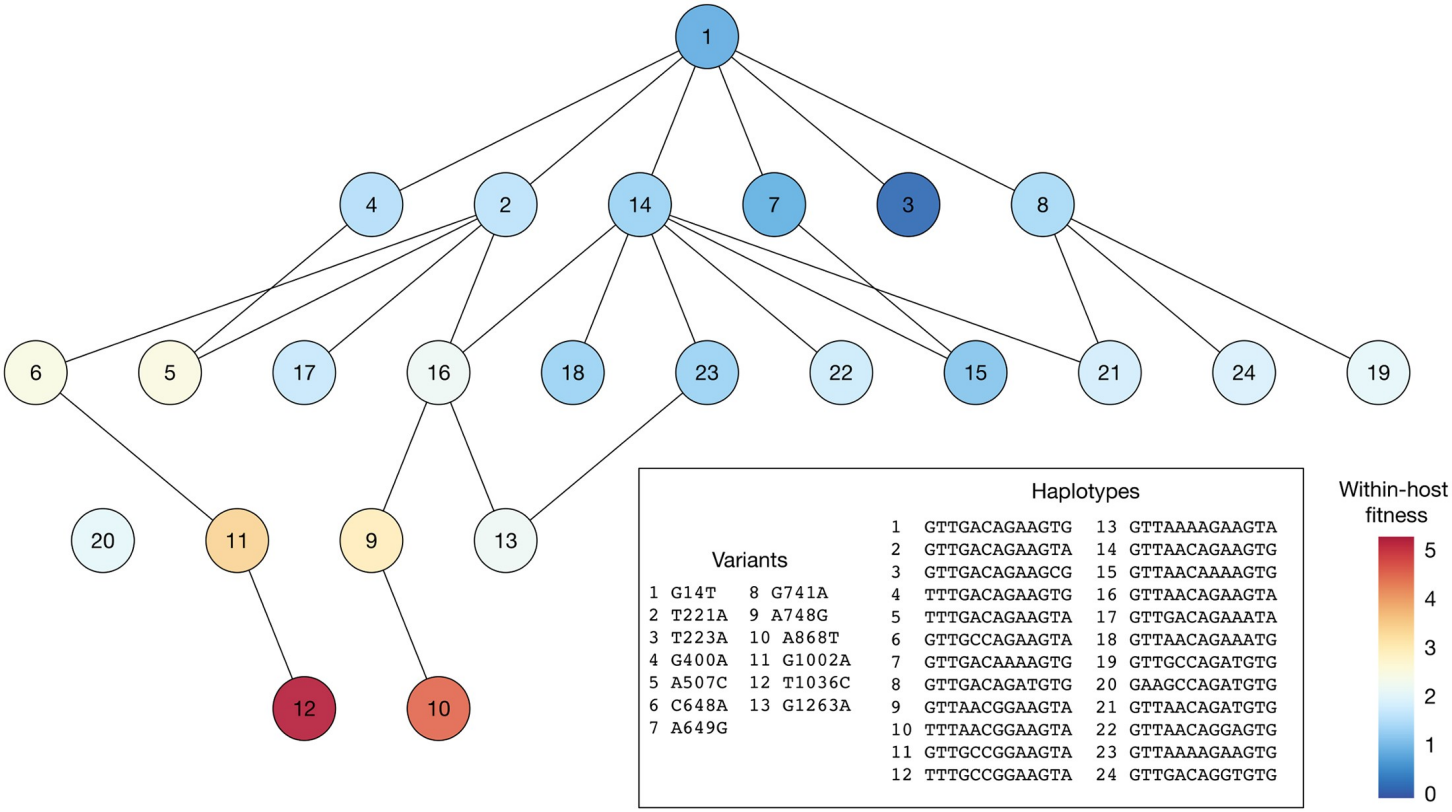
Inferred fitness landscape for within-host growth using data from the HA190D225D dataset. Viral haplotypes for which the inferred frequency rose above 1% in at least one animal are shown. Lines show haplotypes separated by a single mutation.

In our study, data from serial samples from the within-host populations were used to infer a fitness landscape describing the within-host growth of the virus for each of the two experimental populations. Using a previously published approach [52] we inferred the presence of non-neutral change in the population in seven out of eight segments in the combined HA190D225D population, and in four out of eight segments in the combined Mut population. The inference of positive selection acting for multiple non-consensus viral haplotypes in the HA segment (Fig 9) explains the increase in sequence diversity previously observed in these data. Further results are shown in S9 and S10 Figs.

**Fig 10:**
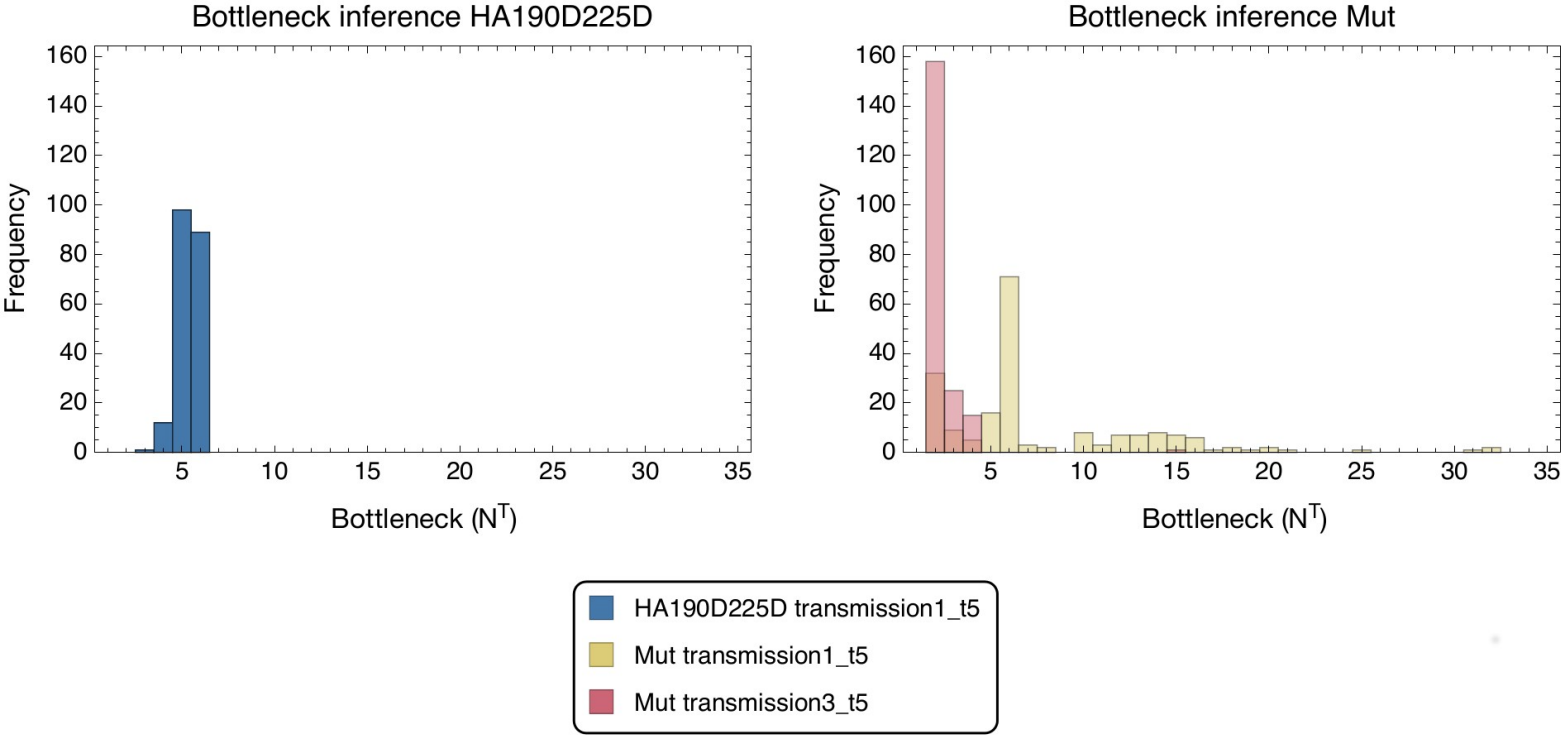
Histograms of bottleneck inferences for HA190D225D and Mut transmission pairs from 200 analysis seeds. A replicate inference method was employed for the Mut transmission pairs such that a common fitness landscape was imposed. The Mut transmission pairs may take different bottleneck values and have been plotted as an overlapping histogram. Bottleneck inferences larger than *N*^*T*^ = 35 have been omitted for clarity.

Applying our inference framework to the data identified narrow transmission bottlenecks in each case (Fig 10). In each of our calculations a set of statistical replicate inferences was produced, corresponding to different potential reconstructions of the population ***q***^*B*^ from the sequence data (see Methods). Within the HA190D225D population, our estimated bottlenecks ranged from 3 to 6, with a median bottleneck size of 5, while for the Mut calculations, our bottlenecks ranged from 2 to 127 and 2 to 61, with medians of 6 and 2 respectively. As such, no clear evidence was found that the HA190D225D transmission involved a greater number of particles than the Mut transmissions. Given the inclusion of the inferred within-host selection *S*^*G*^, no evidence was found for the existence of variants making the virus more or less transmissible, with selection being inferred in only a small number of the replicate calculations (S11 Fig). Increasing the frequency cutoff at which variants were included in the calculation led to small decreases in the inferred bottleneck sizes (S12 Fig).

## Discussion

We have here presented an approach for jointly inferring a population bottleneck size and selection for differential transmissibility from viral sequence data describing a transmission event. While basic sampling approaches to bottleneck inference have been improved by an accounting for drift during within-host viral growth [7, 12–14], our approach additionally accounts for noise in genome sequence data, exploits partial haplotype data available from short-read sequencing, and separates the influence of a finite bottleneck from that induced by selection for increased transmissibility. In multiple studies, the transmission bottleneck has been found to be narrow during natural viral spread between hosts [62]. While acknowledging previous evidence for the existence of small transition bottlenecks in viral systems, we here note that a failure to account for selection and noise in the transmission process can decrease the bottleneck that is inferred from sequence data. Our approach is suitable for the analysis of acute infectious diseases such as influenza on the basis of a small number of observed transmission events; we note that where more substantial diversity is present in a within-host viral population, or where data are available from a large number of hosts in an outbreak, phylogenetic methods of evolutionary inference become of increasing value [63–65].

Applied to the analysis of data from a recent evolutionary experiment, our approach provides a greater precision in the inference of evolutionary statistics, leading to an alternative explanation for the data observed. Where data have previously been interpreted as implying differential transmission bottlenecks between strains, our approach infers bottlenecks of similar sizes ranging from 2-6 viruses. Furthermore, where evidence has been interpreted to suggest a differing extent of transmissibility between strains, our approach attributes changes in the composition of the population to a mixture of stochastic effects and selection for increased within-host adaptation. Our result does not prove the absence of differential transmissibility among the viruses involved in this study; at the bottleneck size we inferred, selection is very hard to identify even where it does influence transmission. Rather, our claim is that under a parsimonious analysis of the data, apparent evidence for increased viral transmissibility can be explained by other evolutionary factors.

Our study shows that the identification of variants conferring increased viral transmissibility is difficult when the number of transmitted viral particles is small. While improvements to our method may be achievable, this difficulty is fundamentally rooted in the nature of a transmission event; where a low number of virions are transmitted, the influence of stochastic processes becomes large, with variants fixing during transmission in a manner that cannot be distinguished from a selective sweep. The potential to infer the presence of selection increases at larger population size and given a greater number of replicate transmission events. However the amount of data required to make a statistically robust identification of a variant increasing viral transmissibility may be large. We note that, unlike more general inferences of selection from changes in viral diversity, our approach evaluates selection in terms of specific variants conveying an advantage or disadvantage for transmission. Where broad measures of diversity are calculated across segments of a genome, the background of genetic diversity across a large number of positions may be hard to separate from changes at individual positions under the action of selection.

In the light of our study, we propose that the term used in some analyses of viral transmission, of a ‘selective bottleneck’ is ambiguous, failing on the one hand to distinguish changes in a population arising from selection and those occurring through stochastic change in the population, and on the other to distinguish between selection for more rapid within-host replication or for inherent viral transmissibility. While selection may act differently for these latter two phenotypes [55], their respective influences are intrinsically hard to separate when an infection is sparsely sampled. In our analysis the completeness of the collected data, covering both within-host adaptation and between-host transmission, was necessary to evaluate the cause of evolutionary change.

Our study provides some insight into the potential for inferring transmissibility using small animal experiments. One approach to exploring transmissibility (in influenza virus) has been the comparison, for different viruses, of the proportion of distinct animal pairs between which transmission occurs [66]. The statistical significance achievable in these studies is limited by the number of animal pairs that can be examined [67–69]. Furthermore, the comparison between one genotype and another may be confounded by viral heterogeneity, whereby each population contains a cloud of genetic diversity [24, 70]. As we have shown, data from replicate transmission events leads to an improved ability to infer selection, in particular by reducing the false positive rate of inference and by increasing the accuracy in inferred selection coefficients. We note, however, that the number of viral particles transmitted in each event is key in determining whether increased transmissibility can be identified; where a transmission bottleneck is narrow it is inherently difficult to identify selection against a background of the large changes in the population induced by stochastic effects. Where transmission bottlenecks are small, a large number of replicates might be needed to make statistically well-supported inferences of increased transmissibility. Applications of our method to simulated data could be used to gain an insight into what might be obtained from a particular experimental setup.

In some situations, neutral markers or molecular barcodes may be added to a viral population [5, 30]; without providing an estimate of selection, sequencing these markers before and after transmission can give a precise estimate of the population bottleneck. While our method does not require the presence of such markers, its adaptation to include marker data would likely be straightforward, including in a calculation a further probabilistic term constraining the bottleneck size. Inference of selection for transmissibility could then be conducted under this constraint; the combination of whole-genome sequence data with such information could prove powerful for the study of viral transmission.

While we have here considered the transmission of influenza virus, very few steps of our approach would need to be altered for the method to be applied to another viral population. As detailed in the Methods section, it is only in accounting for genetic drift in the within-host growth of the virus that we make approximations relying on biological knowledge of the influenza virus; an alternative accounting for within-host expansion could be used. A second key assumption in the inference of selection is the existence of regions of the virus separated from each other by recombination or reassortment. This assumption would be preserved in some other viruses, as noted in observations of within-host HIV evolution [71], if not for all influenza populations [72]. Where a viral genome did not exhibit recombination, and only a single transmission event was observed, the neutral version of our method could be applied; in this context our accounting for haplotype structure and sequencing noise in transmission represents an advance over methods which ignore these factors.

Viral transmission is a critical component of disease and a key factor in viral evolution. In outlining a novel framework for the interpretation of data from viral transmission events we hope to bring a greater clarity to the population genetic theory of how these events operate and a greater power in the interpretation of experimental data, so as to engender a greater understanding of this important topic of research.

## Methods

### Notation and qualitative overview

We describe the viral population as a set of haplotypes, with associated frequencies, that changes in time during a transmission event. Given a number of (possibly non-consecutive) loci of interest in the viral genome, the set of haplotypes ***h*** = {*h*_*i*_} describes a set of sequences having specific nucleotides at these loci. Within a viral population of finite size, the number of viruses with each haplotype *h*_*i*_ is described by the vector ***n*** = {*n*_*i*_}. Frequencies of each haplotype within the population are denoted by the vector ***q*** = {*q*_*i*_}, while observations of the population collected via sequencing are denoted by the vector ***x*** = {*x*_*i*_}, where *x*_*i*_ is the number of sampled viruses with haplotype *h*_*i*_.

The transmission event is now described according to the framework outlined in Fig 2. A population of viruses ***q***^*B*^ undergoes transmission with some bottleneck *N*^*T*^, creating a founder population with haplotype frequencies ***q***^*F*^ in the recipient. Selection influencing this transmission process is described by the function ***S***^*T*^(***q***), which changes the frequency of haplotypes according to the relative propensity of each haplotype to transmit. For example, selection may favour the transmission of viruses containing a specific genetic variant, increasing the expected proportion of viruses with this variant in the founder population. Within the host, the viral population grows rapidly in number to create the population ***q***^*A*^. During this growth process, genetic drift affects the population in a manner according to the effective population size *N*^*G*^. Observations of the system are made via genome sequencing of samples collected before and after transmission, and are denoted ***x***^*B*^ and ***x***^*A*^ respectively; the total numbers of sequence reads in each are denoted *N*^*B*^ and *N*^*A*^. Given the observations ***x***^*B*^ and ***x***^*A*^, we wish to estimate the size of the population bottleneck *N*^*T*^ and the extent of selection for transmissibility *S*^*T*^.

During the process of growth between ***q***^*F*^ and ***q***^*A*^, the population may be influenced by selection for within-host growth; this acts independently of selection for transmissibility [73], and is described by the function *S*^*G*^(***q***), which changes the frequencies of haplotypes according to their relative within-host growth rates. Selection for within-host growth is challenging to separate from selection for transmissibility; we here estimate this parameter independently from the transmission event itself.

Where we consider multiple replicate transmission events, we assume that each transmission has its own transmission bottleneck *N*^*T*^; different numbers of viruses may infect different hosts. However, we assume that selection operates consistently between hosts; a variant which makes a virus grow more efficiently in one host does the same in another.

### Likelihood framework

As the observations ***x***^*B*^ and ***x***^*A*^ are conditionally independent given ***q***^*B*^, the joint probability of the system may be written as a product of individual probabilities

$$\begin{array}{c} P\left(x^{B},x^{A}|q^{B},\theta \right)=P\left(x^{B}|q^{B}\right)P\left(x^{A}|q^{B},\theta \right) \end{array}$$ (1)

where *θ* represents the remaining variables in the system upon which only ***x***^*A*^ depends.

As an approximation to this likelihood, we split the inference into two calculations, first calculating a maximum likelihood for ***q***^*B*^ given ***x***^*B*^, then inferring the transmission event from ***x***^*A*^ given ***q***^*B*^. Noting the potential uncertainty in the inference of ***q***^*B*^, we introduce a variance component so that ***q***^*B*^ may be regarded as a random variable rather than a fixed quantity. The process of breaking up the inference process greatly reduces the computational time required for our approach, without considerable cost to the accuracy of the results. Splitting the likelihood in this manner, and marginalising over unknown quantities, the likelihood can be written generically as

$$\begin{array}{l} \begin{array}{l} L\left(N^{T},S^{T}|x^{B},x^{A},N^{G},S^{G}\right)=\underset{x^{B}\;\text{component}\;}{\underset{︸}{\int P\left(x^{B}|q^{B}\right)\;P\left(q^{B}\right)\;\text{d}q^{B}}}\;\;\times \\ \underset{x^{A}\;\text{component}}{\underset{︸}{\int P\left(x^{A}|q^{A}\right)[\int P\left(q^{A}|N^{G},S^{G},q^{F}\right)[\int P\left(q^{F}|N^{T},S^{T},q^{B}\right)\;P\left(q^{B}\right)\;\text{d}q^{B}]\text{d}q^{F}]\;\text{d}q^{A}}} \end{array} \end{array}$$ (2)

The first component of this likelihood, corresponding to the initial observation of the system, ***x***^*B*^, represents a straightforward sampling of the system, drawing from a collection of viral haplotypes. Such a process can be modelled using a multinomial distribution. However, as is well known [54], next-generation sequence data are error-prone, such that less information is contained within the sample than would be contained in a multinomial sample of equivalent depth. A Dirichlet multinomial distribution may be used to capture this reduction of information [52, 57], such that

$$\begin{array}{c} P\left(x^{B}|q^{B}\right)=\frac{\Gamma (N^{B}+1)}{\prod_{i}\left(x_{i}^{B}+1\right)}\frac{\Gamma (\sum Cq_{i}^{b})}{\Gamma (\sum x_{i}^{B}+Cq_{i}^{B})}\prod_{i}\frac{\Gamma (x_{i}^{b}+Cq_{i}^{B})}{\Gamma (Cq_{i}^{B})} \end{array}$$ (3)

where *C*, which alters the variance of the distribution, characterises the extent of noise in the data. The parameter *C* can be estimated given independent observations of identical parameters, such as haplotype or single allele frequencies; in the application to experimental data, time-resolved variant frequencies derived from the sequence data were used for this purpose [52].

Considering the second component of the likelihood, the expression *P*(***x***^*A*^|***q***^*A*^) may be calculated in the same manner as in Eq 3 dependent upon the haplotype frequencies ***q***^*A*^. The remaining parts of this component can also be described as sampling events. A sample of the population in the donor animal transmits to the recipient, generating a founder population. The founder population multiplies within the host, with offspring being sampled from the founder population ***q***^*F*^ to generate the final population ***q***^*A*^. The ***x***^*A*^ component thus represents a compound of multiple sampling events. We will go on to describe the calculation of both components of the likelihood function. However, we first need to consider how selection is incorporated into our model.

#### Excursus: Modelling selection

Within our model, the functions describing selection are potentially complex, each having a number of parameters equal to the number of haplotypes in the system. In common with previous approaches to studying within-host influenza evolution [74] we adopt a hierarchical model of selection whereby the fitness of a haplotype is calculated from a set of one- or multi-locus components, describing the advantage or disadvantage of a specific nucleotide, or nucleotides, at a single locus or set of loci. A model selection process is then used to identify the most parsimonious explanation of the data.

Formally, we denote the *j*^*th*^ component of the haplotype *h*_*i*_ as *h*_*ij*_, with *h*_*ij*_ ∈ {*A*, *C*, *G*, *T*}. In a fitness model, a parameter is defined as the pair of values (*s*_*k*_, *g*_*k*_), where *s*_*k*_ is a real number, denoting the difference in fitnesses of individuals with and without the allele [75], and *g*_*k*_ is a vector of components *g*_*kj*_ ∈ {*A*, *C*, *G*, *T*, −} denoting the haplotypes to which this selection applies. We now define

$$\begin{array}{c} g_{k}\cdot h_{i}=\prod_{j}g_{kj}\times h_{ij} \end{array}$$ (4)

where

$$\begin{array}{c} g_{kj}\times h_{ij}=\{\begin{array}{cc} 1, & \text{if}\;g_{kj}=h_{ij}\\ 1, & \text{if}\;g_{kj}=-\\ 0, & \text{if}\;g_{kj}\neq -,g_{kj}\neq h_{ij} \end{array} \end{array}$$ (5)

The fitness of a haplotype *h*_*i*_ is then given as

$$\begin{array}{c} w_{i}=\exp(\sum_{k}s_{k}\left(g_{k}\cdot h_{i}\right)) \end{array}$$ (6)

where the sum is calculated over all fitness parameters *k*. To give an example, a single-locus fitness parameter would have a single element of *g*_*k*_ that was either A, C, G, or T. Supposing this element to be at position *j*, it would convey the fitness advantage *s*_*k*_ to all haplotypes with the given nucleotide at position *j* in the genome.

#### Selection in a transmission event

Selection is incorporated into the transmission event from donor to recipient by representing this event as a biased sampling process. As we are not considering data here, noise is not an issue. We therefore model the population ***q***^*F*^ as arising via a multinomial sampling process of depth *N*^*T*^ from a set of genotypes with frequencies *S*^*T*^(***q***^*B*^), where *S*^*T*^ represents the role of selection in the transmission event. We write

$$\begin{array}{c} P\left(q^{F}|q^{B},N^{T},S^{T}\right)=\frac{N^{T}!}{\prod_{i}n_{i}^{F}!}\prod_{i}{\left(S^{T}\left(q^{B}\right)\right)}_{i}^{n_{i}^{F}} \end{array}$$ (7)

where

$$\begin{array}{c} {\left(S^{T}\left(q^{B}\right)\right)}_{i}=\frac{w_{i}^{T}q_{i}^{B}}{\sum_{i^{\prime }}w_{i^{\prime }}^{T}q_{i^{\prime }}^{B}} \end{array}$$ (8)

defines a distorted population based on the haplotype fitnesses $w^{T}=\left\{w_{i}^{T}\right\}$, representing the relative propensity of each haplotype *h*_*i*_ for transmission. We note here that $q_{i}^{F}=\frac{n_{i}^{F}}{N^{T}}$, where the vector ***n***^*F*^ describes the number of copies of each haplotype in the founder population.

#### Selection during within-host growth

From the founding of an infection in the recipient, the viral population grows to the point at which data are collected for sequencing, under the influence of both genetic drift and selection. Selection for within-host growth is modelled by the function *S*^*G*^, identical in form to *S*^*T*^. We note that neglect of this term could distort the inferred value of *S*^*T*^; given only data collected before and after transmission the two terms cannot be separated. However, where samples have been collected at distinct times from one or multiple hosts, it is possible to make an independent estimate of *S*^*G*^ [52], such that the two forms of selection can be discriminated. We here incorporate within-host selection into our derivation; the absence of such selection is then represented as a special case of our model.

Concerning genetic drift, we note that the number of viruses in a host grows rapidly, with experiments suggesting that a single infected cell can produce between 10^3^ and 10^4^ viruses [76]. However not every such virus is viable, and one estimate has put the number of naive cells infected by an infected influenza cell at 22 [60]. By default we here approximate the within-host growth of the virus as a single multinomial draw, compressing growth to a single round of sampling, with the variance effective population size *N*^*G*^ = *gN*^*T*^. By default we set the growth factor *g* to be equal to 22. This approach is distinct from the branching process used in another estimate of bottleneck size [72]; our assumption that viruses infect different cells in the host, with competition between viruses occurring after the release of viruses from cells, leads to a Wright-Fisher-type population model, in which the rapid growth of the viral population leads to a smaller amount of genetic drift than inferred in that model. We note that our method can be extended to incorporate multiple rounds of within-host viral growth; a first approximation would be to reduce *g* to match the effective variance in frequencies induced by repeated rounds of growth. A fuller solution has been implemented in our code; full details are provided in S1 Text.

#### Approximation of the likelihood function

We now turn to calculating the likelihood function of Eq 2. On account of the discrete nature of the multinomial distribution, the integrals present in this equation may be written as sums over all possible outcomes of the multinomial sampling processes represented by the different potential values of ***q***^*F*^ and ***q***^*A*^. However, in realistic cases, where there might be multiple haplotypes present, the number of possible outcomes grows combinatorially with *N*^*T*^, making this calculation intractable. Instead we consider a continuous approximation in which the random variables of the model (Fig 2A) are represented by multivariate normal distributions, each defined by a mean and covariance matrix. By ignoring higher order moments, we may then calculate the individual components of the system (Eq 2) by appealing to a moments based approach for the evaluation of integrals arising from marginalisation over unknown variables. This step follows multiple previous approaches to time-resolved data, in which moments-based approximations have been used to simplify the propagation of evolutionary models [36, 77–79].

The haplotype frequency vector ***q***^*B*^ is unknown and must be determined from the available data. We denote the mean of the distribution of ***q***^*B*^ as ***μ***^*B*^ and its covariance matrix by Σ^*B*^. Given a sampling depth *N*^*B*^ and a dispersion parameter *C*, we describe ***x***^*B*^ as a distribution with mean and variance derived from the Dirichlet multinomial [80]:

$$\begin{array}{c} \text{E}[x^{B}|q^{B}]=N^{B}q^{B} \end{array}$$ (9)

and

$$\begin{array}{c} \text{var}[x^{B}|q^{B}]=(\frac{N^{B}+C}{1+C})\;N^{B}(\text{Diag}\left(q^{B}\right)-q^{B}{\left(q^{B}\right)}^{†})\equiv \beta N^{B}M\left(q^{B}\right) \end{array}$$ (10)

where $\beta =(\frac{N^{B}+C}{1+C})$, *M*(***q***) = Diag(***q***) − ***qq***^†^ and † indicates the transpose function.

The founder population ***q***^*F*^ is sampled from ***q***^*B*^. Its mean is given by the expression

$$\begin{array}{c} \text{E}\left[q^{F}|q^{B}\right]=S^{T}\left(q^{B}\right) \end{array}$$ (11)

and its variance by

$$\begin{array}{c} \text{var}\left[q^{F}|q^{B}\right]=\frac{1}{N^{T}}(\text{Diag}\left(S^{T}\left(q^{B}\right)\right)-S^{T}\left(q^{B}\right)S^{T}{\left(q^{B}\right)}^{†})\equiv \frac{1}{N^{T}}M\left(S^{T}\left(q^{B}\right)\right) \end{array}$$ (12)

arising from a multinomial sample of depth *N*^*T*^ and the selectively shifted frequencies *S*^*T*^(***q***^*B*^).

Similarly, the within-host growth process may be represented by a distribution with mean E[***q***^*A*^|***q***^*F*^] = *q*^*F*^ and variance $\text{var}\left[q^{A}|q^{F}\right]=\frac{1}{N^{G}}M(q^{F}$). As for the pre-transmission case, a Dirichlet multinomial likelihood with sampling depth *N*^*A*^, selectively shifted frequencies *S*^*G*^(***q***^*A*^) and dispersion parameter *C* may be used to model the sequencing of the population post-transmission. The resulting distribution can be approximated as a multivariate normal with mean

$$\begin{array}{c} \text{E}\left[x^{A}|q^{A}\right]=N^{A}S^{G}\left(q^{A}\right) \end{array}$$ (13)

and variance

$$\begin{array}{c} \text{var}\left[x^{A}|q^{A}\right]=(\frac{N^{A}+C}{1+C})\;N^{A}M\left(S^{G}\left(q^{A}\right)\right)\equiv \alpha N^{A}M\left(S^{G}\left(q^{A}\right)\right) \end{array}$$ (14)

where $\alpha =(\frac{N^{A}+C}{1+C})$ is defined for notational convenience.

Having established the above distributions, we are now equipped to carry out the relevant marginalisations (Eq 2) using the law of total expectation and the law of total variance. Starting with the pre-transmission compound distribution, the marginalisation over ***q***^*B*^ yields a mean of

$$\begin{array}{c} \text{E}\left[x^{B}\right]=\text{E}\left[\text{E}\left[x^{B}|q^{B}\right]\right]=\text{E}\left[N^{B}q^{B}\right]=N^{B}\mu^{B} \end{array}$$ (15)

and a variance of

$$\begin{array}{c} \begin{array}{cc} \text{var}(x^{B}) & =\text{E}\left[\text{var}\left[x^{B}|q^{B}\right]\right]+\text{var}\left[\text{E}\left[x^{B}|q^{B}\right]\right]\\ & =\text{E}[\beta N^{B}(\text{Diag}\left(q^{B}\right)-q^{B}{\left(q^{B}\right)}^{†})]+\text{var}\left[N^{B}q^{B}\right]\\ & =\beta N^{B}(\text{Diag}\left(\text{E}\left[q^{B}\right]\right)-\text{E}\left[q^{B}\right]E{\left[q^{B}\right]}^{†})+N^{B}(N^{B}-\beta )\text{var}\left[q^{B}\right]\\ & =\beta N^{B}M(\mu^{B})+N^{B}(N^{B}-\beta )\Sigma^{B} \end{array} \end{array}$$ (16)

These expressions characterise the ***x***^*B*^ component of the likelihood from Eq 2 in terms of a normal distribution. We identify values of ***μ***^*B*^ and Σ^*B*^ maximising this likelihood. The matrix Σ^*B*^ has dimensionality *k*^2^ where *k* is the number of haplotypes in the system, a number which may potentially be large. Accurately determining so many parameters from the available data is unrealistic. In preference to obtaining an ill-defined covariance matrix we make the approximation that the off-diagonal elements of Σ^*B*^ are zero, i.e. we disregard between-haplotype correlations in specifying the uncertainty in ***μ***^*B*^. We note that ignoring the variance component altogether results in an underestimation of the population bottleneck (S13 Fig).

Moving on to the post-transmission process, the marginalisation over ***q***^*B*^ results in a mean of

$$\begin{array}{c} \text{E}\left[q^{F}\right]=\text{E}\left[\text{E}\left[q^{F}|q^{B}\right]\right]=\text{E}\left[S^{T}\left(q^{B}\right)\right]\approx S^{T}\left(\text{E}\left[q^{B}\right]\right)=S^{T}\left(\mu^{B}\right) \end{array}$$ (17)

where in the penultimate step we used the first-order second-moment approximation to a vector function acting on a random variable. The law of total variance yields

$$\begin{array}{ll} \text{var}(q^{F}) & =\text{E}[\text{var}[q^{F}|q^{B}]]+\text{var}[\text{E}[q^{F}|q^{B}]]\\ & =\text{E}\left[\frac{1}{N^{T}}M(S^{T}(q^{B}))\right]+\text{var}\left[S^{T}(q^{B})\right]\\ & =\frac{1}{N^{T}}M(\text{E}[S^{T}(q^{B})])+\left(1-\frac{1}{N^{T}}\right)\;\text{var}[S^{T}(q^{B})]\\ & \approx \frac{1}{N^{T}}M(S^{T}(\text{E}[q^{B}]))+\left(1-\frac{1}{N^{T}}\right)\left({DS^{T}|}_{\text{E}\left[q^{B}\right]}\right)\;\text{var}[q^{B}]{\left({DS^{T}|}_{\text{E}\left[q^{B}\right]}\right)}^{†}\\ & =\frac{1}{N^{T}}M(S^{T}(\mu^{B}))+\left(1-\frac{1}{N^{T}}\right)\left({DS^{T}|}_{\mu^{B}}\right)\Sigma^{B}{\left({DS^{T}|}_{\mu^{B}}\right)}^{†} \end{array}$$ (18)

Note that ${\left(DS\right)}_{i}^{j}=\frac{\partial S_{i}}{\partial q_{j}}$ is the Jacobian matrix arising from the first-order second-moment approximation.

Marginalisation over ***q***^*F*^ yields a mean of

$$\begin{array}{c} \text{E}\left[q^{A}\right]=\text{E}\left[\text{E}\left[q^{A}|q^{F}\right]\right]=\text{E}\left[q^{F}\right]=S^{T}\left(\mu^{B}\right) \end{array}$$ (19)

$$\begin{array}{ll} \text{var}(q^{A}) & =\text{E}[\text{var}[q^{A}|q^{F}]]+\text{var}[\text{E}[q^{A}|q^{F}]]\\ & =\text{E}\left[\frac{1}{N^{G}}\left(\text{Diag}(q^{F})-q^{F}{\left(q^{F}\right)}^{†}\right)\right]+\text{var}[q^{F}]\\ & =\frac{1}{N^{G}}\left(\text{Diag}(\text{E}[q^{F}])-\text{E}[q^{F}]E{\left[q^{F}\right]}^{†}\right)+\left(1-\frac{1}{N^{G}}\right)\;\text{var}[q^{F}]\\ & \begin{array}{c} =\frac{1}{N^{G}}M\left(S^{T}(\mu^{B})\right)\\ +\left(1-\frac{1}{N^{G}}\right)\left(\frac{1}{N^{T}}M(S^{T}(\mu^{B}))+\left(1-\frac{1}{N^{T}}\right)\left({DS^{T}|}_{\mu^{B}}\right)\Sigma^{B}{\left({DS^{T}|}_{\mu^{B}}\right)}^{†}\right) \end{array}\\ & \begin{array}{c} =\frac{N^{T}+N^{G}-1}{N^{T}N^{G}}M\left(S^{T}(\mu^{B})\right)\\ +\frac{N^{T}N^{G}-N^{T}-N^{T}+1}{N^{T}N^{G}}\left({DS^{T}|}_{\mu^{B}}\right)\Sigma^{B}{\left({DS^{T}|}_{\mu^{B}}\right)}^{†} \end{array}\\ & \equiv \gamma M\left(S^{T}(\mu^{B})\right)+\delta \left({DS^{T}|}_{\mu^{B}}\right)\Sigma^{B}{\left({DS^{T}|}_{\mu^{B}}\right)}^{†} \end{array}$$ (20)

where in the last step we defined $\gamma =(\frac{N^{T}+N^{G}-1}{N^{T}N^{G}})$ and $\delta =\frac{N^{T}N^{G}-N^{T}-N^{G}+1}{N^{T}N^{G}}$.

Treating the integral over ***q***^*A*^ in a similar manner, we obtain by the law of total expectation

$$\begin{array}{c} \text{E}\left[x^{A}\right]=\text{E}\left[\text{E}\left[x^{A}|q^{A}\right]\right]=\text{E}\left[N^{A}S^{G}\left(q^{A}\right)\right]\approx N^{A}S^{G}(\text{E}[q^{A}])=N^{A}S^{G}(S^{T}(\mu^{B})) \end{array}$$ (21)

Analogously, the law of total variance yields

$$\begin{array}{c} \begin{array}{cc} \text{var}(x^{A}) & =\text{E}\left[\text{var}\left[x^{A}|q^{A}\right]\right]+\text{var}\left[\text{E}\left[x^{A}|q^{A}\right]\right]\\ & =\text{E}[\alpha N^{A}M(S^{G}(q^{A}))]+\text{var}\left[N^{A}S^{G}(q^{A})\right]\\ & =\alpha N^{A}(\text{Diag}(\text{E}[S^{G}(q^{A})]-\text{E}[S^{G}(q^{A})]\text{E}{\left[S^{G}(q^{A})\right]}^{†})\\ & \;+N^{A}(N^{A}-\alpha )\text{var}\left[S^{G}(q^{A})\right]\\ & \approx \alpha N^{A}(\text{Diag}(S^{G}(\text{E}[q^{A}])-S^{G}(\text{E}[q^{A}]){\left(S^{G}(\text{E}[q^{A}])\right)}^{†})\\ & \;+N^{A}(N^{A}-\alpha )\left(DS^{G}|{}_{E[q^{A}]}\right)\text{var}[q^{A}]{\left(DS^{G}|{}_{E[q^{A}]}\right)}^{†}\\ & =\alpha N^{A}M\left(S^{G}\left(S^{T}\left(\mu^{B}\right)\right)\right)+N^{A}(N^{A}-\alpha )(DS^{G}|{}_{S^{T}(\mu^{B})})\times \\ & \;\;(\gamma M(S^{T}\left(\mu^{B}\right))+\delta (DS^{T}|{}_{\mu^{B}})\Sigma^{B}{\left(DS^{T}|{}_{\mu^{B}}\right)}^{†}){\left(DS^{G}|{}_{S^{T}(\mu^{B})}\right)}^{†} \end{array} \end{array}$$ (22)

The above expressions represent mean and covariance matrices of multivariate normal distributions resulting from the evaluation of marginalisations in Eq 2. As such, the components of Eq 2 may be represented in a tractable form as the probability density functions of two multivariate normal distributions; The ***x***^*B*^ component has mean and covariance matrix as specified in Eqs 15 and 16, whilst the ***x***^*A*^ component has mean and covariance matrix as given in Eqs 21 and 22. Taken as a whole, this defines a likelihood for the transmission event given the data. As such, given an independent estimate of *S*^*G*^, and our estimated values for *μ*^*B*^ and Σ^*B*^, the maximum likelihood values of *N*^*T*^ and *S*^*T*^ may be inferred.

### Reversion to a discrete likelihood function

Given a mean and covariance matrix for the likelihood function, we can approximate the likelihood by the probability density function of a multivariate normal distribution. However, where the variance of this distribution is very small in one dimension, as can occur under an inference of very strong selection, the density function evaluated at a point can become arbitrarily large. For this reason a Gaussian cubature approach was used to calculate the integral of the final likelihood over the unit cube described by each observation ***x***, once optimisation had been completed. Approximate numerical integrals were calculated using the software package cubature [81].

### Extension to partial haplotype data

In the calculations above we made the implicit assumption that the observations ***x***^*B*^ and ***x***^*A*^ consist of sets of complete viral haplotypes ***h***_*i*_. However, short-read sequencing technologies generally result in sets of individual reads which only cover a subset of the genetic loci of interest; we refer to these reads as partial haplotypes. In this framework the data represents direct observations of partial haplotypes in the set $h^{P}=\left\{h_{1}^{\text{P}},\ldots ,h_{L}^{\text{P}}\right\}$, where each of the sets $h_{l}^{\text{P}}$ is a vector of haplotypes spanning a common subset of the loci spanned by the full haplotypes in ***h***. Population-wide observations of these partial haplotypes are then defined by $x^{\text{P}}=\left\{x_{1}^{\text{P}},\ldots ,x_{L}^{\text{P}}\right\}$ with $x_{l}^{\text{P}}=\left\{x_{li}^{\text{P}}\right\}$ where $x_{li}^{\text{P}}$ is the number of reads with haplotype $h_{li}^{\text{P}}$. As a result, the total number of observations must now be defined on the basis of each set of partial haplotypes, e.g. $N_{l}^{B,\text{P}}=\sum_{i}x_{li}^{\text{P}}$ is the total number observations of partial haplotypes in the set *l*. As each set of partial haplotype observations is independent of the others, we may reconstruct Eq 2 in the following terms:

$$\begin{array}{c} \log L\left(N^{T},S^{T}|x^{B},x^{A},N^{G},S^{G}\right)=\sum_{l}\text{log}L\left(N^{T},S^{T}|x_{l}^{B,\text{P}},x_{l}^{A,\text{P}},N^{G},S^{G}\right) \end{array}$$ (23)

Within this construction, bottleneck sizes and selection are conserved between partial haplotype sets, being evaluated at the full haplotype level. Each set of partial haplotype observations $x_{l}^{\text{P}}$ is considered as a sample drawn from a set of partial haplotypes with frequencies $q_{l}^{\text{P}}$, these frequencies being defined via a linear transformation of the full haplotype frequencies with matrix *T*_*l*_. For example, given the full haplotypes {AG, AT, CG, CT} and a set of partial haplotypes {A-, C-}, we have

$$\begin{array}{c} q_{l}^{\text{P}}=T_{l}q \end{array}$$ (24)

or more explicitly,

$$\begin{array}{c} \left(\begin{array}{c} q_{l1}^{\text{P}}\\ q_{l2}^{\text{P}} \end{array})=(\begin{array}{cccc} 1 & 1 & 0 & 0\\ 0 & 0 & 1 & 1 \end{array})(\begin{array}{c} q_{1}\\ q_{2}\\ q_{3}\\ q_{4} \end{array}\right) \end{array}$$ (25)

Thus, as described above, the calculation of transmission and within-host growth under selection can be performed at the level of full haplotypes, switching into partial haplotype space only to evaluate the likelihoods of the observations. Re-deriving the results of Eqs 15 and 16 for short-read sequence data, we find that the compound distribution for the ***x***^*B*^ component has mean

$$\begin{array}{c} \text{E}\left[x_{l}^{B,\text{P}}\right]=N_{l}^{B,\text{P}}T_{l}\;\mu^{B} \end{array}$$ (26)

and variance

$$\begin{array}{c} \text{var}\left(x_{l}^{B,\text{P}}\right)=\beta N_{l}^{B,\text{P}}M(T_{l}\mu^{B})+N_{l}^{B,\text{P}}(N_{l}^{B,\text{P}}-\beta )T_{l}\Sigma^{B}T_{l}^{†} \end{array}$$ (27)

Similarly, for the ***x***^*A*^ component of the likelihood, we get a mean of

$$\begin{array}{c} \text{E}\left[x_{l}^{A,\text{P}}\right]=N_{l}^{A,\text{P}}T_{l}S^{G}(S^{T}(\mu^{B})) \end{array}$$ (28)

and variance

$$\begin{array}{l} \text{var}\left(x_{l}^{A,\text{P}}\right)=\alpha N_{l}^{A,\text{P}}M\left(T_{l}S^{G}\left(S^{T}\left(\mu^{B}\right)\right)\right)+N_{l}^{A,\text{P}}\left(N_{l}^{A,\text{P}}-\alpha \right)\times \\ T_{l}\left(DS^{G}{|}_{S^{T}\left(\mu^{B}\right)}\right)\left(\gamma M\left(S^{T}\left(\mu^{B}\right)\right)+\delta \left(DS^{T}{|}_{\mu^{B}}\right)\Sigma^{B}{\left(DS^{T}{|}_{\mu^{B}}\right)}^{†}\right){\left(DS^{G}{|}_{S^{T}\left(\mu^{B}\right)}\right)}^{†}T_{l}^{†} \end{array}$$ (29)

### Data from multiple genes

The mathematical framework outlined above utilises the haplotype information inherent to the data, and accounts for the effect of noise in the sequencing process (Fig 1B and 1C). However, in order to discriminate between changes in viral diversity arising from bottlenecking and selection (Fig 1A) it is necessary to consider data from different regions of the genome at which genetic diversity is nominally statistially independent. At high doses of influenza virus reassortment occurs rapidly, as has been observed both *in vitro* and in small animal infections [82, 83]. In our analysis, distinct viral segments were therefore considered to be independent of one another in this manner, albeit sharing a common transmission bottleneck *N*^*T*^, each transmitted virus being assumed to contain one of each viral segment. As such the likelihood in Eq 23 becomes

$$\begin{array}{c} \log L\left(N^{T},S^{T}|x^{B},x^{A},N^{G},S^{G}\right)=\sum_{m}\sum_{l}\log L\left(N^{T},S_{m}^{T}|x_{ml}^{B,\text{PH}},x_{ml}^{A,\text{PH}},N^{G},S_{m}^{G}\right) \end{array}$$ (30)

where the subscript *m* denotes information particular to a specific genomic region.

### Data from multiple replicates

Replicate data are highly valuable for evolutionary inference [84, 85]. Within our calculation they provide an additional level of abstraction to the inference process. Under this framework we assumed that replicates share a common fitness landscape, *S*^*T*^, whilst exhibiting individual bottleneck values. As a result, the likelihood from Eq 30 becomes

$$\begin{array}{c} \log L\left(N^{T},S^{T}|x^{B},x^{A},N^{G},S^{G}\right)=\sum_{r}\sum_{m}\sum_{l}\log L\left(N_{r}^{T},S_{m}^{T}|x_{rml}^{B,\text{PH}},x_{rml}^{A,\text{PH}},N_{r}^{G},S_{m}^{G}\right) \end{array}$$ (31)

where the subscript *r* denotes information particular to a specific replicate.

### Implementation of Leonard et al. method

For comparison of bottleneck estimates with existing methods we implemented the exact version of the beta-binomial inference scheme of Leonard et al. [12]. The likelihood function for site *i* was defined as

$$\begin{array}{c} L{\left(N^{T}\right)}_{i}=\sum_{j=0}^{N^{T}}P_{\text{beta-bin}}(x_{i,\text{minor}}^{A,\text{SL}}|N_{i}^{A,\text{SL}},j,N^{T}-j)\;P_{\text{bin}}(j|N^{T},q_{i,\text{minor}}^{B,\text{SL}}) \end{array}$$ (32)

where *N*^*T*^ is the bottleneck size, *P*_beta-bin_ is the beta-binomial probability mass function, $x_{i,\text{minor}}^{A,\text{SL}}$ is the number of recipient observations for the minor allele at site *i*, $N_{i}^{A,\text{SL}}$ is the total number of recipient observations for site *i*, i.e. $N_{i}^{A,\text{SL}}=x_{i,\text{minor}}^{A,\text{SL}}+x_{i,\text{major}}^{A,\text{SL}}$, *P*_bin_ is the binomial probability mass function, and $q_{i,\text{minor}}^{B,\text{SL}}$ is the donor frequency for the minor allele at site *i*. We note that the beta-binomial is undefined for *j* = 0 and *j* = *N*^*T*^ and define *j* = 10^−10^ and *j* = *N*^*T*^−10^−10^ respectively in these cases. The original authors did not discuss this further. We did not make use of the cumulative version of the likelihood function as we avoided the problem of variant calling by fixing the number of required polymorphic loci when simulating data. The total likelihood for each bottleneck value was computed as

$$\begin{array}{c} L\left(N^{T}\right)=\sum_{i=0}^{n_{\text{sites}}}L{\left(N^{T}\right)}_{i} \end{array}$$ (33)

where *n*_sites_ is the number of variant loci. Bottleneck inference was defined as the bottleneck associated with the largest likelihood value.

### Application to data

Our method was applied to both simulated sequence data, and data from an evolutionary experiment conducted in ferrets [25].

#### Generation of simulated data

Simulated data were generated in order to nominally reflect data from an influenza transmission event. As such, a single transmission event was modelled as the transmission of viruses each with eight independent segments, the lengths of each segment being equal to the eight segments of the A/H1N1 influenza virus, with five randomly located polymorphic loci in each segment creating a total of 2^5^ potential full haplotypes. One fourth of these haplotypes were randomly chosen under the constraint that each of the five loci had to be polymorphic. Subsequently, full haplotype frequencies were generated at random, with the constraint of a minimum haplotype frequency of 5%.

Transmission was modelled as a multinomial draw of depth equal to the bottleneck size. Selection for transmission was incorporated as a shift in haplotype frequencies as described in Eq 8. Where included in the simulation, selection was assumed to act upon a single variant in one of the viral segments. Within-host growth was modelled as a single round of replication defined as a multinomial draw conferring a 22-fold increase in population size. Within-host selection was incorporated in a manner similar to that of selection for transmission.

Partial haplotype observations were generated on the basis of short-read data simulated for each gene. Short-reads were modelled as randomly placed gapped reads with mean read and gap lengths derived from an example influenza dataset [24] (mean read length = 119.68, SD read length = 136.88, mean gap length = 61.96, SD gap length = 104.48, total read depth = 102825); these estimates are conservative relative to what can be achieved with the best contemporary sequencing technologies. Read depths were calculated for all possible sets of partial haplotypes by assigning individual reads to sets according to the loci they cover. Finally, partial haplotype observations were modelled as Dirichlet-multinomial draws employing a dispersion parameter *C* to account for noise.

Replicate experiments were generated by considering replicate observations of transmission events with consistent viral populations; that is, for which the variant alleles were consistent between replicate transmission events.

#### Experimental sequence data

Data were analysed from an evolutionary experiment in the transmission of a 1918-like influenza virus between ferrets [25]. The specific data examined here describes two sets of viral transmissions. In the first, denoted HA190D220D, a viral population was given to three ferrets, transmission to a recipient host being observed in one of three cases, giving time-resolved sequence data from four ferrets. In the second, denoted Mut, a viral population arising from the first experiment was given to three ferrets, transmission to two recipient hosts being observed, giving data from five ferrets.

#### Processing of sequence data

Genome sequence data was processed using the SAMFIRE software package, according to default settings [57], calling variant alleles that existed at a frequency of at least 1% at some point during the observed infections. For the calculation of a within-host fitness landscape, the effective depth of sequencing was estimated in a conservative manner, filtering out variants which changed in frequency by more than 5% per day before using the frequencies of remaining variants from different time-points within the same host to estimate the parameter *C*. For the within-host model, following the approach of previous calculations [52, 72], potentially non-neutral variants were identified as those for which a model of frequency change under selection outperformed a neutral model by more than 10 units according to the Bayesian Information Criterion (BIC) [58]. Variants reaching a frequency of at least 5% in at least one sample were then identified before calling multi-locus variant observations from the data; data from all time-points for which within-host data were collected were used in this inference. The 5% cutoff was chosen to reduce computational costs for this part of the calculation while still reconstructing the core aspects of the within-host fitness landscape.

For the inference of transmission, data from all polymorphic sites was utilised, with no filtering of sites. As in the original analysis of the data [25], variants were identified from data collected from the final observation before transmission and the first point of observation after transmission; these data were used to construct multi-locus observations across variants which reached a frequency of at least 2% in at least one sample. In this inference a revised approach to estimating the effective depth of sequencing was taken, noting our result that estimates which overestimate noise may lead to errors in the inferred bottleneck size. Here, in common with previous calculations, we initially identified a conservative value of *C* from within-host data using the default settings in SAMFIRE. Next, variant frequencies were evaluated, identifying potentially non-neutral changes in frequency using a single-locus analysis [52]. Finally, a more conservative estimate of *C* was calculated, using the set of trajectories which were identified as being consistent with a neutral model of frequency change.

Subsequent processing was identical for simulated and experimental datasets. Multi-locus variants, detailing partial haplotypes, were identified using SAMFIRE. These were removed from consideration if A) the partial haplotype did not have at least 10 observations either before or after transmission, B) the partial haplotype exhibited a frequency of < 1% before transmission, C) the partial haplotype had no observations before transmission (variant assumed to have arisen de novo), D) the partial haplotype was the only partial haplotype in its set and had no observations post-transmission. Additionally, to avoid potential dataset errors from drastically influencing the inference outcome, partial haplotypes were removed if found to have a single post-transmission observation despite the presence of a large (≥ 50) overall sampling depth. Finally, removal of partial haplotype observations may result in individual loci becoming monomorphic (all partial haplotypes covering these loci exhibit the same alleles). In this case, relevant partial haplotype sets were removed with the reads being redistributed unto variant sets with fewer loci.

SAMFIRE was used to construct a set of haplotypes spanning each viral segment using the multi-locus variant calls from all time points. Here, potential haplotypes are identified by a process of exclusion. Given *n* biallelic variants in a segment, there are 2^n^ potential haplotypes, or combinations of those variants across all loci. SAMFIRE uses observed partial haplotype reads to constrain this set. For example, if across four loci only three of a potential sixteen combinations of alleles are observed, this removes a large proportion of the potential haplotype set. The haplotypes identified in this manner comprise the space of haplotypes spanned by the vectors ***q***^*B*^ and ***q***^*A*^. No inference of haplotype frequencies is conducted at this point, such inference is conducted in a subsequent step, using the likelihood framework described above.

### Inference of parameters

#### Hierarchical selection model

In our model, the set of potential fitness parameters is large. To simplify the calculation, parameters modelling three- or higher-locus epistatic effects were neglected, while parameters modelling two-locus epistasis were only considered for addition to a model which already contained single-locus fitness parameters for each of the two loci. In both the inferences of within-host selection and of transmissibility, a null assumption of neutrality was used as the starting point for an inference model, exploring successively more complex models of selection until an optimal model, defined according to a model selection process, was identified.

#### Inference of within-host selection

For the experimental dataset an inference of within-host selection was conducted according to a method previously described in earlier publications [52, 72]. Under the assumption of rapid reassortment in the system [82] different segments of the virus were treated independently. Our inference of selection aimed to characterise fitness so as to estimate *S*^*G*^ for an inference of transmission; the HA190D225D and Mut datasets were considered independently, with data from all animals in each set being combined to infer within-host selection.

#### Replicate calculations of transmission parameters

Both our within-host and transmission calculations are performed in a model space of potential haplotypes. For example, in the first step of the transmission model, we calculate an estimate for the population ***q***^*B*^ given the data ***x***^*B*^. In many cases, particularly where there are greater numbers of potential haplotypes and short reads span smaller numbers of loci, it is possible that the data ***x***^*B*^ will not uniquely specify the initial vector ***q***^*B*^. Here we are concerned about inferring parameters of transmission, rather than the explicit haplotype reconstruction. Therefore, to check the robustness of our inference, statistical replicate calculations were run, using different reconstructions of ***q***^*B*^ in each case; median inferred parameters across replicates are presented above. To improve the speed of the inference, haplotypes in ***q***^*B*^ with inferred frequencies of less than 10^−10^ were removed from the calculation; subsequent to this, haplotypes were removed in increasing size of inferred frequency until no more than 100 haplotypes remained in ***q***^*B*^ at non-zero frequencies. Results from all statistical replicates are reported in the analysis of the real data.

We note that our inference of ***q***^*B*^ depends upon the initial identification of a plausible set of underlying viral haplotypes using SAMFIRE. A broad set of haplotypes is required for the comparison of different hypotheses about selection in the system. However, where the initial set of haplotypes is very large, as might occur where very short reads describe a great number of polymorphic loci, our approach becomes computationally challenging.

#### Model selection

Model selection was performed using the Bayesian Information Criterion:

$$\begin{array}{c} \text{BIC}=-2\log L+K\log n \end{array}$$ (34)

where *L* is the maximum likelihood obtained for a model, *K* is the number of parameters in the fitness model, and *n* is the number of data points. A range of potential fitness models were explored, the optimal model being identified as that for which the addition of any single fitness parameter failed to bring a significant improvement in BIC.

#### Adaptive BIC

Noting previous discussion of the complexity of using BIC in biological modelling [86], we here adopted a machine-learning approach to the interpretation of BIC statistics. Classically, a difference of 10 units of BIC has been held to represent strong evidence in favour of the additional parameter [58]. Consistent with previous approaches this heuristic was used in the inference of within-host selection; in this case the final model parameters make only small refinements to the inferred fitness landscape [52]. In the inference of transmission, errors in model selection have more severe consequences for the inferred bottleneck size and selection model. Using a fixed difference of 10 BIC units for model selection resulted in an overestimation of the extent of selection with a high false positive rate (S14 Fig). As such, we generated and analysed simulated data to identify the optimal interpretation of BIC differences. Given a real dataset for analysis, simulated data was generated describing systems with equivalent numbers of gene segments and polymorphic loci to the real dataset, being observed with an equal number of reads spanning each set of loci, and with reads containing an amount of information specified by the parameter *C* inferred for the real dataset.

Next, inferences were conducted on data describing neutral transmission events with bottlenecks in the range [5, 100]. As shown in Fig 3, the ability to infer a correct neutral bottleneck is impaired by noise for transmission events involving a large number of viruses; linear regression was used to obtain a simple function describing the ratio between the median inferred and true bottleneck sizes under neutrality (S15A Fig); this parameterises our expectation of the ‘correct’ inferred bottleneck size for any given real bottleneck, once noise is accounted for.

Secondly, using this baseline to set our expectations, a parameterisation was carried out to find a BIC penalty function that gave the largest accuracy in bottleneck inference. To this end, three datasets were considered; a neutral dataset and two datasets with single selection coefficients of *s* = {1, 2} respectively. BIC penalty values in the range [10, 200] were examined, with smaller BIC penalty values leading to inferences with a larger number of selection coefficients and vice versa. For each BIC penalty value, the difference between the bottleneck inference of the optimal model (under BIC) and the baseline expectation was summed for the three datasets to give a statistic describing the accuracy of the inferred bottlenecks, this statistic being expressed as a function of the real transmission bottleneck *N*^*T*^ and the BIC penalty (S15B Fig). Finally, linear and decay exponential models were fitted to this data via regression, selecting the BIC penalty model which minimised the error in the inferred bottlenecks from the simulation data. We note that our penalty is a function of the inferred population bottleneck, higher penalties being inferred for tight bottlenecks and lower penalties being inferred for looser bottlenecks.

Thirdly, the inferred data were reinterpreted to derive a BIC penalty optimal for the inference of selection. We note that, even with a BIC penalty function optimised for bottleneck inference, there may still remain cases where, through the stochastic process of transmission, the genetic composition of the population changes in a manner consistent with the action of selection, granting a false positive inference. A second BIC penalty was learned as above, this time maximising the accuracy of the inference or non-inference of selection parameters, defined as

$$\frac{\#\text{true}\;\text{positives}+\#\text{true}\;\text{negatives}}{\#\text{true}\;\text{positives}+\#\text{false}\;\text{positives}+\#\text{true}\;\text{negatives}+\#\text{false}\;\text{negatives}}$$ (35)

This conservative BIC penalty function typically led to an underestimate for the inferred bottleneck; the two BIC penalty functions were used in concert to estimate *N*^*T*^ and *S*^*T*^ in separate calculations. The BIC penalty functions are specific to individual datasets and, as a result, recalculation of BIC penalty functions is required when considering new data. Inference of BIC penalty functions is only necessary when attempting to jointly determine bottleneck and selection; for inference of transmission bottlenecks only, our method is remarkably simple and fast.

As noted elsewhere, where a genomic variant fixes between two observations, this change in frequency can be explained by the fitting of an arbitrarily large selection coefficient; no upper bound on selection can be established [87]. Within our framework, if this is not accounted for, extremely strong selection may be falsely inferred to explain the loss of variants during a transmission bottleneck. To guard against this, models of transmission in which the inferred magnitude of selection was outside of the range (-10, 10) were excluded from consideration. In the within-host analysis methods, haplotype fitness are not constrained; here, to avoid errors of machine precision, the magnitudes of extreme fitness inferences were reduced to be within the range (-10, 10). For the same reason, elements of the mean and covariance matrix of ***q***^*B*^ were constrained to be greater in magnitude than 10^−11^. While selection coefficients outside of this range have been identified [88], these steps greatly reduce the number of false inferences of strong selection.

### Online repository and instructions for use

Code and scripts related to this project can be found online at https://bitbucket.org/casperlu/transmission_project/. Detailed descriptions of code options and user guides are available in the repository README files. Scripts and instructions relevant for generating the figures in this paper may be found online as well.

The overall workflow is as follows: Time series viral sequence data are prepared in SAM format. SAMFIRE is used for filtering and splitting of data unto relevant time points, e.g. those surrounding transmission. Single- and multi-locus trajectories are computed using SAMFIRE. A list of potential haplotypes (see [52] for details) are constructed. A noise parameter *C* and potential within-host selection is inferred using SAMFIRE. The transmission code is used for generating simulated data based on the experimental data. Transmission bottleneck and selection is inferred for the simulated data and BIC penalty curves are determined. A final inference of bottleneck and transmissibility is carried out on the real data taking into account the BIC penalty curves. This process will be greatly simplified in the case where only a neutral estimate of the transmission bottleneck is required. Quick-start and step-by-step guides can be found in the online transmission repository.

## Supporting information

S1 Fig Effect of the noise parameter *C* on a one-dimensional distribution. Allele frequency distribution for a sample of read depth *N* = 1000 collected from a population with true allele frequency one third, with a noise-free sampling method (*C* = ∞) and with C values of 10, 100, and 1000.
(PDF)

S2 Fig Effect of incorrect estimation of the noise parameter *C*. Bottleneck inference under a neutral model applied to neutral data with simulation dispersion parameters of *C* = {50, 10^6^}. Inference was performed using a range of dispersion parameters, *C* = {50, 100, 200, 500, 1000, 10^6^}. Each datapoint represents a median over 200 simulation seeds.
(PDF)

S3 Fig Median inferred bottleneck size from data simulating neutral transmission and transmission with a single locus under selection, from three replicate systems. Inferences were made using either a neutral model, in which the effect of selection was assumed to be zero, or a selection model, which allowed scenarios involving selection to be identified. Median inferences are shown from 200 simulations, each involving three replicate transmission events, for each datapoint.
(PDF)

S4 Fig Inferred bottleneck sizes *N*^*T*^ for a range of true bottleneck sizes, applying a neutral inference model to simulated transmission data with selection. Results were generated by applying a neutral inference model to selected simulated data. Results are shown for 200 simulations at each bottleneck size.
(PDF)

S5 Fig Inferred bottleneck sizes *N*^*T*^ for a range of true bottleneck sizes, applying an inference model accounting for selection to simulated transmission data with selection. Results were generated by applying an inference model accounting for selection to selected simulated data. Results are shown for 200 simulations at each bottleneck size.
(PDF)

S6 Fig Inferred bottleneck sizes *N*^*T*^ for a range of true bottleneck sizes, applying a neutral inference model to simulated transmission data with selection. Data were collected from three replicate transmission events. Results were generated by applying a neutral inference model to selected simulated data. Results are shown for 200 simulations at each bottleneck size, each simulation describing three replicate transmission events.
(PDF)

S7 Fig Inferred bottleneck sizes *N*^*T*^ for a range of true bottleneck sizes, applying an inference model accounting for selection to simulated transmission data with selection. Data were collected from three replicate transmission events. Results were generated by applying a neutral inference model to selected simulated data. Results are shown for 200 simulations at each bottleneck size, each simulation describing three replicate transmission events.
(PDF)

S8 Fig Probability distributions of inferred selection coefficients from 200 simulations each of three transmission events. Distributions were constructed for bottleneck values where the inference of selection resulted in a true positive rate for identifying selected variants of above 5%. Smooth kernel distributions were computed as for Fig 7.
(PDF)

S9 Fig Inferred within-host fitness landscape for segments in the HA190D220D viral populations. Haplotypes for which the inferred frequency rose to a frequency of at least 1% in at least one animal are shown. Haplotypes which are separated by a single mutation are joined by lines.
(PDF)

S10 Fig Inferred within-host fitness landscape for segments in the Mut viral populations. Haplotypes for which the inferred frequency rose to a frequency of at least 1% in at least one animal are shown. Haplotypes which are separated by a single mutation are joined by lines.
(PDF)

S11 Fig Histograms of selection inferences for the Mut transmission pairs from 200 seeds using an allele frequency cut-off of 2%. A replicate inference method was employed such that a common fitness landscape was imposed. Selection inferences that resulted in at least 10% non-zero inferences are here reported by the nucleotide position of the variant site.
(PDF)

S12 Fig Histograms of bottleneck inferences for HA190D225D and Mut transmission pairs from 200 random seeds using allele frequency cut-offs of 3% and 4%. A replicate inference method was employed for the Mut transmission pairs such that a common fitness landscape was imposed. The Mut transmission pairs may take different bottleneck values and have been plotted as an overlapping histogram. Bottleneck inferences larger than *N*^*T*^ = 35 have been omitted for clarity.
(PDF)

S13 Fig Median inferred bottleneck size from simulated neutral transmission data under a modified inference method. Inferences were made using either the standard neutral model, in which the covariance matrix ***q***^*B*^ is diagonal, or using a simplified model ignoring the variance in ***q***^*B*^. Each datapoint represents a median over 200 simulation seeds.
(PDF)

S14 Fig True and false positive rates of selection inference given a standard interpretation of BIC. A fixed BIC difference of 10 units were employed in the model selection process, requiring a model with a single additional parameter to generate an improvement of at least 10 units to BIC to be accepted. While such a difference is accepted as showing strong evidence in favour of the more complex model, in our case it generated a high rate of false positive inferences of selection.
(PDF)

S15 Fig Determining BIC penalty function for bottleneck inference under simulated data. A) The ratio of the median inferred bottleneck to the true bottleneck is plotted against the true bottleneck size. As shown in Fig 3, as the bottleneck increases, our ability to infer it correctly decreases due to noise. In order to account for this phenomenon, a straight line is fitted to the data aiming to capture the general trend. B) Heat map of the bottleneck-specific statistic plotted against BIC penalty and bottleneck size. The plot was generated for three datasets with selection coefficients *s* = {0, 1, 2} and a simple statistic based on bottleneck differences was employed. More specifically, the median bottleneck was computed across 200 seeds and the bottleneck-statistic was defined as the absolute value of the difference between the median inferred bottleneck and the true bottleneck multiplied by the baseline determined in A). By considering bottlenecks in the range [5, 100] and BIC penalty values in the range [10, 200], a heat map was produced and linear and decay exponential regression were conducted seeking to minimise the sum of the statistic across the values of *N*^*T*^ that were considered.
(PDF)

S1 Text Derivation of compound distributions for a multi-step within-host growth process. We consider both the neutral case and that where selection applies within-host.
(PDF)

S2 Text Mathematical notes on a useful identity. (PDF)

S1 Table Inferred fitness coefficients for the within-host evolution of the virus within each experiment. Parameters were inferred across all index and contact ferrets within each experiment and are reported to a single decimal place. Only polymorphisms at which within-host selection was identified are listed. The parameter *χ* denotes an epistatic interaction between variant alleles. We note that our method infers the approximate shape of a fitness landscape based upon a reconstruction of whole viral segments; individual selection coefficients may be subject to variance between similar fitness landscapes.
(PDF)

S1 Data Values corresponding to figures. Numerical values corresponding to figures in the main text are shown.
(XLSX)

## References

[1] Fraser C, Donnelly CA, Cauchemez S, Hanage WP, Van Kerkhove MD, Hollingsworth TD, et al Pandemic Potential of a Strain of Influenza A (H1N1): Early Findings. Science. 2009;324(5934):1557–1561. 10.1126/science.1176062 19433588 PMC3735127

[2] Breban R, Riou J, Fontanet A. Interhuman transmissibility of Middle East respiratory syndrome coronavirus: estimation of pandemic risk. The Lancet. 2013;382(9893):694–699. 10.1016/S0140-6736(13)61492-0 PMC7159280 23831141

[3] Bergstrom CT, McElhany P, Real LA. Transmission bottlenecks as determinants of virulence in rapidly evolving pathogens. Proceedings of the National Academy of Sciences of the United States of America. 1999;96(9):5095–5100. 10.1073/pnas.96.9.5095 10220424 PMC21822

[4] Gutiérrez S, Michalakis Y, Blanc S. Virus population bottlenecks during within-host progression and host-to-host transmission. Current Opinion in Virology. 2012;2(5):546–555. 10.1016/j.coviro.2012.08.001 22921636

[5] Varble A, Albrecht RA, Backes S, Crumiller M, Bouvier NM, Sachs D, et al Influenza A Virus Transmission Bottlenecks Are Defined by Infection Route and Recipient Host. Cell Host and Microbe. 2014;16(5):691–700. 10.1016/j.chom.2014.09.020 25456074 PMC4272616

[6] Frise R, Bradley K, van Doremalen N, Galiano M, Elderfield RA, Stilwell P, et al Contact transmission of influenza virus between ferrets imposes a looser bottleneck than respiratory droplet transmission allowing propagation of antiviral resistance. Scientific Reports. 2016;6(1):29793 10.1038/srep29793 27430528 PMC4949428

[7] Sacristan S, Malpica JM, Fraile A, Garcia-Arenal F. Estimation of Population Bottlenecks during Systemic Movement of Tobacco Mosaic Virus in Tobacco Plants. Journal of Virology. 2003;77(18):9906–9911. 10.1128/JVI.77.18.9906-9911.2003 12941900 PMC224588

[8] Krimbas CB, Tsakas S. The Genetics of Dacus Oleae. V. Changes of esterase polymorphism in a natural population following insecticide control—selection or drift? Evolution. 1971;25(3):454–460. 10.1111/j.1558-5646.1971.tb01904.x 28565021

[9] Monsion B, Froissart R, Michalakis Y, Blanc S. Large bottleneck size in Cauliflower Mosaic Virus populations during host plant colonization. PLoS Pathogens. 2008;4(10):e1000174 10.1371/journal.ppat.1000174 18846207 PMC2553192

[10] Charlesworth B. Fundamental concepts in genetics: Effective population size and patterns of molecular evolution and variation. Nature Reviews Genetics. 2009;10(3):195–205. 10.1038/nrg2526 19204717

[11] Khiabanian H, Emmett KJ, Lee A, Rabadan R. High-resolution Genomic Surveillance of 2014 Ebolavirus Using Shared Subclonal Variants. PLoS currents. 2015;7:1–17. 10.1371/currents.outbreaks.c7fd7946ba606c982668a96bcba43c90 PMC4339230 25737802

[12] Sobel Leonard A, Weissman DB, Greenbaum B, Ghedin E, Koelle K. Transmission Bottleneck Size Estimation from Pathogen Deep-Sequencing Data, with an Application to Human Influenza A Virus. Journal of Virology. 2017;91(14):e00171–17–19. 10.1128/JVI.00171-17 28468874 PMC5487570

[13] McCrone JT, Woods RJ, Martin ET, Malosh RE, Monto AS, Lauring AS. Stochastic processes constrain the within and between host evolution of influenza virus. eLife. 2018;7:e35962 10.7554/eLife.35962 29683424 PMC5933925

[14] Poon LLM, Song T, Rosenfeld R, Lin X, Rogers MB, Zhou B, et al Quantifying influenza virus diversity and transmission in humans. Nature Genetics. 2016;48(2):195–200. 10.1038/ng.3479 26727660 PMC4731279

[15] Xue KS, Bloom JD. Reconciling disparate estimates of viral genetic diversity during human influenza infections. bioRxiv. 2018; 10.1038/s41588-019-0349-3 PMC6708745 30804564

[16] Kuiken T, Holmes EC, McCauley J, Rimmelzwaan GF, Williams CS, Grenfell BT. Host species barriers to influenza virus infections. Science. 2006;312(5772):394–397. 10.1126/science.1122818 16627737

[17] Lipsitch M, Barclay W, Raman R, Russell CJ, Belser JA, Cobey S, et al Viral factors in influenza pandemic risk assessment. eLife. 2016;5:316ra192 10.7554/eLife.18491 PMC5156527 27834632

[18] Herfst S, Schrauwen EJA, Linster M, Chutinimitkul S, de Wit E, Munster VJ, et al Airborne Transmission of Influenza A/H5N1 Virus Between Ferrets. Science. 2012;336(6088):1534–1541. 10.1126/science.1213362 22723413 PMC4810786

[19] Imai M, Watanabe T, Hatta M, Das SC, Ozawa M, Shinya K, et al Experimental adaptation of an influenza H5 HA confers respiratory droplet transmission to a reassortant H5 HA/H1N1 virus in ferrets. Nature. 2012;486(7403):420–428. 10.1038/nature10831 22722205 PMC3388103

[20] Sutton TC, Finch C, Shao H, Angel M, Chen H, Capua I, et al Airborne transmission of highly pathogenic H7N1 influenza virus in ferrets. Journal of Virology. 2014;88(12):6623–6635. 10.1128/JVI.02765-13 24696487 PMC4054360

[21] Yang H, Chen Y, Qiao C, He X, Zhou H, Sun Y, et al Prevalence, genetics, and transmissibility in ferrets of Eurasian avian-like H1N1 swine influenza viruses. Proceedings of the National Academy of Sciences. 2016;113(2):392–397. 10.1073/pnas.1522643113 PMC4720320 26711995

[22] Palese P, Wang TT. H5N1 influenza viruses: facts, not fear. Proceedings of the National Academy of Sciences. 2012;109(7):2211–2213. 10.1073/pnas.1121297109 PMC3289294 22308474

[23] Buhnerkempe MG, Gostic K, Park M, Ahsan P, Belser JA, Lloyd-Smith JO. Mapping influenza transmission in the ferret model to transmission in humans. eLife. 2015;4:e29971 10.7554/eLife.07969 PMC4586390 26329460

[24] Wilker PR, Dinis JM, Starrett G, Imai M, Hatta M, Nelson CW, et al Selection on haemagglutinin imposes a bottleneck during mammalian transmission of reassortant H5N1 influenza viruses. Nature Communications. 2013;4:1–11. 10.1038/ncomms3636 PMC3845350 24149915

[25] Moncla LH, Zhong G, Nelson CW, Dinis JM, Mutschler J, Hughes AL, et al Selective Bottlenecks Shape Evolutionary Pathways Taken during Mammalian Adaptation of a 1918-like Avian Influenza Virus. Cell Host and Microbe. 2016;19(2):169–180. 10.1016/j.chom.2016.01.011 26867176 PMC4761429

[26] Acevedo A, Brodsky L, Andino R. Mutational and fitness landscapes of an RNA virus revealed through population sequencing. Nature. 2014;505(7485):686–690. 10.1038/nature12861 24284629 PMC4111796

[27] Visher E, Whitefield SE, McCrone JT, Fitzsimmons W, Lauring AS. The Mutational Robustness of Influenza A Virus. PLoS Pathogens. 2016;12(8):e1005856–25. 10.1371/journal.ppat.1005856 27571422 PMC5003363

[28] Rouzine IM, Rodrigo A, Coffin JM. Transition between stochastic evolution and deterministic evolution in the presence of selection: general theory and application to virology. Microbiology and Molecular Biology Reviews. 2001;65(1):151–185. 10.1128/MMBR.65.1.151-185.2001 11238990 PMC99023

[29] Zwart MP, Daròs JA, Elena SF. One Is Enough: In Vivo Effective Population Size Is Dose-Dependent for a Plant RNA Virus. PLoS Pathogens. 2011;7(7):e1002122–12. 10.1371/journal.ppat.1002122 21750676 PMC3131263

[30] Abel S, Abel zur Wiesch P, Davis BM, Waldor MK. Analysis of Bottlenecks in Experimental Models of Infection. PLoS Pathogens. 2015;11(6):e1004823–7. 10.1371/journal.ppat.1004823 26066486 PMC4465827

[31] O’Hara RB. Comparing the effects of genetic drift and fluctuating selection on genotype frequency changes in the scarlet tiger moth. Proceedings of the Royal Society B: Biological Sciences. 2005;272(1559):211–217. 10.1098/rspb.2004.2929 15695213 PMC1634953

[32] Bollback JP, York TL, Nielsen R. Estimation of 2Nes From Temporal Allele Frequency Data. Genetics. 2008;179(1):497–502. 10.1534/genetics.107.085019 18493066 PMC2390626

[33] Malaspinas AS, Malaspinas O, Evans SN, Slatkin M. Estimating allele age and selection coefficient from time-serial data. Genetics. 2012;192(2):599–607. 10.1534/genetics.112.140939 22851647 PMC3454883

[34] Feder AF, Kryazhimskiy S, Plotkin JB. Identifying signatures of selection in genetic time series. Genetics. 2014;196(2):509–522. 10.1534/genetics.113.158220 24318534 PMC3914623

[35] Foll M, Poh YP, Renzette N, Ferrer-Admetlla A, Bank C, Shim H, et al Influenza Virus Drug Resistance: A Time-Sampled Population Genetics Perspective. PLoS Genetics. 2014;10(2):e1004185 10.1371/journal.pgen.1004185 24586206 PMC3937227

[36] Terhorst J, Schlötterer C, Song YS. Multi-locus Analysis of Genomic Time Series Data from Experimental Evolution. PLoS Genetics. 2015;11(4):e1005069–29. 10.1371/journal.pgen.1005069 25849855 PMC4388667

[37] Chare ER, Gould EA, Holmes EC. Phylogenetic analysis reveals a low rate of homologous recombination in negative-sense RNA viruses. The Journal of general virology. 2003;84(Pt 10):2691–2703. 10.1099/vir.0.19277-0 13679603

[38] Boni MF, Zhou Y, Taubenberger JK, Holmes EC. Homologous recombination is very rare or absent in human influenza A virus. Journal of Virology. 2008;82(10):4807–4811. 10.1128/JVI.02683-07 18353939 PMC2346757

[39] Neher RA, Shraiman BI. Competition between recombination and epistasis can cause a transition from allele to genotype selection. Proceedings of the National Academy of Sciences. 2009;106(16):6866–6871. 10.1073/pnas.0812560106 PMC2672512 19366665

[40] Strelkowa N, Lässig M. Clonal interference in the evolution of influenza. Genetics. 2012;192(2):671–682. 10.1534/genetics.112.143396 22851649 PMC3454888

[41] Illingworth CJR, Mustonen V. Components of Selection in the Evolution of the Influenza Virus: Linkage Effects Beat Inherent Selection. PLoS Pathogens. 2012;8(12):e1003091 10.1371/journal.ppat.1003091 23300444 PMC3531508

[42] Koelle K, Rasmussen DA. The effects of a deleterious mutation load on patterns of influenza A/H3N2’s antigenic evolution in humans. eLife. 2015;4:e07361 10.7554/eLife.07361 26371556 PMC4611170

[43] Felsenstein J. Inbreeding and variance effective numbers in populations with overlapping generations. Genetics. 1971;68(4):581–597. 5166069 10.1093/genetics/68.4.581 PMC1212678

[44] Wang C, Mitsuya Y, Gharizadeh B, Ronaghi M, Shafer RW. Characterization of mutation spectra with ultra-deep pyrosequencing: Application to HIV-1 drug resistance. Genome Research. 2007;17(8):1195–1201. 10.1101/gr.6468307 17600086 PMC1933516

[45] Varghese V, Wang E, Babrzadeh F, Bachmann MH, Shahriar R, Liu T, et al Nucleic Acid Template and the Risk of a PCR-Induced HIV-1 Drug Resistance Mutation. PLoS ONE. 2010;5(6):e10992–6. 10.1371/journal.pone.0010992 20539818 PMC2881873

[46] Beerenwinkel N, Zagordi O. Ultra-deep sequencing for the analysis of viral populations. Current Opinion in Virology. 2011;1(5):413–418. 10.1016/j.coviro.2011.07.008 22440844

[47] van Dijk EL, Auger H, Jaszczyszyn Y, Thermes C. Ten years of next-generation sequencing technology. Trends in Genetics. 2014;30(9):418–426. 10.1016/j.tig.2014.07.001 25108476

[48] Iyer S, Casey E, Bouzek H, Kim M, Deng W, Larsen BB, et al Comparison of Major and Minor Viral SNPs Identified through Single Template Sequencing and Pyrosequencing in Acute HIV-1 Infection. PLoS ONE. 2015;10(8):e0135903 10.1371/journal.pone.0135903 26317928 PMC4552882

[49] Laehnemann D, Borkhardt A, McHardy AC. Denoising DNA deep sequencing data-high-throughput sequencing errors and their correction. Briefings in Bioinformatics. 2016;17(1):154–179. 10.1093/bib/bbv029 26026159 PMC4719071

[50] McCrone JT, Lauring AS. Measurements of Intrahost Viral Diversity Are Extremely Sensitive to Systematic Errors in Variant Calling. Journal of Virology. 2016;90(15):6884–6895. 10.1128/JVI.00667-16 27194763 PMC4944299

[51] Sandmann S, de Graaf AO, Karimi M, van der Reijden BA, Hellström-Lindberg E, Jansen JH, et al Evaluating Variant Calling Tools for Non-Matched Next-Generation Sequencing Data. Scientific Reports. 2017;7:43169 10.1038/srep43169 28233799 PMC5324109

[52] Illingworth CJR. Fitness Inference from Short-Read Data: Within-Host Evolution of a Reassortant H5N1 Influenza Virus. Molecular Biology and Evolution. 2015;32(11):3012–3026. 10.1093/molbev/msv171 26243288 PMC4651230

[53] Zanini F, Brodin J, Albert J, Neher RA. Error rates, PCR recombination, and sampling depth in HIV-1 whole genome deep sequencing. Virus Research. 2017;239:106–114. 10.1016/j.virusres.2016.12.009 28039047

[54] Illingworth CJR, Roy S, Beale MA, Tutill H, Williams R, Breuer J. On the effective depth of viral sequence data. Virus Evolution. 2017;3(2):1–9. 10.1093/ve/vex030 PMC5724399 29250429

[55] Blanquart F, Grabowski MK, Herbeck J, Nalugoda F, Serwadda D, Eller MA, et al A transmission-virulence evolutionary trade-off explains attenuation of HIV-1 in Uganda. eLife. 2016;5:2171 10.7554/eLife.20492 PMC5115872 27815945

[56] Zaraket H, Baranovich T, Kaplan BS, Carter R, Song MS, Paulson JC, et al Mammalian adaptation of influenza A(H7N9) virus is limited by a narrow genetic bottleneck. Nature Communications. 2015;6:1–10. 10.1038/ncomms7553 PMC4403340 25850788

[57] Illingworth CJR. SAMFIRE: multi-locus variant calling for time-resolved sequence data. Bioinformatics. 2016;32(14):2208–2209. 10.1093/bioinformatics/btw205 27153641 PMC4937198

[58] Kass RE, Raftery AE. Bayes factors. Journal of the American Statistical Association. 1995;90(430):773–795. 10.1080/01621459.1995.10476572

[59] Silverman BW. Density estimation for statistics and data analysis. Chapman and Hall; 1986.

[60] Baccam P, Beauchemin C, Macken CA, Hayden FG, Perelson AS. Kinetics of influenza A virus infection in humans. Journal of virology. 2006;80(15):7590–9. 10.1128/JVI.01623-05 16840338 PMC1563736

[61] Watanabe T, Zhong G, Russell CA, Nakajima N, Hatta M, Hanson A, et al Circulating avian influenza viruses closely related to the 1918 virus have pandemic potential. Cell host & microbe. 2014;15(6):692–705. 10.1016/j.chom.2014.05.006 24922572 PMC4205238

[62] Zwart MP, Elena SF. Matters of Size: Genetic Bottlenecks in Virus Infection and Their Potential Impact on Evolution. Annual Review of Virology. 2015;2(1):161–179. 10.1146/annurev-virology-100114-055135 26958911

[63] Derdeyn CA, Decker JM, Bibollet-Ruche F, Mokili JL, Muldoon M, Denham SA, et al Envelope-Constrained Neutralization-Sensitive HIV-1 after Heterosexual Transmission. Science. 2004;303(5666):2019–2022. 10.1126/science.1093137 15044802

[64] Edwards CTT, Holmes EC, Wilson DJ, Viscidi RP, Abrams EJ, Phillips RE, et al Population genetic estimation of the loss of genetic diversity during horizontal transmission of HIV-1. BMC Evolutionary Biology. 2006;6:28 10.1186/1471-2148-6-28 16556318 PMC1444934

[65] Li LM, Grassly NC, Fraser C. Quantifying Transmission Heterogeneity Using Both Pathogen Phylogenies and Incidence Time Series. Molecular Biology and Evolution. 2017;34(11):2982–2995. 10.1093/molbev/msx195 28981709 PMC5850343

[66] Yen HL, Liang CH, Wu CY, Forrest HL, Ferguson A, Choy KT, et al Hemagglutinin–neuraminidase balance confers respiratory-droplet transmissibility of the pandemic H1N1 influenza virus in ferrets. Proceedings of the National Academy of Sciences of the United States of America. 2011;108(34):14264–14269. 10.1073/pnas.1111000108 21825167 PMC3161546

[67] Steel J, Lowen AC, Mubareka S, Palese P. Transmission of influenza virus in a mammalian host is increased by PB2 amino acids 627K or 627E/701N. PLoS Pathogens. 2009;5(1):e1000252 10.1371/journal.ppat.1000252 19119420 PMC2603332

[68] Linster M, van Boheemen S, de Graaf M, Schrauwen EJA, Lexmond P, Mänz B, et al Identification, Characterization, and Natural Selection of Mutations Driving Airborne Transmission of A/H5N1 Virus. Cell. 2014;157(2):329–339. 10.1016/j.cell.2014.02.040 24725402 PMC4003409

[69] Nishiura H, Yen HL, Cowling BJ. Sample Size Considerations for One-to-One Animal Transmission Studies of the Influenza A Viruses. PLoS ONE. 2013;8(1):e55358–7. 10.1371/journal.pone.0055358 23383167 PMC3561278

[70] Dinis JM, Florek NW, Fatola OO, Moncla LH, Mutschler JP, Charlier OK, et al Deep Sequencing Reveals Potential Antigenic Variants at Low Frequencies in Influenza A Virus-Infected Humans. Journal of Virology. 2016;90(7):3355–3365. 10.1128/JVI.03248-15 26739054 PMC4794676

[71] Zanini F, Brodin J, Thebo L, Lanz C, Bratt G, Albert J, et al Population genomics of intrapatient HIV-1 evolution. eLife. 2015;4 10.7554/eLife.11282 26652000 PMC4718817

[72] Sobel Leonard A, McClain MT, Smith GJD, Wentworth DE, Halpin RA, Lin X, et al The effective rate of influenza reassortment is limited during human infection. PLoS Pathogens. 2017;13(2):e1006203–26. 10.1371/journal.ppat.1006203 28170438 PMC5315410

[73] Coombs D, Gilchrist MA, Ball CL. Evaluating the importance of within- and between-host selection pressures on the evolution of chronic pathogens. Theoretical Population Biology. 2007;72(4):576–591. 10.1016/j.tpb.2007.08.005 17900643

[74] Illingworth CJR, Fischer A, Mustonen V. Identifying selection in the within-host evolution of influenza using viral sequence data. PLoS Computational Biology. 2014;10(7):e1003755 10.1371/journal.pcbi.1003755 25080215 PMC4117419

[75] Kimura M. Stochastic processes and distribution of gene frequencies under natural selection. Cold Spring Harbor symposia on quantitative biology. 1955;20:33–53. 10.1101/SQB.1955.020.01.006 13433553

[76] Stray SJ, Air GM. Apoptosis by influenza viruses correlates with efficiency of viral mRNA synthesis. Virus Research. 2001;77(1):3–17. 10.1016/S0168-1702(01)00260-X 11451482

[77] Lacerda M, Seoighe C. Population Genetics Inference for Longitudinally-Sampled Mutants Under Strong Selection. Genetics. 2014;198(3):1237–1250. 10.1534/genetics.114.167957 25213172 PMC4224163

[78] Tran TD, Hofrichter J, Jost J. The evolution of moment generating functions for the Wright-Fisher model of population genetics. Mathematical Biosciences. 2014;256:10–17. 10.1016/j.mbs.2014.07.007 25065291

[79] Tataru P, Simonsen M, Bataillon T, Hobolth A. Statistical Inference in the Wright-Fisher Model Using Allele Frequency Data. Systematic Biology. 2017;66(1):e30–e46. 10.1093/sysbio/syw056 28173553 PMC5837693

[80] Mosimann JE. On the Compound Multinomial Distribution, the Multivariate *β*-Distribution, and Correlations Among Proportions. Biometrika. 1962;49(1/2):65 10.1093/biomet/49.1-2.65

[81] Johnson SG. Multi-dimensional adaptive integration (cubature) in C;. https://github.com/stevengj/cubature.

[82] Marshall N, Priyamvada L, Ende Z, Steel J, Lowen AC. Influenza Virus Reassortment Occurs with High Frequency in the Absence of Segment Mismatch. PLoS Pathogens. 2013;9(6):e1003421 10.1371/journal.ppat.1003421 23785286 PMC3681746

[83] Tao H, Steel J, Lowen AC. Intrahost Dynamics of Influenza Virus Reassortment. Journal of Virology. 2014;88(13):7485–7492. 10.1128/JVI.00715-14 24741099 PMC4054463

[84] Kofler R, Schlötterer C. A guide for the design of evolve and resequencing studies. Molecular Biology and Evolution. 2014;31(2):474–483. 10.1093/molbev/mst221 24214537 PMC3907048

[85] Achaz G, Rodriguez-Verdugo A, Gaut BS, Tenaillon O. The reproducibility of adaptation in the light of experimental evolution with whole genome sequencing. Adv Exp Med Biol. 2014;781:211–31. 10.1007/978-94-007-7347-9_11 24277302

[86] Fischer A, Vázquez-García I, Illingworth CJR, Mustonen V. High-Definition Reconstruction of Clonal Composition in Cancer. CellReports. 2014;7(5):1740–1752. 10.1016/j.celrep.2014.04.055 PMC4062932 24882004

[87] Illingworth CJR, Mustonen V. A method to infer positive selection from marker dynamics in an asexual population. Bioinformatics. 2012;28(6):831–837. 10.1093/bioinformatics/btr722 22223745 PMC3307107

[88] Bull JJ, Badgett MR, Wichman HA. Big-Benefit Mutations in a Bacteriophage Inhibited with Heat. Molecular Biology and Evolution. 2000;17(6):942–950. 10.1093/oxfordjournals.molbev.a026375 10833201

