# Supplementary figures and images for: A novel framework for inferring parameters of transmission from viral sequence data
Source: PLoS Genet. 2018 Oct 16;14(10):e1007718. doi: 10.1371/journal.pgen.1007718 (PMC6203404; doi:10.1371/journal.pgen.1007718)

Read depth  $N=1000$

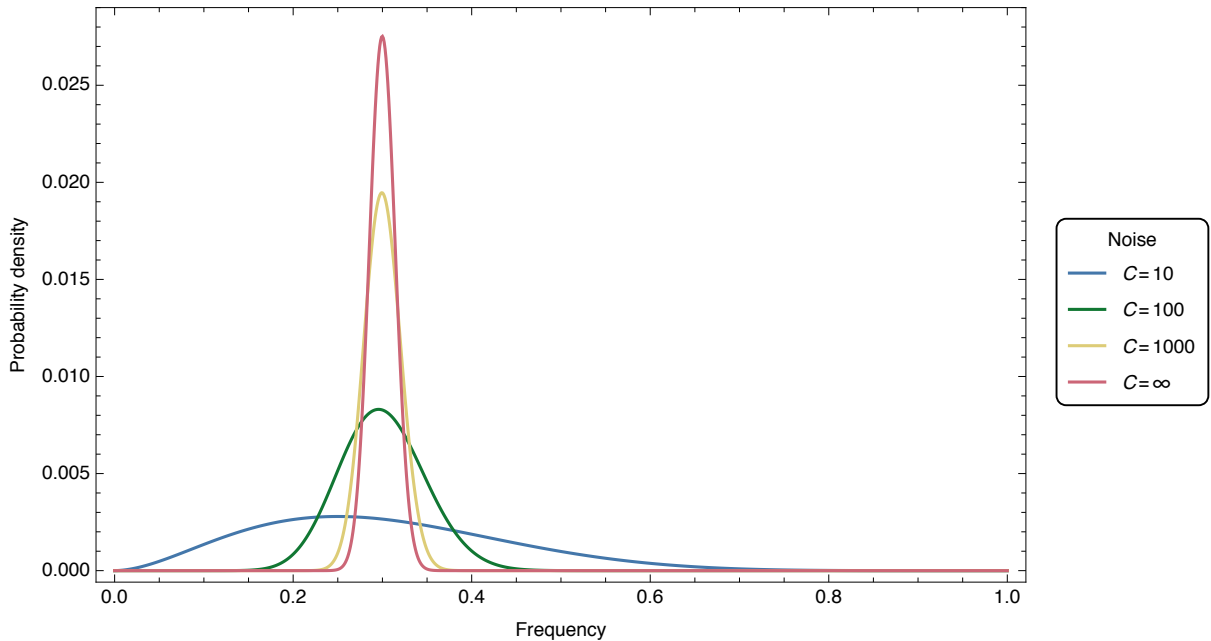

Supplement: S1 Fig — Allele frequency distribution for a sample of read depth N = 1000 collected from a population with true allele frequency one third, with a noise-free sampling method (C = ∞) and with C values of 10, 100, and 1000. (PDF) [file pgen.1007718.s001.pdf]

Simulation  $C=50$ 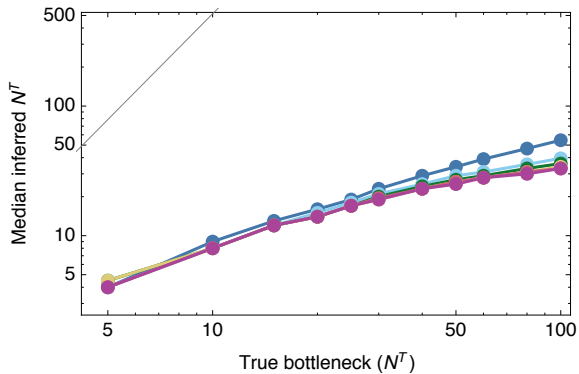Simulation  $C=10^6$ 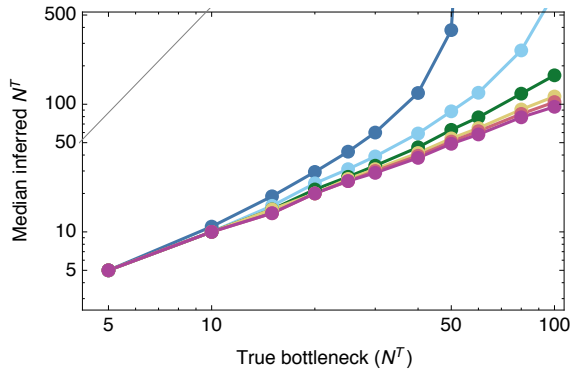

Analysis C

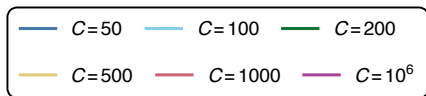

Supplement: S2 Fig — Bottleneck inference under a neutral model applied to neutral data with simulation dispersion parameters of C = {50, 106}. Inference was performed using a range of dispersion parameters, C = {50, 100, 200, 500, 1000, 106}. Each datapoint represents a median over 200 simulation seeds. (PDF) [file pgen.1007718.s002.pdf]

Neutral model

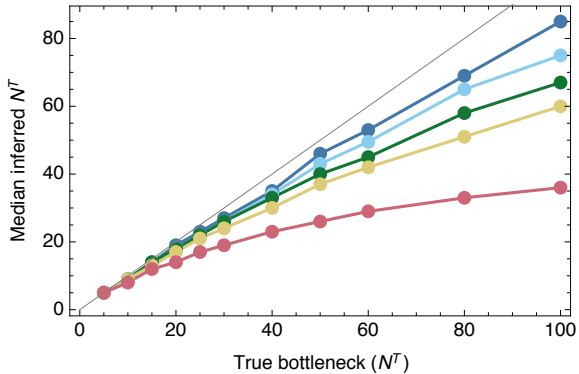

Selection model

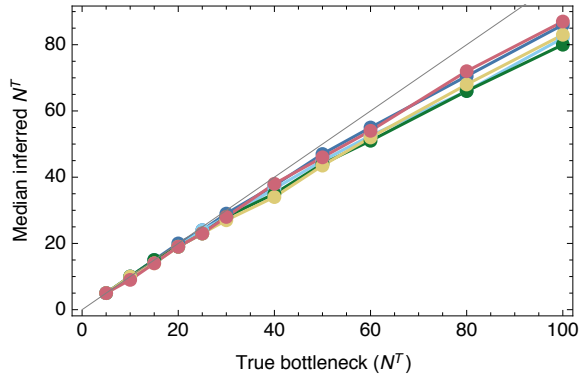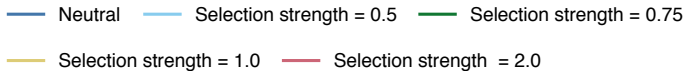

Supplement: S3 Fig — Inferences were made using either a neutral model, in which the effect of selection was assumed to be zero, or a selection model, which allowed scenarios involving selection to be identified. Median inferences are shown from 200 simulations, each involving three replicate transmission events, for each datapoint. (PDF) [file pgen.1007718.s003.pdf]

Neutral

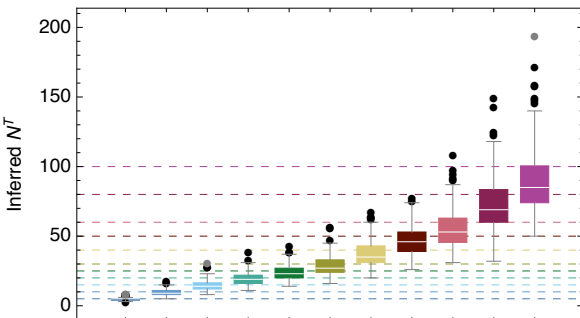

Selection strength = 0.5

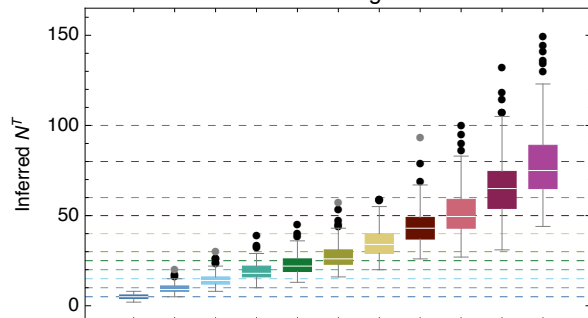

Selection strength = 0.75

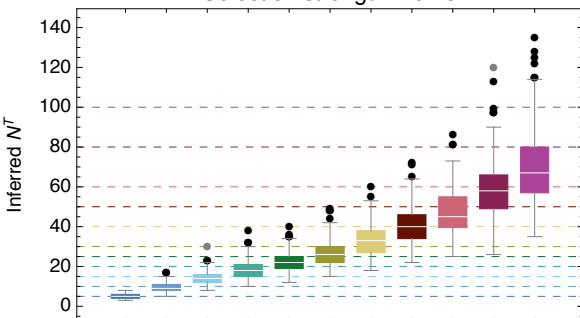

Selection strength = 1.0

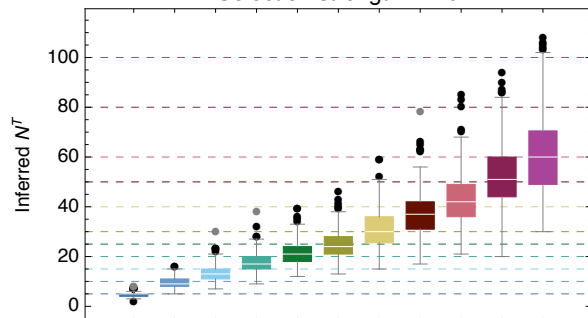

Selection strength = 2.0

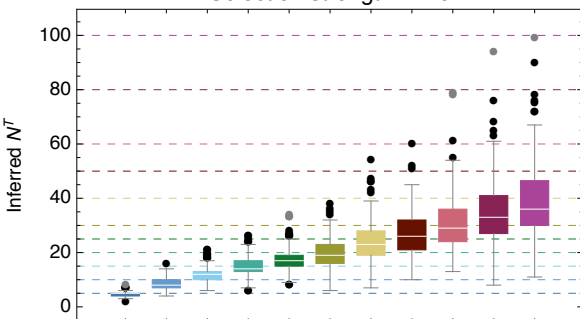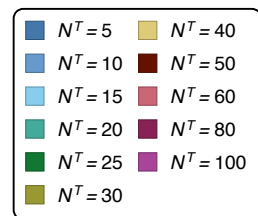

Supplement: S4 Fig — Results were generated by applying a neutral inference model to selected simulated data. Results are shown for 200 simulations at each bottleneck size. (PDF) [file pgen.1007718.s004.pdf]

Neutral

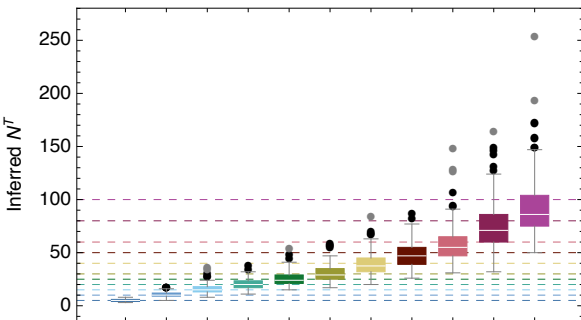

Selection strength = 0.5

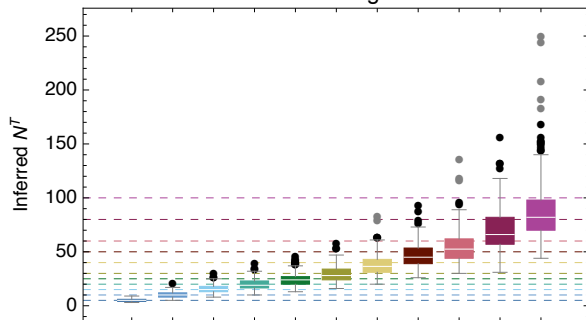

Selection strength = 0.75

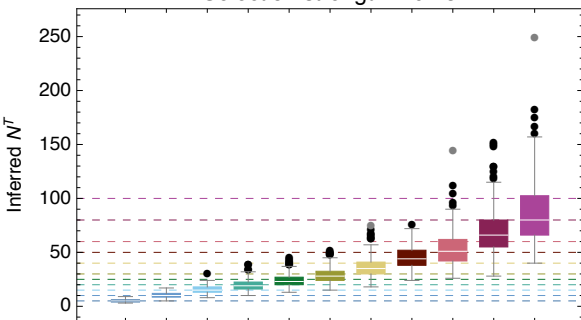

Selection strength = 1.0

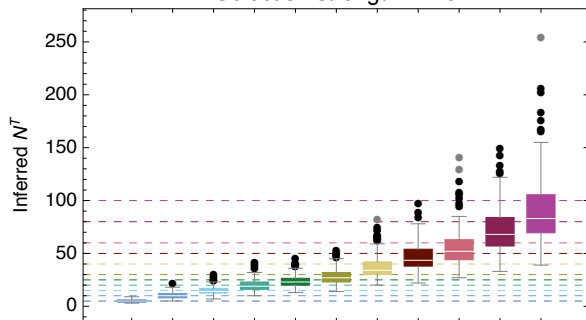

Selection strength = 2.0

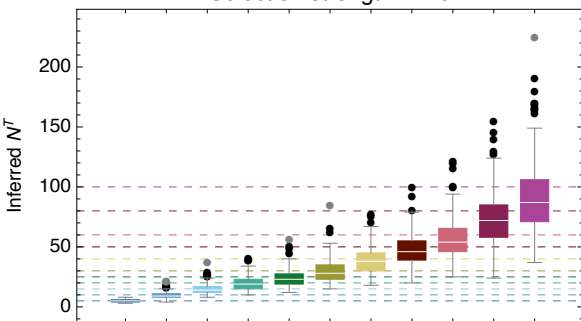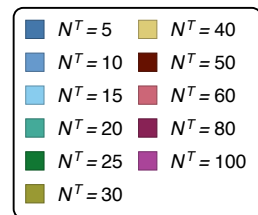

Supplement: S5 Fig — Results were generated by applying an inference model accounting for selection to selected simulated data. Results are shown for 200 simulations at each bottleneck size. (PDF) [file pgen.1007718.s005.pdf]

Neutral

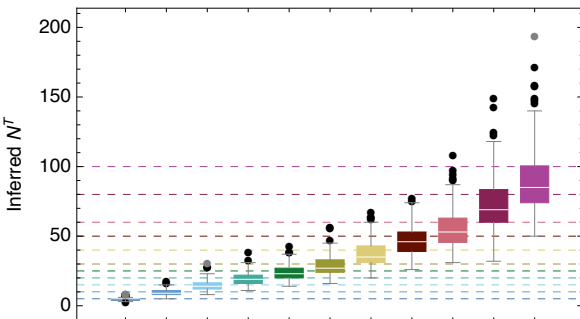

Selection strength = 0.5

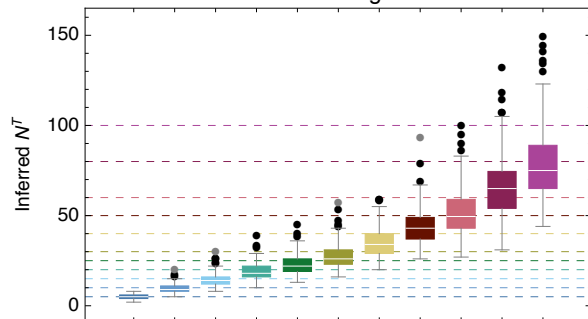

Selection strength = 0.75

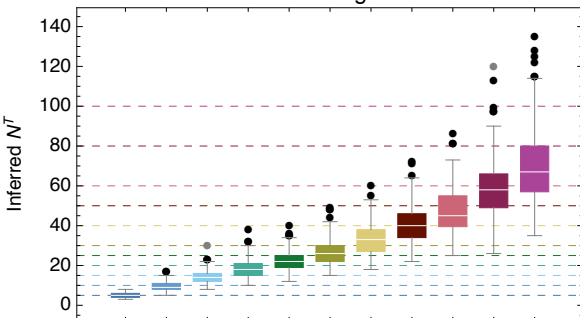

Selection strength = 1.0

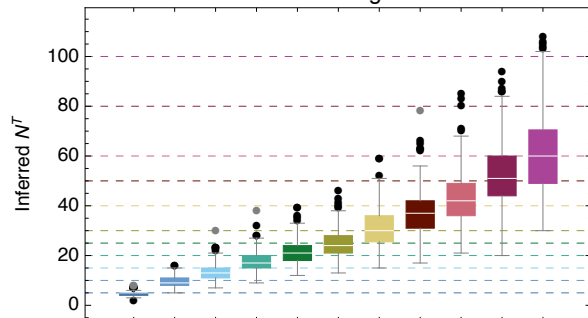

Selection strength = 2.0

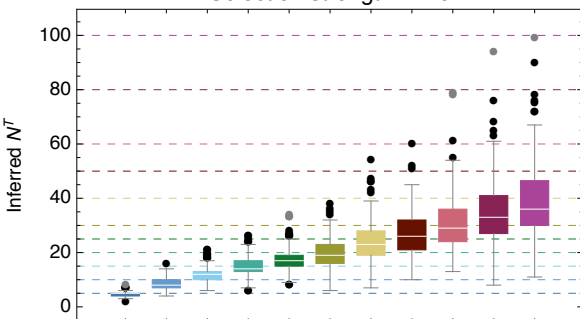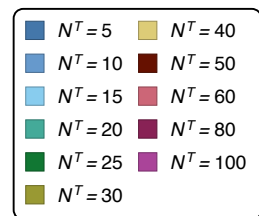

Supplement: S6 Fig — Results were generated by applying a neutral inference model to selected simulated data. Results are shown for 200 simulations at each bottleneck size, each simulation describing three replicate transmission events. (PDF) [file pgen.1007718.s006.pdf]

Neutral

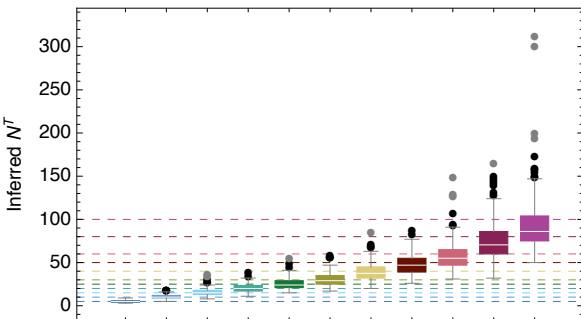

Selection strength = 0.5

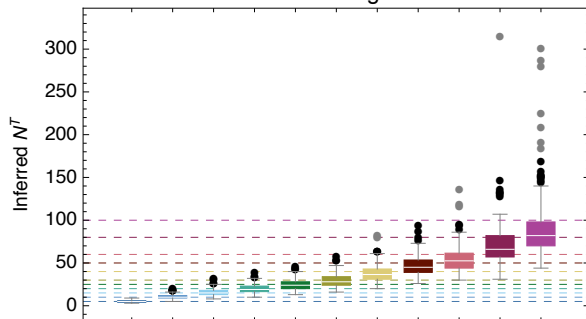

Selection strength = 0.75

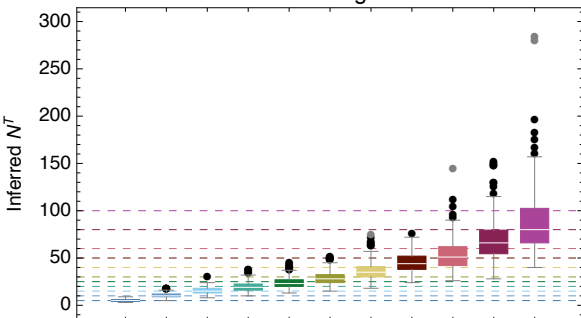

Selection strength = 1.0

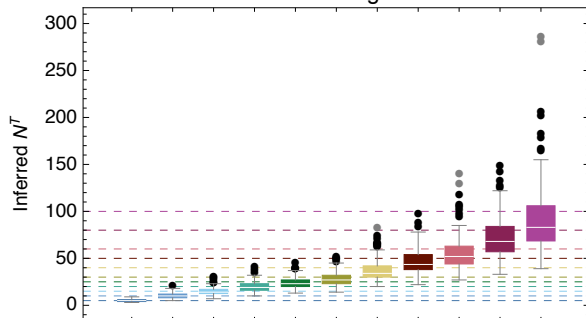

Selection strength = 2.0

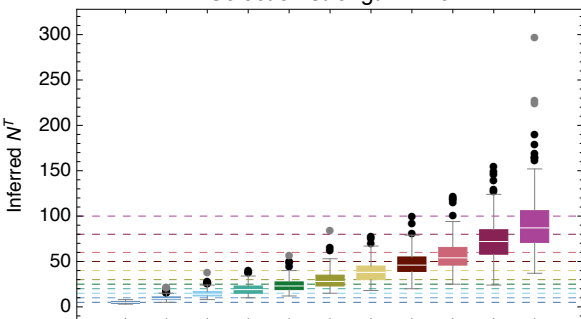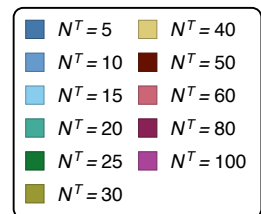

Supplement: S7 Fig — Results were generated by applying a neutral inference model to selected simulated data. Results are shown for 200 simulations at each bottleneck size, each simulation describing three replicate transmission events. (PDF) [file pgen.1007718.s007.pdf]

Selection strength = 0.75

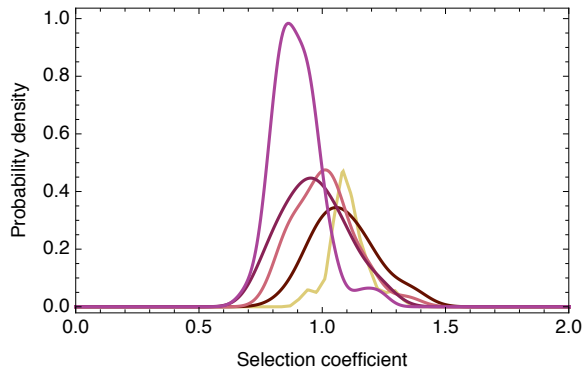

Selection strength = 1.0

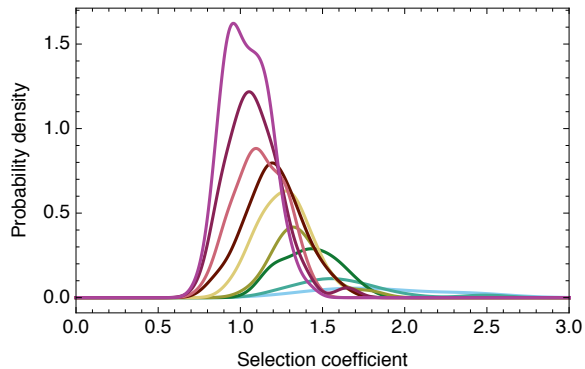

Selection strength = 2.0

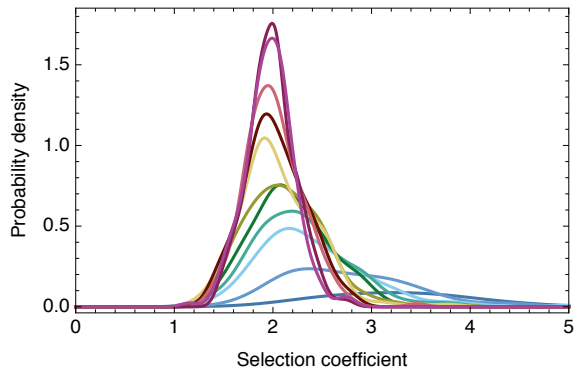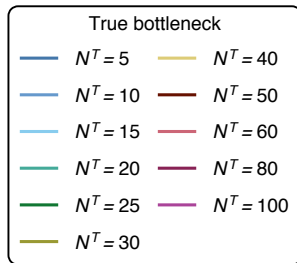

Supplement: S8 Fig — Distributions were constructed for bottleneck values where the inference of selection resulted in a true positive rate for identifying selected variants of above 5%. Smooth kernel distributions were computed as for Fig 7. (PDF) [file pgen.1007718.s008.pdf]

HA G400A

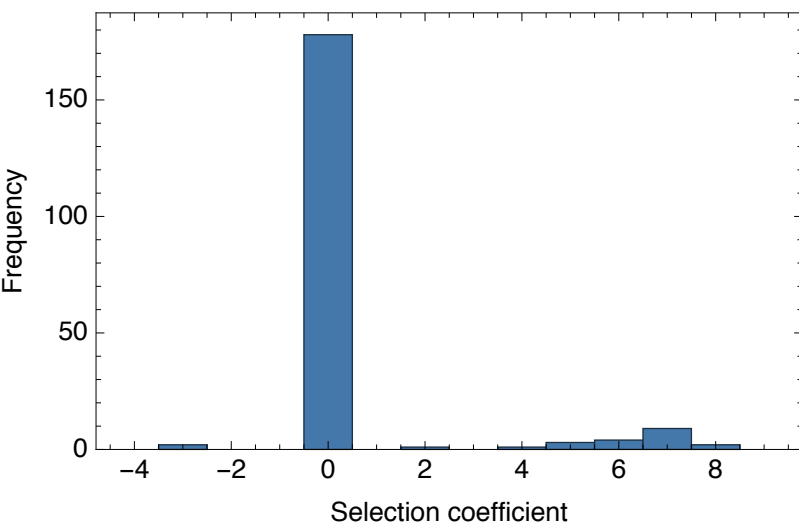

HA C550A

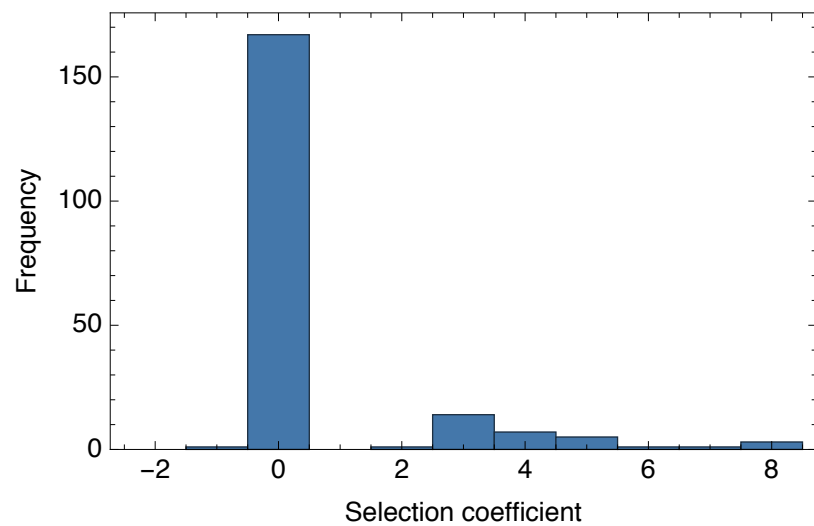

HA T634C

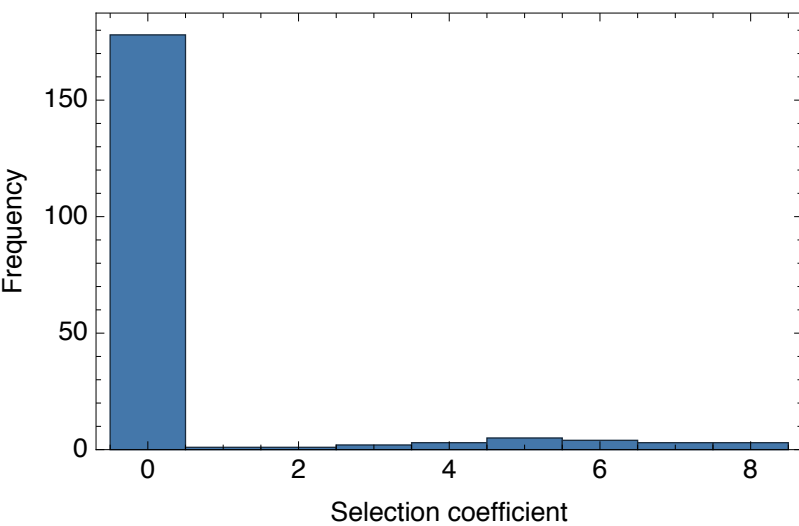

HA G748A

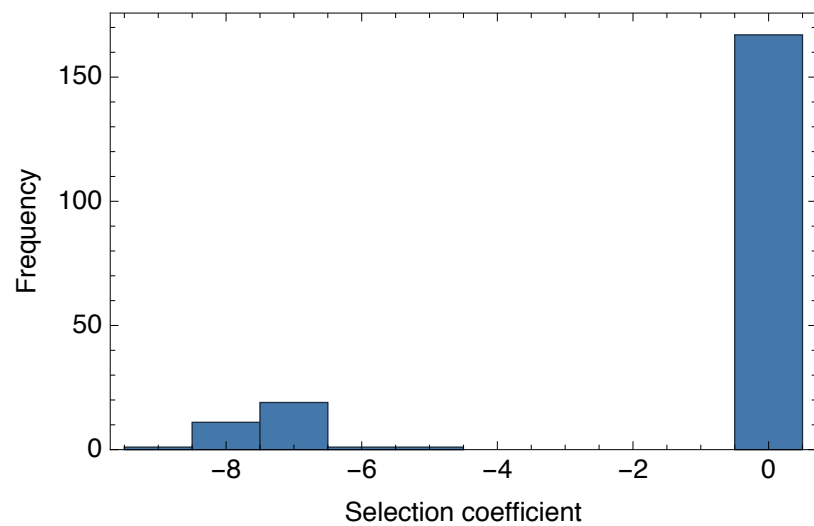

HA A868T

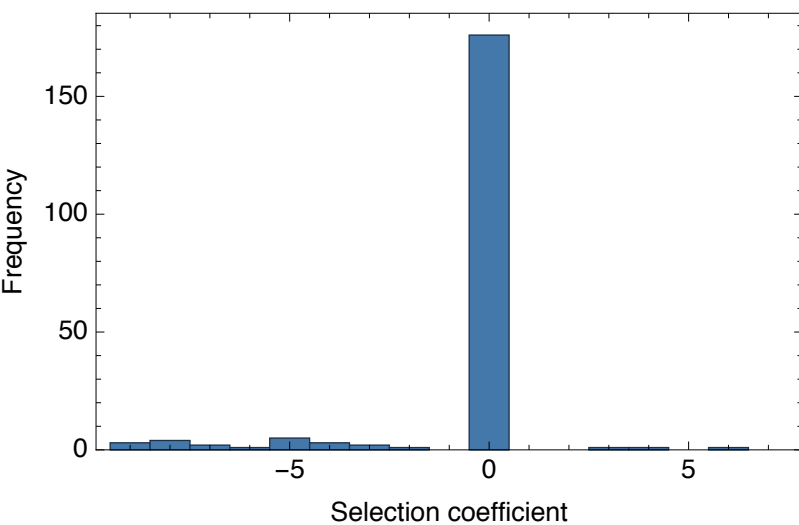

HA C1762T

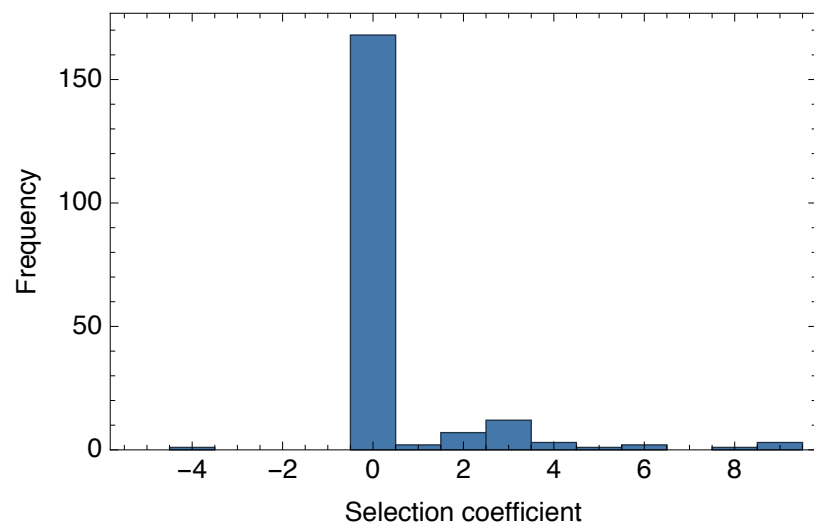

Supplement: S11 Fig — A replicate inference method was employed such that a common fitness landscape was imposed. Selection inferences that resulted in at least 10% non-zero inferences are here reported by the nucleotide position of the variant site. (PDF) [file pgen.1007718.s011.pdf]

HA190D225D  $q_{\text{cut}} = 0.03$ 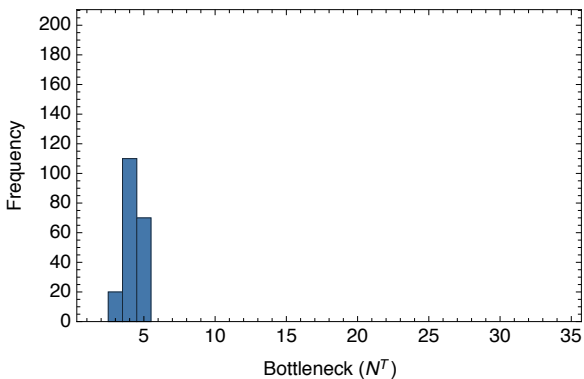HA190D225D  $q_{\text{cut}} = 0.04$ 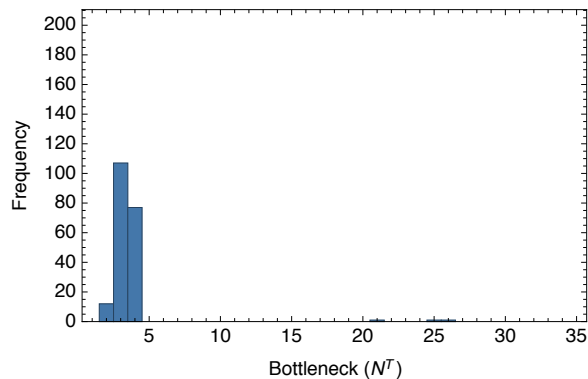Mut  $q_{\text{cut}} = 0.03$ 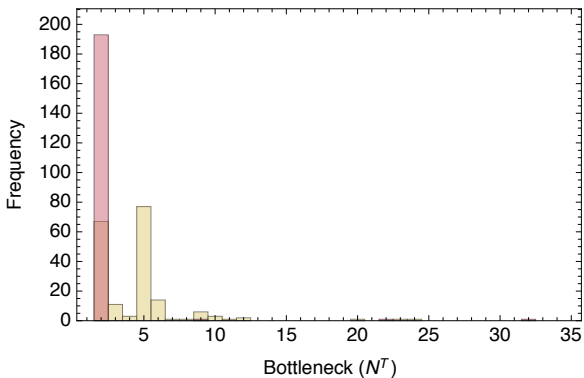Mut  $q_{\text{cut}} = 0.04$ 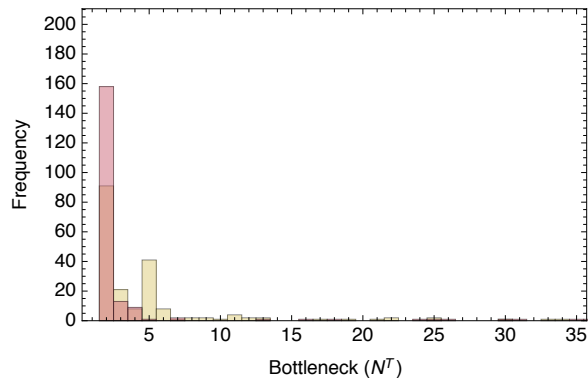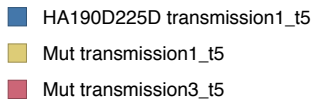

Supplement: S12 Fig — A replicate inference method was employed for the Mut transmission pairs such that a common fitness landscape was imposed. The Mut transmission pairs may take different bottleneck values and have been plotted as an overlapping histogram. Bottleneck inferences larger than NT = 35 have been omitted for clarity. (PDF) [file pgen.1007718.s012.pdf]

Neutral

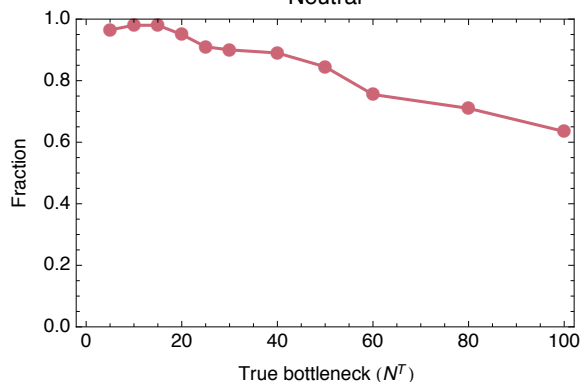

Selection strength = 0.5

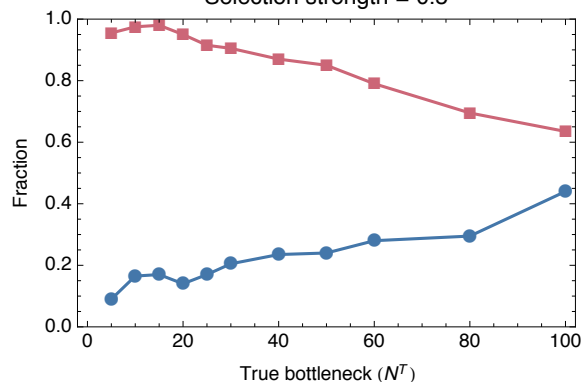

Selection strength = 0.75

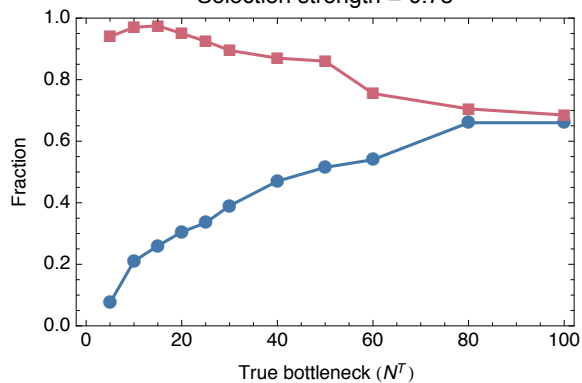

Selection strength = 1.0

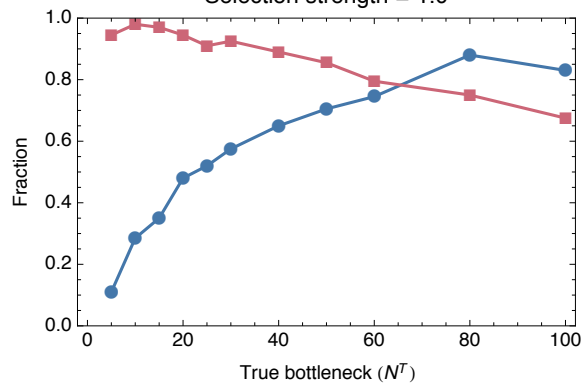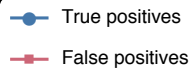

Supplement: S14 Fig — A fixed BIC difference of 10 units were employed in the model selection process, requiring a model with a single additional parameter to generate an improvement of at least 10 units to BIC to be accepted. While such a difference is accepted as showing strong evidence in favour of the more complex model, in our case it generated a high rate of false positive inferences of selection. (PDF) [file pgen.1007718.s014.pdf]

**A**

Bottleneck ratio

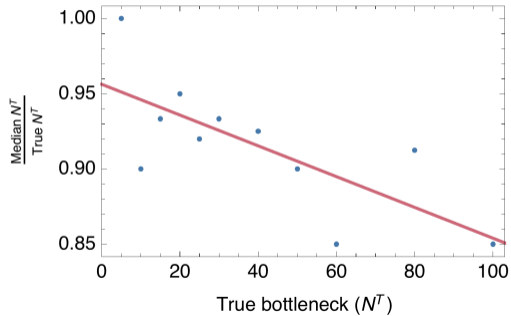**B**

BIC penalty function

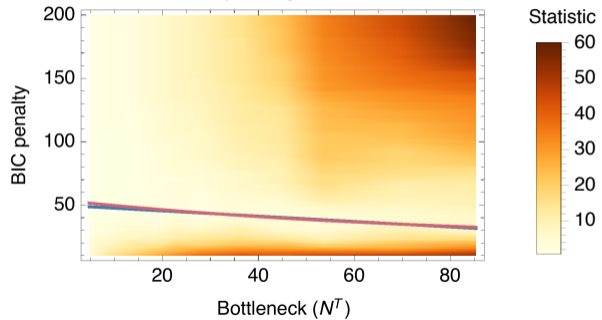

Supplement: S15 Fig — A) The ratio of the median inferred bottleneck to the true bottleneck is plotted against the true bottleneck size. As shown in Fig 3, as the bottleneck increases, our ability to infer it correctly decreases due to noise. In order to account for this phenomenon, a straight line is fitted to the data aiming to capture the general trend. B) Heat map of the bottleneck-specific statistic plotted against BIC penalty and bottleneck size. The plot was generated for three datasets with selection coefficients s = {0, 1, 2} and a simple statistic based on bottleneck differences was employed. More specifically, the median bottleneck was computed across 200 seeds and the bottleneck-statistic was defined as the absolute value of the difference between the median inferred bottleneck and the true bottleneck multiplied by the baseline determined in A). By considering bottlenecks in the range [5, 100] and BIC penalty values in the range [10, 200], a heat map was produced and linear and decay exponential regression were conducted seeking to minimise the sum of the statistic across the values of NT that were considered. (PDF) [file pgen.1007718.s015.pdf]
