# Supplementary material for: A novel framework for inferring parameters of transmission from viral sequence data
Source: PLoS Genet. 2018 Oct 16;14(10):e1007718. doi: 10.1371/journal.pgen.1007718 (PMC6203404; doi:10.1371/journal.pgen.1007718)
Supplement: S9 Fig — Haplotypes for which the inferred frequency rose to a frequency of at least 1% in at least one animal are shown. Haplotypes which are separated by a single mutation are joined by lines. (PDF) [file pgen.1007718.s009.pdf]

NA

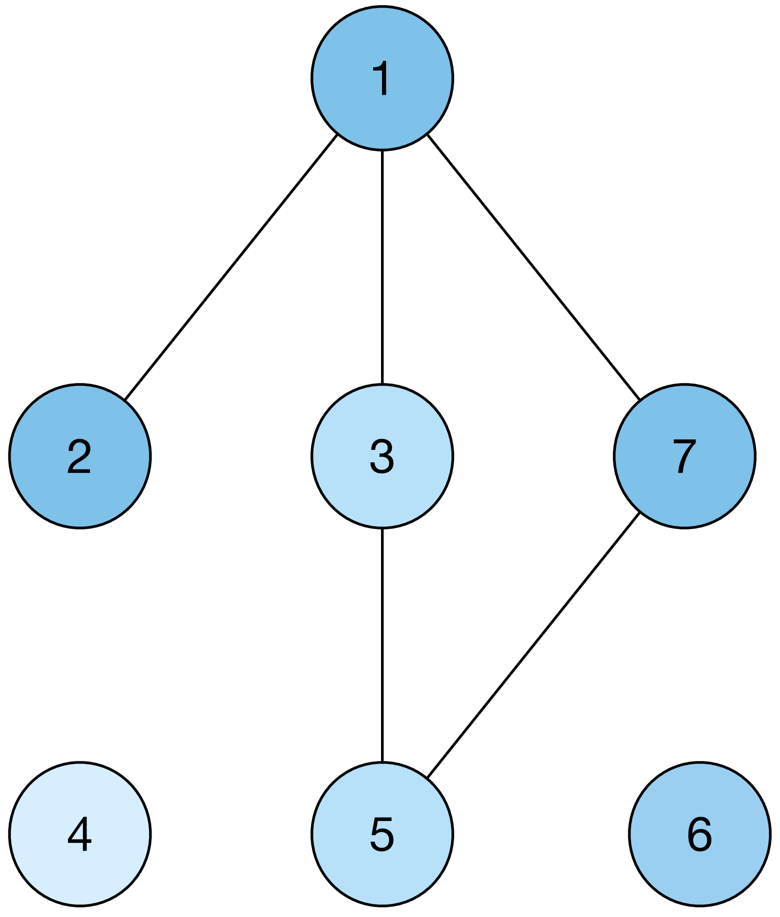

Variants		Haplotypes	
1	A45G	1	AGGCG
2	G440A	2	AGGAG
3	G649A	3	AAGCG
4	C1002A	4	AAACT
5	G1401T	5	GAGCG
		6	GGACA
		7	GGGCG

NP

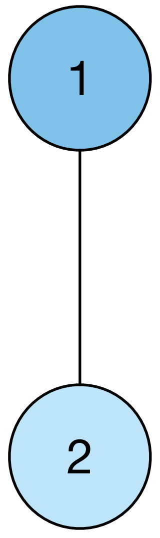

Variants		Haplotypes	
1	G600A	1	G
		2	A

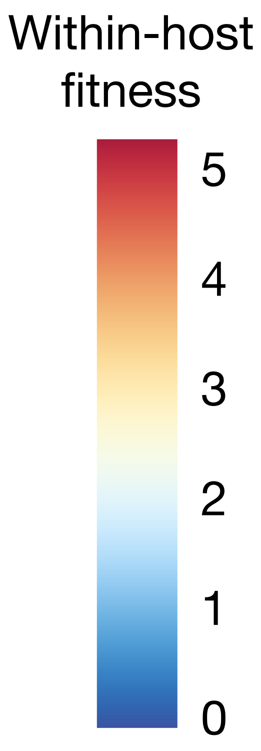

PA

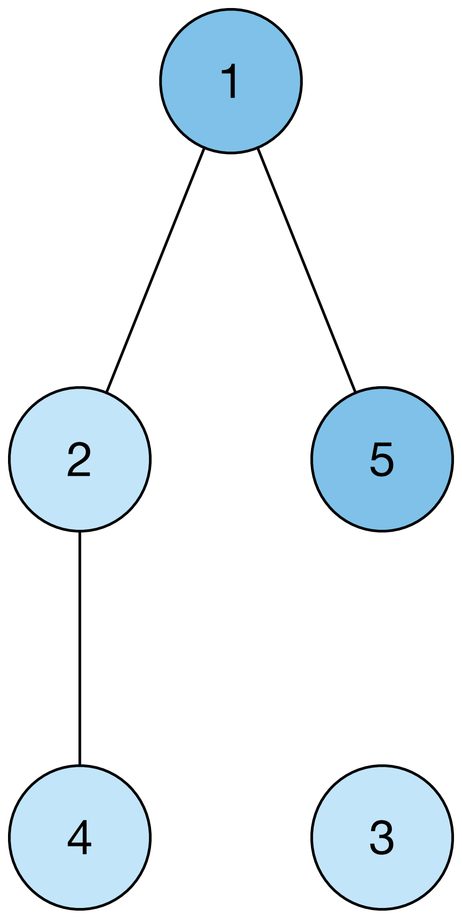

Variants		Haplotypes	
1	C234T	1	CACGG
2	A376G	2	CACGT
3	C465A	3	TAAGT
4	G781A	4	CGCGT
5	G1500T	5	CACAG

PB1

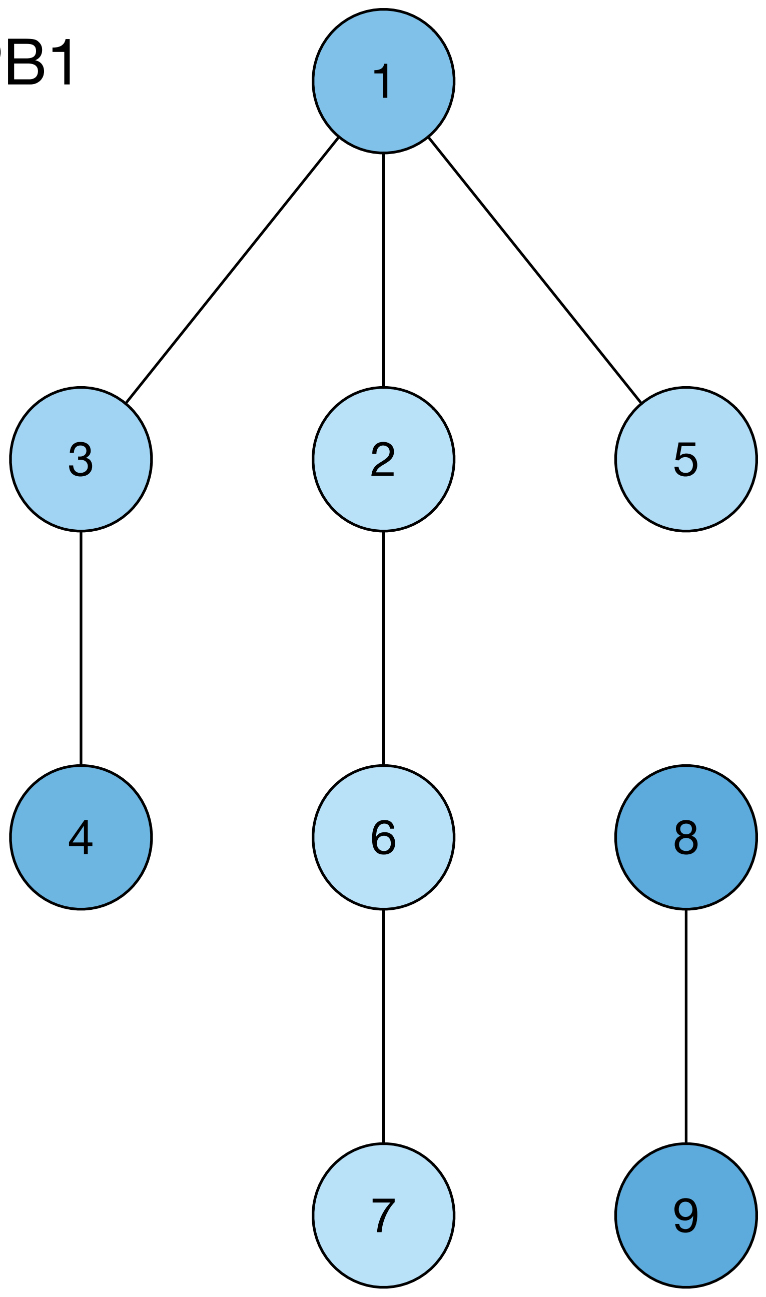

Variants		Haplotypes	
1	C65T	1	CCGACGTGC
2	C90A	2	CCGACTTGC
3	G265A	3	CCGAAGTGC
4	A558C	4	TCGAAGTGC
5	C835A	5	CCGACGGGC
6	G982T	6	CCGACTTAC
7	T1151G	7	CCAACTTAC
8	G1225A	8	CAGCCGTGC
9	C1512T	9	CAGCCGTGT

PB2

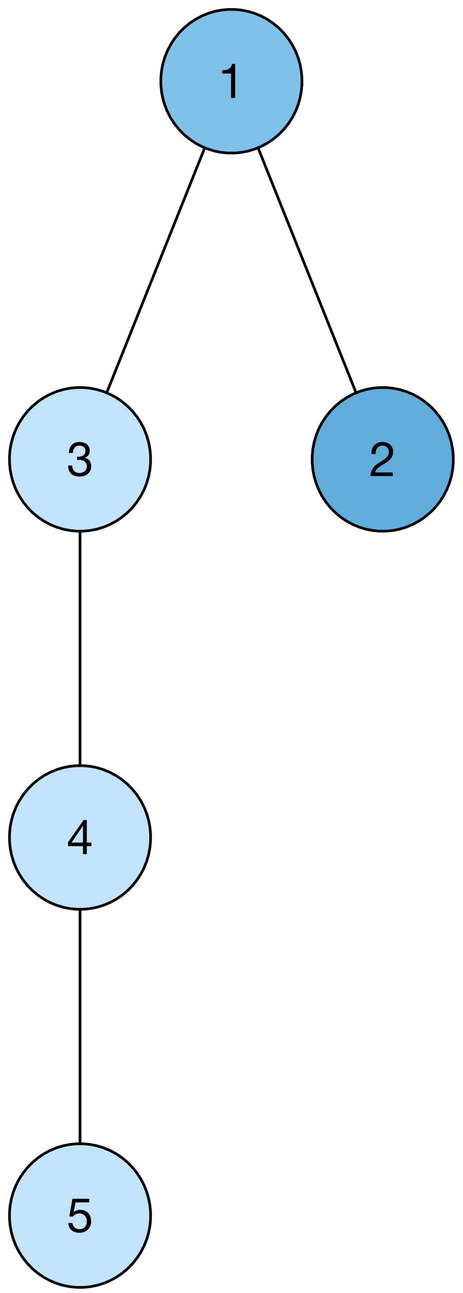

Variants		Haplotypes	
1	C651A	1	CTGAAACAGGGT
2	T1537C	2	CTGAAACAGGCT
3	G1930C	3	CCGAAACAGGGT
4	A1933C	4	CCGAAACAGGGC
5	A1943T	5	ACGAAACAGGGC
6	A1948G		
7	C1949G		
8	A1951G		
9	G1956T		
10	G1957A		
11	G2193C		
12	T2202C		

NS

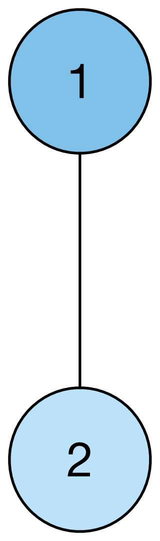

Variants		Haplotypes	
1	G298A	1	GA
2	A876T	2	AA
