# Supplementary material for: A novel framework for inferring parameters of transmission from viral sequence data
Source: PLoS Genet. 2018 Oct 16;14(10):e1007718. doi: 10.1371/journal.pgen.1007718 (PMC6203404; doi:10.1371/journal.pgen.1007718)
Supplement: S10 Fig — Haplotypes for which the inferred frequency rose to a frequency of at least 1% in at least one animal are shown. Haplotypes which are separated by a single mutation are joined by lines. (PDF) [file pgen.1007718.s010.pdf]

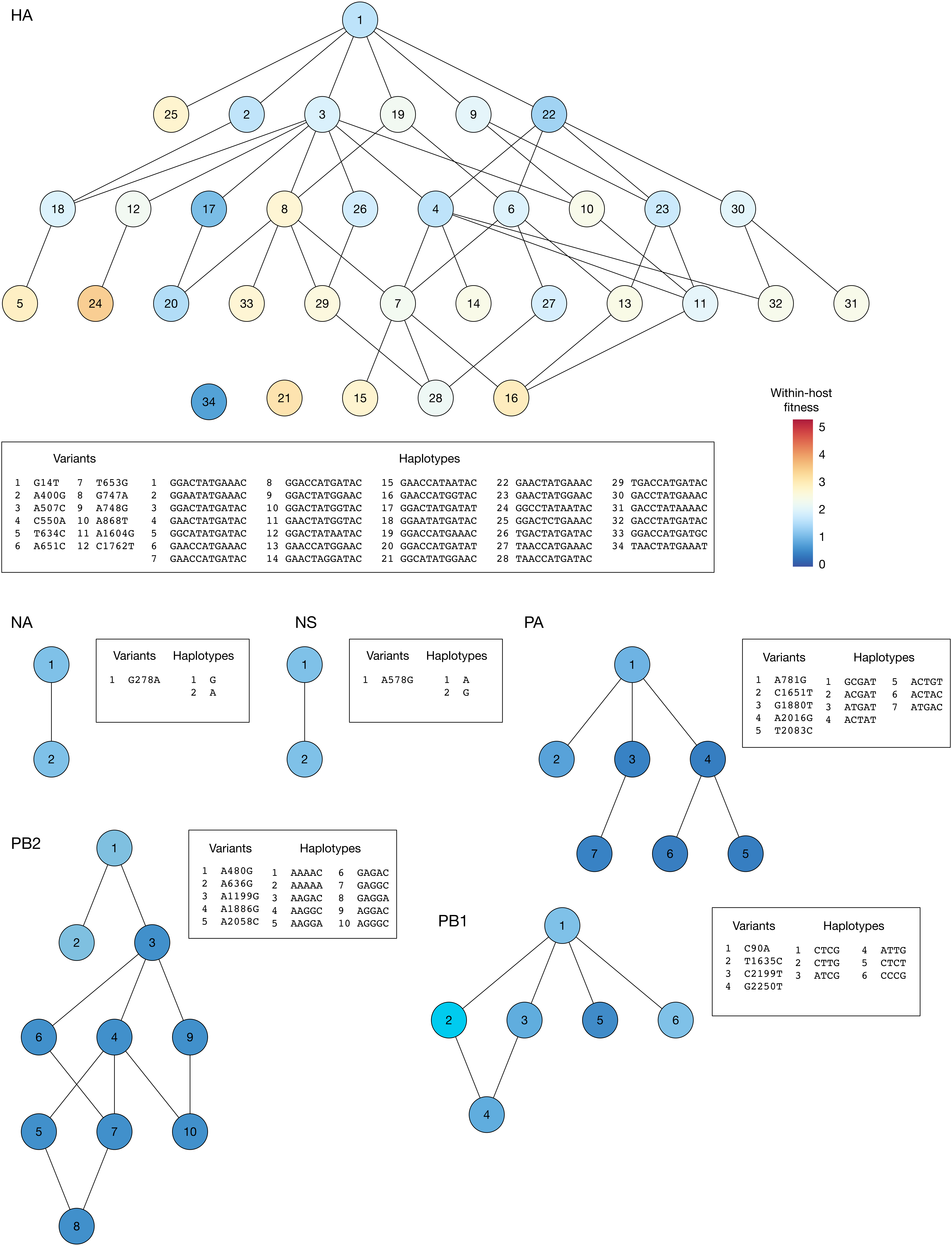
HA
Within-host fitness
5
4
3
2
1
0
Variants
1 G14T
2 A400G
3 A507C
4 C550A
5 T634C
6 A651C
7 T653G
8 G747A
9 A748G
10 A868T
11 A1604G
12 C1762T
Haplotypes
1 GGACTATGAAAC
2 GGAATATGAAAC
3 GGACTATGATAC
4 GAACTATGATAC
5 GGCATATGATAC
6 GAACCATGAAAC
7 GAACCATGATAC
8 GGACCATGATAC
9 GGACTATGGAAC
10 GGACTATGGTAC
11 GAACTATGGTAC
12 GGACTATAATAC
13 GAACCATGGAAC
14 GAACTAGGATAC
15 GAACCATAATAC
16 GAACCATGGTAC
17 GGACTATGATAT
18 GGAATATGATAC
19 GGACCATGAAAC
20 GGACCATGATAT
21 GGCATATGGAAC
22 GAACTATGAAAC
23 GAACTATGGAAC
24 GGCCTATAATAC
25 GGACTCTGAAAC
26 TGACTATGATAC
27 TAACCATGAAAC
28 TAACCATGATAC
29 TGACCATGATAC
30 GACCTATGAAAC
31 GACCTATAAAAC
32 GACCTATGATAC
33 GGACCATGATGC
34 TAACTATGAAAT
NA
Variants
1 G278A
Haplotypes
1 G
2 A
NS
Variants
1 A578G
Haplotypes
1 A
2 G
PA
Variants
1 A781G
2 C1651T
3 G1880T
4 A2016G
5 T2083C
Haplotypes
1 GCGAT
2 ACGAT
3 ATGAT
4 ACTAT
5 ACTGT
6 ACTAC
7 ATGAC
PB2
Variants
1 A480G
2 A636G
3 A1199G
4 A1886G
5 A2058C
Haplotypes
1 AAAAC
2 AAAAA
3 AAGAC
4 AAGGC
5 AAGGA
6 GAGAC
7 GAGGC
8 GAGGA
9 AGGAC
10 AGGGC
PB1
Variants
1 C90A
2 T1635C
3 C2199T
4 G2250T
Haplotypes
1 CTCG
2 CTTG
3 ATCG
4 ATTG
5 CTCT
6 CCCG
