# Supplementary material for: A novel framework for inferring parameters of transmission from viral sequence data
Source: PLoS Genet. 2018 Oct 16;14(10):e1007718. doi: 10.1371/journal.pgen.1007718 (PMC6203404; doi:10.1371/journal.pgen.1007718)
Supplement: S1 Text — We consider both the neutral case and that where selection applies within-host. (PDF) [file pgen.1007718.s016.pdf]

Supplementary Text 1

Derivation of Compound Distributions for N-Step Drift Process

1 Introduction

This considers the derivation of compound distributions for a general N-step drift process. Given a starting population size of N^T and a growth factor of λ the population size takes value $N(t) = N^T\lambda^t$ for $t = 1, 2, \ldots, N$. Note that we overload capital N: Baseline N refers to population sizes whilst super- or subscript N refers to the final time point of the within-host growth process. The specific composition of the viral population at each time point is governed by a multinomial sampling process in the viral frequencies at the preceding time point. A generalised probabilistic graph model for the system is shown in Figure 1. The below derivations assume familiarity with the methods presented in the main paper.

2 Neutral

Here we give a derivation for the compound distribution $\boldsymbol{x}^A_{\mathrm{PH},i}$ under a neutral model (no selection). As $\boldsymbol{q}^B$ is unknown we make the assumption that its true value is multivariate normal distributed with mean $\boldsymbol{\mu}^B$ and covariance matrix Σ^B, i.e. $\boldsymbol{q}^B \sim N(\boldsymbol{\mu}^B, \Sigma^B)$. As such $\boldsymbol{x}^B_{\mathrm{PH},i} \sim N\left(N^B_i T_i \boldsymbol{q}^B, \beta_i N^B_i M(T_i\boldsymbol{q}^B)\right)$ and $\boldsymbol{q}^F \sim N\left(\boldsymbol{q}^B, \frac{1}{N^T}M(\boldsymbol{q}^B)\right)$ where $\beta_i = \frac{N^B_i + C}{1+C}$. We now have that $\boldsymbol{q}^{G_1} \sim N\left(\boldsymbol{q}^F, \frac{1}{N^{G_1}}M(\boldsymbol{q}^F)\right) = N\left(\boldsymbol{q}^F, \frac{1}{\lambda N^T}M(\boldsymbol{q}^F)\right)$ with $N^{G_1} = \lambda N^T$. Consequently, $\boldsymbol{q}^{G_n} \sim N\left(\boldsymbol{q}^{G_{n-1}}, \frac{1}{N^{G_n}}M(\boldsymbol{q}^{G_{n-1}})\right) = N\left(\boldsymbol{q}^{G_{n-1}}, \frac{1}{\lambda^n N^T}M(\boldsymbol{q}^{G_{n-1}})\right)$ with $N^{G_n} = \lambda^n N^T$ for $n > 1$. Assuming N steps in the growth process (e.g. if one step = 12 hours, then $N = 2$ steps would correspond to a 24 hour difference between donor and recipient sampling times), we have $\boldsymbol{q}^A \sim N\left(\boldsymbol{q}^{G_{N-1}}, \frac{1}{N^{G_N}}M(\boldsymbol{q}^{G_{N-1}})\right)$. Finally, as always, $\boldsymbol{x}^A_{\mathrm{PH},i} \sim N\left(N^A_i T_i \boldsymbol{q}^A, \alpha_i N^A_i M(T_i\boldsymbol{q}^A)\right)$ where $\alpha_i = \frac{N^A_i + C}{1+C}$.

For the pre-transmission process, see main paper. For the post-transmission process, the marginalisation over $\boldsymbol{q}^B$ leads to

$$\mathrm{E}[\boldsymbol{q}^F] = \mathrm{E}[\mathrm{E}[\boldsymbol{q}^F|\boldsymbol{q}^B]] = \mathrm{E}[\boldsymbol{q}^B] = \boldsymbol{\mu}^B \tag{1}$$

and,

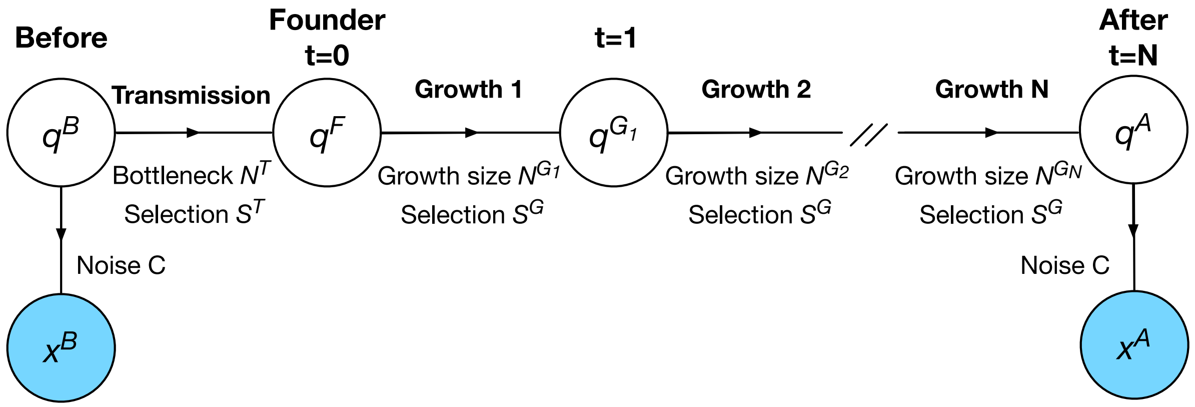

Figure 1. Probabilistic graph model for viral transmission and N-step within-host drift.

$$
\begin{aligned}
\operatorname{var}(\boldsymbol{q}^F) &= \mathrm{E}[\operatorname{var}[\boldsymbol{q}^F|\boldsymbol{q}^B]] + \operatorname{var}[\mathrm{E}[\boldsymbol{q}^F|\boldsymbol{q}^B]] \\
&= \mathrm{E}\left[\frac{1}{N^T}M(\boldsymbol{q}^B)\right] + \operatorname{var}\left[\boldsymbol{q}^B\right] \\
&= \frac{1}{N^T}M(\mathrm{E}[\boldsymbol{q}^B]) + \left(1-\frac{1}{N^T}\right)\operatorname{var}[\boldsymbol{q}^B] \\
&= \frac{1}{N^T}M(\boldsymbol{\mu}^B) + \left(1-\frac{1}{N^T}\right)\Sigma^B \\
&= \gamma_0 M(\boldsymbol{\mu}^B) + \delta_0\Sigma^B
\end{aligned}
\tag{2}
$$

where in the last step we defined $\gamma_0 = \frac{1}{N^T}$ and $\delta_0 = \left(1-\frac{1}{N^T}\right)$ (for reasons that will become clear later).

Next, for the $\boldsymbol{q}^F$ integral, the law of total expectation yields

$$
\mathrm{E}[\boldsymbol{q}^{G_1}] = \mathrm{E}[\mathrm{E}[\boldsymbol{q}^{G_1}|\boldsymbol{q}^F]] = \mathrm{E}[\boldsymbol{q}^F] = \boldsymbol{\mu}^B \tag{3}
$$

Next, under the law of total variance,

$$
\begin{aligned}
\operatorname{var}(\boldsymbol{q}^{G_1}) &= \mathrm{E}[\operatorname{var}[\boldsymbol{q}^{G_1}|\boldsymbol{q}^F]] + \operatorname{var}[\mathrm{E}[\boldsymbol{q}^{G_1}|\boldsymbol{q}^F]] \\
&= \mathrm{E}\left[\frac{1}{\lambda N^T}\left(\mathrm{Diag}(\boldsymbol{q}^F) - \boldsymbol{q}^F(\boldsymbol{q}^F)^\dagger\right)\right] + \operatorname{var}[\boldsymbol{q}^F] \\
&= \frac{1}{\lambda N^T}\left(\mathrm{Diag}(\mathrm{E}[\boldsymbol{q}^F]) - \mathrm{E}[\boldsymbol{q}^F]E[\boldsymbol{q}^F]^\dagger\right) + \left(1-\frac{1}{\lambda N^T}\right)\operatorname{var}[\boldsymbol{q}^F] \\
&= \frac{1}{\lambda N^T}M\left(\boldsymbol{\mu}^B\right) + \left(1-\frac{1}{\lambda N^T}\right)\left(\gamma_0 M(\boldsymbol{\mu}^B) + \delta_0\Sigma^B\right) \\
&= \left(\frac{1}{\lambda N^T} + \left(1-\frac{1}{\lambda N^T}\right)\gamma_0\right)M\left(\boldsymbol{\mu}^B\right) + \left(1-\frac{1}{\lambda N^T}\right)\delta_0\Sigma^B \\
&\equiv \gamma_1 M\left(\boldsymbol{\mu}^B\right) + \delta_1\Sigma^B
\end{aligned}
\tag{4}
$$

where in the last step we defined $\gamma_1 = \frac{1}{\lambda N^T} + \left(1-\frac{1}{\lambda N^T}\right)\gamma_0$ and $\delta_1 = \left(1-\frac{1}{\lambda N^T}\right)\delta_0$.

Continuing with the marginalisation over $\boldsymbol{q^{G_1}}$:

$$\mathrm{E}[\boldsymbol{q}^{G_2}] = \mathrm{E}[\mathrm{E}[\boldsymbol{q}^{G_2}|\boldsymbol{q}^{G_1}]] = \mathrm{E}[\boldsymbol{q}^{G_1}] = \boldsymbol{\mu}^B \tag{5}$$

and

$$\begin{aligned}
\mathrm{var}(\boldsymbol{q}^{G_2}) &= \mathrm{E}[\mathrm{var}[\boldsymbol{q}^{G_2}|\boldsymbol{q}^{G_1}]] + \mathrm{var}[\mathrm{E}[\boldsymbol{q}^{G_2}|\boldsymbol{q}^{G_1}]] \\
&= \mathrm{E}\left[\frac{1}{\lambda^2 N^T}\left(\mathrm{Diag}(\boldsymbol{q}^{G_1}) - \boldsymbol{q}^{G_1}(\boldsymbol{q}^{G_1})^\dagger\right)\right] + \mathrm{var}[\boldsymbol{q}^{G_1}] \\
&= \frac{1}{\lambda^2 N^T}\left(\mathrm{Diag}(\mathrm{E}[\boldsymbol{q}^{G_1}]) - \mathrm{E}[\boldsymbol{q}^{G_1}]E[\boldsymbol{q}^{G_1}]^\dagger\right) + \left(1 - \frac{1}{\lambda^2 N^T}\right)\mathrm{var}[\boldsymbol{q}^{G_1}] \\
&= \frac{1}{\lambda^2 N^T} M\left(\boldsymbol{\mu}^B\right) + \left(1 - \frac{1}{\lambda^2 N^T}\right)\left(\gamma_1 M\left(\boldsymbol{\mu}^B\right) + \delta_1 \Sigma^B\right) \\
&= \left(\frac{1}{\lambda^2 N^T} + \left(1 - \frac{1}{\lambda^2 N^T}\right)\gamma_1\right) M\left(\boldsymbol{\mu}^B\right) + \left(1 - \frac{1}{\lambda^2 N^T}\right)\delta_1 \Sigma^B \\
&\equiv \gamma_2 M\left(\boldsymbol{\mu}^B\right) + \delta_2 \Sigma^B
\end{aligned} \tag{6}$$

where in the last step we defined $\gamma_2 = \frac{1}{\lambda^2 N^T} + \left(1 - \frac{1}{\lambda^2 N^T}\right)\gamma_1$ and $\delta_1 = \left(1 - \frac{1}{\lambda^2 N^T}\right)\delta_1$.

From the above it is clear that general mean and variance expressions may derived for arbitrary $\boldsymbol{q}^{G_n}$:

$$\mathrm{E}[\boldsymbol{q}^{G_n}] = \boldsymbol{\mu}^B \tag{7}$$

$$\mathrm{var}(\boldsymbol{q}^{G_n}) = \gamma_n M\left(\boldsymbol{\mu}^B\right) + \delta_n \Sigma^B \tag{8}$$

where γ_n and δ_n obey the recurrence relations:

$$\gamma_n = \frac{1}{\lambda^n N^T} + \left(1 - \frac{1}{\lambda^n N^T}\right)\gamma_{n-1} \tag{9}$$

$$\delta_n = \left(1 - \frac{1}{\lambda^n N^T}\right)\delta_{n-1} \tag{10}$$

with $\gamma_0 = \frac{1}{N^T}$ and $\delta_0 = \left(1 - \frac{1}{N^T}\right)$.

Analytical solutions can be found using mathematical suites that allow for symbolic manipulation of recurrence relations:

$$\gamma_n = \frac{(-1)^n \lambda^{-\frac{n^2}{2}-\frac{n}{2}}\left(N^T\right)^{-n-1}(\lambda N^T;\lambda)_n \left(\lambda\left(N^T\right)^2\left(\sum_{j=0}^{n-1}\frac{(\lambda N^T-1)(-1)^{1-j}\lambda^{\frac{j^2}{2}+\frac{j}{2}-1}\left(N^T\right)^{j-1}}{(\lambda N^T;\lambda)_{j+1}}\right) + \lambda N^T - 1\right)}{\lambda N^T - 1} \tag{11}$$

$$\delta_n = (-1)^n \lambda^{-\frac{n^2}{2}-\frac{n}{2}}\left(N^T\right)^{-n-1}\left(N^T - 1\right)(\lambda N^T;\lambda)_n \tag{12}$$

where $(a;q)_n = \prod_{k=0}^{n-1}(1-aq^k)$ is the q-Pochhammer symbol. These expressions can be readily verified for low n.

To this end, assuming N steps in the growth process, the mean and variance of $\boldsymbol{q}^A$ are those of $\boldsymbol{q}^N$:

$$\mathrm{E}[\boldsymbol{q}^A] = \boldsymbol{\mu}^B \tag{13}$$

$$\mathrm{var}(\boldsymbol{q}^A) = \gamma_N M\left(\boldsymbol{\mu}^B\right) + \delta_N \Sigma^B \tag{14}$$

Finally we may compute the marginalisation over $\boldsymbol{q}^A$:

$$\mathrm{E}[\boldsymbol{x}^A_{\mathrm{PH,i}}] = \mathrm{E}[\mathrm{E}[\boldsymbol{x}^A_{\mathrm{PH},i}|\boldsymbol{q}^A]] = \mathrm{E}[N^A_i T_i \boldsymbol{q}^A] = N^A_i T_i \mathrm{E}[\boldsymbol{q}^A] = N^A_i T_i \boldsymbol{\mu}^B \tag{15}$$

and

$$\begin{aligned}
\mathrm{var}(\boldsymbol{x}^A_{\mathrm{PH},i}) &= \mathrm{E}[\mathrm{var}[\boldsymbol{x}^A_{\mathrm{PH},i}|\boldsymbol{q}^A]] + \mathrm{var}[\mathrm{E}[\boldsymbol{x}^A_{\mathrm{PH},i}|\boldsymbol{q}^A]] \\
&= \mathrm{E}\left[\alpha N^A_i M\left(T_i\boldsymbol{q}^A\right)\right] + \mathrm{var}[N^A_i T_i \boldsymbol{q}^A] \\
&= \alpha_i N^A_i \left(\mathrm{Diag}(\mathrm{E}\left[T_i\boldsymbol{q}^A\right] - \mathrm{E}\left[T_i\boldsymbol{q}^A\right]\mathrm{E}\left[T_i\boldsymbol{q}^A\right]^\dagger\right) + N^A_i\left(N^A_i - \alpha_i\right)\mathrm{var}[T_i\boldsymbol{q}^A] \\
&= \alpha_i N^A_i \left(\mathrm{Diag}\left(T_i\mathrm{E}\left[\boldsymbol{q}^A\right]\right) - T_i\mathrm{E}\left[\boldsymbol{q}^A\right]\left(T_i\mathrm{E}\left[\boldsymbol{q}^A\right]\right)^\dagger\right) + N^A_i\left(N^A_i - \alpha_i\right)T_i\mathrm{var}\left[\boldsymbol{q}^A\right]T_i^\dagger \\
&= \alpha_i N^A_i M\left(T_i\boldsymbol{\mu}^B\right)) + N^A_i\left(N^A_i - \alpha_i\right)T_i\left(\gamma_N M\left(\boldsymbol{\mu}^B\right) + \delta_N\Sigma^B\right)T_i^\dagger \\
&= N^A_i\left(\alpha_i + \left(N^A_i - \alpha_i\right)\gamma_N\right)M\left(T_i\boldsymbol{\mu}^B\right) + N^A_i\left(N^A_i - \alpha_i\right)\delta_N T_i\Sigma^B T_i^\dagger
\end{aligned} \tag{16}$$

where in the last step we used that $T_i\mathrm{Diag}(\boldsymbol{\mu}^B)T_i^\dagger = \mathrm{Diag}(T_i\boldsymbol{\mu}^B)$ which is true if T_i consists of zeroes and ones and if every column of T_i contains a single non-zero element, i.e. if a full haplotype can only contribute to a single partial haplotype in the set i. See Supplementary Text 2 for proof of this identity.

2.1 Generalisation

We note that the expressions for γ_n and δ_n may be generalised for arbitrary γ_0 and δ_0:

$$\gamma_n = \frac{(-1)^n\lambda^{-\frac{n^2}{2}-\frac{n}{2}}\left(N^T\right)^{-n}(\lambda N^T;\lambda)_n\left(\lambda N^T\left(\sum_{j=0}^{n-1}\frac{(\lambda N^T-1)(-1)^{1-j}\lambda^{\frac{j^2}{2}+\frac{j}{2}-1}\left(N^T\right)^{j-1}}{(\lambda N^T;\lambda)_{j+1}}\right) + \gamma_0\lambda N^T - \gamma_0\right)}{\lambda N^T - 1} \tag{17}$$

$$\delta_n = (-1)^n\delta_0\lambda^{-\frac{n^2}{2}-\frac{n}{2}}\left(N^T\right)^{-n}(\lambda N^T;\lambda)_n \tag{18}$$

3 Selection for Transmissibility

Deriving compound distributions under selection for transmissibility is a straightforward task. Here we have $\boldsymbol{q}^F \sim N\left(S^T(\boldsymbol{q}^B), \frac{1}{N^T}M(S^T(\boldsymbol{q}^B))\right)$ where $\beta_i = \frac{N^B_i + C}{1+C}$. As before, we have that $\boldsymbol{q}^{G_1} \sim N\left(\boldsymbol{q}^F, \frac{1}{N^{G_1}}M(\boldsymbol{q}^F)\right) = N\left(\boldsymbol{q}^F, \frac{1}{\lambda N^T}M(\boldsymbol{q}^F)\right)$ with $N^{G_1} = \lambda N^T$. Consequently, $\boldsymbol{q}^{G_n} \sim N\left(\boldsymbol{q}^{G_{n-1}}, \frac{1}{N^{G_n}}M(\boldsymbol{q}^{G_{n-1}})\right) = N\left(\boldsymbol{q}^{G_{n-1}}, \frac{1}{\lambda^n N^T}M(\boldsymbol{q}^{G_{n-1}})\right)$ with $N^{G_n} = \lambda^n N^T$ for $n > 1$. Assuming N steps in the growth process (e.g. if one step = 12 hours, then N=2 steps would correspond to a 24 hour difference between donor and recipient sampling times), then $\boldsymbol{q}^A \sim N\left(\boldsymbol{q}^{G_{N-1}}, \frac{1}{N^{G_N}}M(\boldsymbol{q}^{G_{N-1}})\right)$. Finally, as always, $\boldsymbol{x}^A_{\mathrm{PH},i} \sim N\left(N^A_i T_i\boldsymbol{q}^A, \alpha_i N^A_i M(T_i\boldsymbol{q}^A\right)$ where $\alpha_i = \frac{N^A_i + C}{1+C}$.

Effectively this only changes the marginalisation over $\boldsymbol{q}^B$:

$$\mathrm{E}[\boldsymbol{q}^F] = \mathrm{E}[\mathrm{E}[\boldsymbol{q}^F|\boldsymbol{q}^B]] = \mathrm{E}[S^T(\boldsymbol{q}^B)] \approx S^T(\mathrm{E}[\boldsymbol{q}^B]) = S^T(\boldsymbol{\mu}^B) \tag{19}$$

where in the penultimate step we used the first-order second-moment approximation to a vector function acting on a random variable. The law of total variance yields

$$\begin{aligned}
\mathrm{var}(\boldsymbol{q}^F) &= \mathrm{E}[\mathrm{var}[\boldsymbol{q}^F|\boldsymbol{q}^B]] + \mathrm{var}[\mathrm{E}[\boldsymbol{q}^F|\boldsymbol{q}^B]] \\
&= \mathrm{E}\left[\frac{1}{N^T} M(S^T(\boldsymbol{q}^B))\right] + \mathrm{var}\left[S^T(\boldsymbol{q}^B)\right] \\
&= \frac{1}{N^T} M(\mathrm{E}[S^T(\boldsymbol{q}^B)]) + \left(1 - \frac{1}{N^T}\right) \mathrm{var}[S^T(\boldsymbol{q}^B)] \\
&\approx \frac{1}{N^T} M(S^T(\mathrm{E}[\boldsymbol{q}^B])) + \left(1 - \frac{1}{N^T}\right) \left(DS^T\big|_{\mathrm{E}[\boldsymbol{q}^B]}\right) \mathrm{var}[\boldsymbol{q}^B] \left(DS^T\big|_{\mathrm{E}[\boldsymbol{q}^B]}\right)^\dagger \\
&= \frac{1}{N^T} M(S^T(\boldsymbol{\mu}^B)) + \left(1 - \frac{1}{N^T}\right) \left(DS^T\big|_{\boldsymbol{\mu}^B}\right) \Sigma^B \left(DS^T\big|_{\boldsymbol{\mu}^B}\right)^\dagger \\
&= \gamma_0 M(S^T(\boldsymbol{\mu}^B)) + \delta_0 \left(DS^T\big|_{\boldsymbol{\mu}^B}\right) \Sigma^B \left(DS^T\big|_{\boldsymbol{\mu}^B}\right)^\dagger
\end{aligned} \tag{20}$$

where in the last step we defined $\gamma_0 = \frac{1}{N^T}$ and $\delta_0 = \left(1 - \frac{1}{N^T}\right)$. We also note that $(DS)_i^j = \frac{\partial S_i}{\partial q_j}$ is the Jacobian matrix arising from the first-order second-moment approximation.

Next, for the $\boldsymbol{q}^F$ integral, the law of total expectation yields

$$\mathrm{E}[\boldsymbol{q}^{G_1}] = \mathrm{E}[\mathrm{E}[\boldsymbol{q}^{G_1}|\boldsymbol{q}^F]] = \mathrm{E}[\boldsymbol{q}^F] = S^T(\boldsymbol{\mu}^B) \tag{21}$$

Next, under the law of total variance,

$$\begin{aligned}
\mathrm{var}(\boldsymbol{q}^{G_1}) &= \mathrm{E}[\mathrm{var}[\boldsymbol{q}^{G_1}|\boldsymbol{q}^F]] + \mathrm{var}[\mathrm{E}[\boldsymbol{q}^{G_1}|\boldsymbol{q}^F]] \\
&= \mathrm{E}\left[\frac{1}{\lambda N^T}\left(\mathrm{Diag}(\boldsymbol{q}^F) - \boldsymbol{q}^F(\boldsymbol{q}^F)^\dagger\right)\right] + \mathrm{var}[\boldsymbol{q}^F] \\
&= \frac{1}{\lambda N^T}\left(\mathrm{Diag}(\mathrm{E}[\boldsymbol{q}^F]) - \mathrm{E}[\boldsymbol{q}^F]E[\boldsymbol{q}^F]^\dagger\right) + \left(1 - \frac{1}{\lambda N^T}\right) \mathrm{var}[\boldsymbol{q}^F] \\
&= \frac{1}{\lambda N^T} M\left(S^T(\boldsymbol{\mu}^B)\right) + \left(1 - \frac{1}{\lambda N^T}\right)\left(\gamma_0 M(S^T(\boldsymbol{\mu}^B)) + \delta_0 \left(DS^T\big|_{\boldsymbol{\mu}^B}\right) \Sigma^B \left(DS^T\big|_{\boldsymbol{\mu}^B}\right)^\dagger\right) \\
&= \left(\frac{1}{\lambda N^T} + \left(1 - \frac{1}{\lambda N^T}\right)\gamma_0\right) M\left(S^T(\boldsymbol{\mu}^B)\right) + \left(1 - \frac{1}{\lambda N^T}\right)\delta_0 \left(DS^T\big|_{\boldsymbol{\mu}^B}\right) \Sigma^B \left(DS^T\big|_{\boldsymbol{\mu}^B}\right)^\dagger \\
&\equiv \gamma_1 M\left(S^T(\boldsymbol{\mu}^B)\right) + \delta_1 \left(DS^T\big|_{\boldsymbol{\mu}^B}\right) \Sigma^B \left(DS^T\big|_{\boldsymbol{\mu}^B}\right)^\dagger
\end{aligned} \tag{22}$$

where in the last step we defined $\gamma_1 = \frac{1}{\lambda N^T} + \left(1 - \frac{1}{\lambda N^T}\right)\gamma_0$ and $\delta_1 = \left(1 - \frac{1}{\lambda N^T}\right)\delta_0$.

Continuing with the marginalisation over $\boldsymbol{q}^{\boldsymbol{G_1}}$:

$$\mathrm{E}[\boldsymbol{q}^{G_2}] = \mathrm{E}[\mathrm{E}[\boldsymbol{q}^{G_2}|\boldsymbol{q}^{G_1}]] = \mathrm{E}[\boldsymbol{q}^{G_1}] = S^T(\boldsymbol{\mu}^B) \tag{23}$$

and

$$
\begin{aligned}
\mathrm{var}(\boldsymbol{q}^{G_2}) &= \mathrm{E}[\mathrm{var}[\boldsymbol{q}^{G_2}|\boldsymbol{q}^{G_1}]] + \mathrm{var}[\mathrm{E}[\boldsymbol{q}^{G_2}|\boldsymbol{q}^{G_1}]] \\
&= \mathrm{E}\left[\frac{1}{\lambda^2 N^T}\left(\mathrm{Diag}(\boldsymbol{q}^{G_1}) - \boldsymbol{q}^{G_1}(\boldsymbol{q}^{G_1})^\dagger\right)\right] + \mathrm{var}[\boldsymbol{q}^{G_1}] \\
&= \frac{1}{\lambda^2 N^T}\left(\mathrm{Diag}(\mathrm{E}[\boldsymbol{q}^{G_1}]) - \mathrm{E}[\boldsymbol{q}^{G_1}]E[\boldsymbol{q}^{G_1}]^\dagger\right) + \left(1 - \frac{1}{\lambda^2 N^T}\right)\mathrm{var}[\boldsymbol{q}^{G_1}] \\
&= \frac{1}{\lambda^2 N^T} M\left(S^T(\boldsymbol{\mu}^B)\right) + \left(1 - \frac{1}{\lambda^2 N^T}\right)\left(\gamma_1 M\left(S^T(\boldsymbol{\mu}^B)\right) + \delta_1\left(DS^T\big|_{\boldsymbol{\mu}^B}\right)\Sigma^B\left(DS^T\big|_{\boldsymbol{\mu}^B}\right)^\dagger\right) \\
&= \left(\frac{1}{\lambda^2 N^T} + \left(1 - \frac{1}{\lambda^2 N^T}\right)\gamma_1\right) M\left(S^T(\boldsymbol{\mu}^B)\right) + \left(1 - \frac{1}{\lambda^2 N^T}\right)\delta_1\left(DS^T\big|_{\boldsymbol{\mu}^B}\right)\Sigma^B\left(DS^T\big|_{\boldsymbol{\mu}^B}\right)^\dagger \\
&\equiv \gamma_2 M\left(S^T(\boldsymbol{\mu}^B)\right) + \delta_2\left(DS^T\big|_{\boldsymbol{\mu}^B}\right)\Sigma^B\left(DS^T\big|_{\boldsymbol{\mu}^B}\right)^\dagger
\end{aligned} \tag{24}
$$

where in the last step we defined $\gamma_2 = \frac{1}{\lambda^2 N^T} + \left(1 - \frac{1}{\lambda^2 N^T}\right)\gamma_1$ and $\delta_1 = \left(1 - \frac{1}{\lambda^2 N^T}\right)\delta_1$.

From the above it is clear that general mean and variance expressions may derived for arbitrary $\boldsymbol{q}^{G_n}$:

$$
\mathrm{E}[\boldsymbol{q}^{G_n}] = S^T(\boldsymbol{\mu}^B) \tag{25}
$$

$$
\mathrm{var}(\boldsymbol{q}^{G_n}) = \gamma_n M\left(S^T(\boldsymbol{\mu}^B)\right) + \delta_n\left(DS^T\big|_{\boldsymbol{\mu}^B}\right)\Sigma^B\left(DS^T\big|_{\boldsymbol{\mu}^B}\right)^\dagger \tag{26}
$$

where γ_n and δ_n obey the recurrence relations:

$$
\gamma_n = \frac{1}{\lambda^n N^T} + \left(1 - \frac{1}{\lambda^n N^T}\right)\gamma_{n-1} \tag{27}
$$

$$
\delta_n = \left(1 - \frac{1}{\lambda^n N^T}\right)\delta_{n-1} \tag{28}
$$

with $\gamma_0 = \frac{1}{N^T}$ and $\delta_0 = \left(1 - \frac{1}{N^T}\right)$. The solutions to these recurrence relations are given in Equations 11 and 12.

To this end, assuming N steps in the growth process, the mean and variance of $\boldsymbol{q}^A$ are those of $\boldsymbol{q}^N$:

$$
\mathrm{E}[\boldsymbol{q}^A] = S^T(\boldsymbol{\mu}^B) \tag{29}
$$

$$
\mathrm{var}(\boldsymbol{q}^A) = \gamma_N M\left(S^T(\boldsymbol{\mu}^B)\right) + \delta_N\left(DS^T\big|_{\boldsymbol{\mu}^B}\right)\Sigma^B\left(DS^T\big|_{\boldsymbol{\mu}^B}\right)^\dagger \tag{30}
$$

Finally we may compute the marginalisation over $\boldsymbol{q}^A$:

$$
\mathrm{E}[\boldsymbol{x}^A_{\mathrm{PH,i}}] = \mathrm{E}[\mathrm{E}[\boldsymbol{x}^A_{\mathrm{PH},i}|\boldsymbol{q}^A]] = \mathrm{E}[N^A_i T_i \boldsymbol{q}^A] = N^A_i T_i \mathrm{E}[\boldsymbol{q}^A] = N^A_i T_i S^T(\boldsymbol{\mu}^B) \tag{31}
$$

and

$$
\begin{aligned}
\mathrm{var}(\boldsymbol{x}^A_{\mathrm{PH},i}) &= \mathrm{E}[\mathrm{var}[\boldsymbol{x}^A_{\mathrm{PH},i}|\boldsymbol{q}^A]] + \mathrm{var}[\mathrm{E}[\boldsymbol{x}^A_{\mathrm{PH},i}|\boldsymbol{q}^A]] \\
&= \mathrm{E}\left[\alpha N_i^A M\left(T_i\boldsymbol{q}^A\right)\right] + \mathrm{var}[N_i^A T_i \boldsymbol{q}^A] \\
&= \alpha_i N_i^A \left(\mathrm{Diag}(\mathrm{E}\left[T_i\boldsymbol{q}^A\right] - \mathrm{E}\left[T_i\boldsymbol{q}^A\right]\mathrm{E}\left[T_i\boldsymbol{q}^A\right]^\dagger\right) + N_i^A\left(N_i^A - \alpha_i\right)\mathrm{var}[T_i\boldsymbol{q}^A] \\
&= \alpha_i N_i^A \left(\mathrm{Diag}\left(T_i\mathrm{E}\left[\boldsymbol{q}^A\right]\right) - T_i\mathrm{E}\left[\boldsymbol{q}^A\right]\left(T_i\mathrm{E}\left[\boldsymbol{q}^A\right]\right)^\dagger\right) + N_i^A\left(N_i^A - \alpha_i\right)T_i\mathrm{var}\left[\boldsymbol{q}^A\right]T_i^\dagger \\
&= \alpha_i N_i^A M\left(T_i S^T(\boldsymbol{\mu}^B))\right) + N_i^A\left(N_i^A - \alpha_i\right)T_i\left(\gamma_N M\left(S^T(\boldsymbol{\mu}^B)\right) + \delta_N\left(DS^T\big|_{\boldsymbol{\mu}^B}\right)\Sigma^B\left(DS^T\big|_{\boldsymbol{\mu}^B}\right)^\dagger\right)T_i^\dagger \\
&= N_i^A\left(\alpha_i + \left(N_i^A - \alpha_i\right)\gamma_N\right)M\left(T_i S^T(\boldsymbol{\mu}^B)\right) + N_i^A\left(N_i^A - \alpha_i\right)\delta_N T_i\left(DS^T\big|_{\boldsymbol{\mu}^B}\right)\Sigma^B\left(DS^T\big|_{\boldsymbol{\mu}^B}\right)^\dagger T_i^\dagger
\end{aligned}
\tag{32}
$$

where in the last step we used that $T_i\mathrm{Diag}(S^T(\boldsymbol{\mu}^B))T_i^\dagger = \mathrm{Diag}(T_i S^T(\boldsymbol{\mu}^B))$.

4 Selection for Within-Host Adaptation

Deriving compound distributions under selection for within-host adaptation is a little more tricky. Two approaches can be taken: A) We assume that selection only acts after all the growth steps, i.e. it acts in the $\boldsymbol{q}^A$ compound. This can straightforwardly be derived by combining results from the main paper with the new coefficients γ_n and δ_n. B) We assume that selection acts once every 12 hours of growth, i.e. we have interleaving drift and selection processes: $\boldsymbol{q}^F \to \boldsymbol{q}^{G_1} \to S^G(\boldsymbol{q}^{G_1}) \to \boldsymbol{q}^{G_2} \to S^G(\boldsymbol{q}^{G_2}) \to \ldots$. In the below we use the latter approach to derive compound solutions in the absence of selection for transmissibility.

As previously we have $\boldsymbol{q}^F \sim N\left(\boldsymbol{q}^B, \frac{1}{N^T}M(\boldsymbol{q}^B)\right)$ with $\beta_i = \frac{N_i^B + C}{1+C}$ and $\boldsymbol{q}^{G_1} \sim N\left(\boldsymbol{q}^F, \frac{1}{N^{G_1}}M(\boldsymbol{q}^F)\right) = N\left(\boldsymbol{q}^F, \frac{1}{\lambda N^T}M(\boldsymbol{q}^F)\right)$ with $N^{G_1} = \lambda N^T$. Next we have $\boldsymbol{q}^{G_2} \sim N\left(S^G(\boldsymbol{q}^{G_1}), \frac{1}{N^{G_2}}M(S^G(\boldsymbol{q}^{G_1}))\right) = N\left(S^G(\boldsymbol{q}^{G_1}), \frac{1}{\lambda^2 N^T}M(S^G(\boldsymbol{q}^{G_1}))\right)$ where S^G is in 12-hour units and $N^{G_2} = \lambda^2 N^T$. In general we have that $\boldsymbol{q}^{G_n} \sim N\left(S^G(\boldsymbol{q}^{G_{n-1}}), \frac{1}{N^{G_n}}M(S^G(\boldsymbol{q}^{G_{n-1}}))\right) = N\left(S^G(\boldsymbol{q}^{G_{n-1}}), \frac{1}{\lambda^n N^T}M(S^G(\boldsymbol{q}^{G_{n-1}}))\right)$ with $N^{G_n} = \lambda^n N^T$ for $n > 1$. Assuming N steps in the growth process (e.g. if one step = 12 hours, then N=2 steps would correspond to a 24 hour difference between donor and recipient sampling times), then $\boldsymbol{q}^A \sim N\left(S^G(\boldsymbol{q}^{G_{N-1}}), \frac{1}{N^{G_N}}M(S^G(\boldsymbol{q}^{G_{N-1}}))\right)$. Finally, as always, $\boldsymbol{x}^A_{\mathrm{PH},i} \sim N\left(N_i^A T_i S^G(\boldsymbol{q}^A), \alpha_i N_i^A M(T_i S^G(\boldsymbol{q}^A)\right)$ where $\alpha_i = \frac{N_i^A + C}{1+C}$.

For the pre-transmission process, see main paper. For the post-transmission process, the marginalisation over $\boldsymbol{q}^B$ leads to

$$
\mathrm{E}[\boldsymbol{q}^F] = \mathrm{E}[\mathrm{E}[\boldsymbol{q}^F|\boldsymbol{q}^B]] = \mathrm{E}[\boldsymbol{q}^B] = \boldsymbol{\mu}^B \tag{33}
$$

and,

$$\begin{aligned}
\text{var}(\boldsymbol{q}^F) &= \text{E}[\text{var}[\boldsymbol{q}^F|\boldsymbol{q}^B]] + \text{var}[\text{E}[\boldsymbol{q}^F|\boldsymbol{q}^B]] \\
&= \text{E}\left[\frac{1}{N^T}M(\boldsymbol{q}^B)\right] + \text{var}\left[\boldsymbol{q}^B\right] \\
&= \frac{1}{N^T}M(\text{E}[\boldsymbol{q}^B]) + \left(1 - \frac{1}{N^T}\right)\text{var}[\boldsymbol{q}^B] \\
&= \frac{1}{N^T}M(\boldsymbol{\mu}^B) + \left(1 - \frac{1}{N^T}\right)\Sigma^B \\
&= \gamma_0 M(\boldsymbol{\mu}^B) + \delta_0\Sigma^B
\end{aligned} \tag{34}$$

where in the last step we defined $\gamma_0 = \frac{1}{N^T}$ and $\delta_0 = \left(1 - \frac{1}{N^T}\right)$ (for reasons that will become clear later).

Next, for the $\boldsymbol{q}^F$ integral, the law of total expectation yields

$$\text{E}[\boldsymbol{q}^{G_1}] = \text{E}[\text{E}[\boldsymbol{q}^{G_1}|\boldsymbol{q}^F]] = \text{E}[\boldsymbol{q}^F] = \boldsymbol{\mu}^B \tag{35}$$

Next, under the law of total variance,

$$\begin{aligned}
\text{var}(\boldsymbol{q}^{G_1}) &= \text{E}[\text{var}[\boldsymbol{q}^{G_1}|\boldsymbol{q}^F]] + \text{var}[\text{E}[\boldsymbol{q}^{G_1}|\boldsymbol{q}^F]] \\
&= \text{E}\left[\frac{1}{\lambda N^T}\left(\text{Diag}(\boldsymbol{q}^F) - \boldsymbol{q}^F(\boldsymbol{q}^F)^\dagger\right)\right] + \text{var}[\boldsymbol{q}^F] \\
&= \frac{1}{\lambda N^T}\left(\text{Diag}(\text{E}[\boldsymbol{q}^F]) - \text{E}[\boldsymbol{q}^F]E[\boldsymbol{q}^F]^\dagger\right) + \left(1 - \frac{1}{\lambda N^T}\right)\text{var}[\boldsymbol{q}^F] \\
&= \frac{1}{\lambda N^T}M\left(\boldsymbol{\mu}^B\right) + \left(1 - \frac{1}{\lambda N^T}\right)\left(\gamma_0 M(\boldsymbol{\mu}^B) + \delta_0\Sigma^B\right) \\
&= \left(\frac{1}{\lambda N^T} + \left(1 - \frac{1}{\lambda N^T}\right)\gamma_0\right)M\left(\boldsymbol{\mu}^B\right) + \left(1 - \frac{1}{\lambda N^T}\right)\delta_0\Sigma^B \\
&\equiv \gamma_{1,1}M\left(\boldsymbol{\mu}^B\right) + \delta_1\Sigma^B
\end{aligned} \tag{36}$$

where in the last step we defined $\gamma_{1,1} = \frac{1}{\lambda N^T} + \left(1 - \frac{1}{\lambda N^T}\right)\gamma_0$ and $\delta_1 = \left(1 - \frac{1}{\lambda N^T}\right)\delta_0$ (to become clear later).

Continuing with the marginalisation over $\boldsymbol{q^{G_1}}$:

$$\text{E}[\boldsymbol{q}^{G_2}] = \text{E}[\text{E}[\boldsymbol{q}^{G_2}|\boldsymbol{q}^{G_1}]] = \text{E}[S^G(\boldsymbol{q}^{G_1})] \approx S^G(\text{E}[\boldsymbol{q}^{G_1}]) = S^G(\boldsymbol{\mu}^B) \tag{37}$$

where the approximation in the penultimate step was due to the first-order second-moment method.

Next, under the law of total variance,

$$\begin{aligned}
\mathrm{var}(\boldsymbol{q}^{G_2}) &= \mathrm{E}[\mathrm{var}[\boldsymbol{q}^{G_2}|\boldsymbol{q}^{G_1}]] + \mathrm{var}[\mathrm{E}[\boldsymbol{q}^{G_2}|\boldsymbol{q}^{G_1}]] \\
&= \mathrm{E}\left[\frac{1}{\lambda^2 N^T}\left(\mathrm{Diag}(S^G(\boldsymbol{q}^{G_1})) - S^G(\boldsymbol{q}^{G_1})(S^G(\boldsymbol{q}^{G_1}))^\dagger\right)\right] + \mathrm{var}[S^G(\boldsymbol{q}^{G_1})] \\
&= \frac{1}{\lambda^2 N^T}\left(\mathrm{Diag}(\mathrm{E}[S^G(\boldsymbol{q}^{G_1})]) - \mathrm{E}[S^G(\boldsymbol{q}^{G_1})]E[S^G(\boldsymbol{q}^{G_1})]^\dagger\right) + \left(1 - \frac{1}{\lambda^2 N^T}\right)\mathrm{var}[S^G(\boldsymbol{q}^{G_1})] \\
&= \frac{1}{\lambda^2 N^T} M\left(\mathrm{E}[S^G(\boldsymbol{q}^{G_1})]\right) + \left(1 - \frac{1}{\lambda^2 N^T}\right)\mathrm{var}[S^G(\boldsymbol{q}^{G_1})] \\
&\approx \frac{1}{\lambda^2 N^T} M\left(S^G(\mathrm{E}[\boldsymbol{q}^{G_1}])\right) + \left(1 - \frac{1}{\lambda^2 N^T}\right)\left(DS^G\big|_{\mathrm{E}[\boldsymbol{q}^{G_1}]}\right)\mathrm{var}\left[\boldsymbol{q}^{G_1}\right]\left(DS^G\big|_{\mathrm{E}[\boldsymbol{q}^{G_1}]}\right)^\dagger \\
&= \frac{1}{\lambda^2 N^T} M\left(S^G(\boldsymbol{\mu}^B)\right) + \left(1 - \frac{1}{\lambda^2 N^T}\right)\left(DS^G\big|_{\boldsymbol{\mu}^B}\right)\left(\gamma_{1,1} M(\boldsymbol{\mu}^B) + \delta_1 \Sigma^B\right)\left(DS^G\big|_{\boldsymbol{\mu}^B}\right)^\dagger \\
&= \frac{1}{\lambda^2 N^T} M\left(S^G(\boldsymbol{\mu}^B)\right) + \left(1 - \frac{1}{\lambda^2 N^T}\right)\gamma_{1,1}\left(DS^G\big|_{\boldsymbol{\mu}^B}\right) M(\boldsymbol{\mu}^B)\left(DS^G\big|_{\boldsymbol{\mu}^B}\right)^\dagger + \left(1 - \frac{1}{\lambda^2 N^T}\right)\delta_1\left(DS^G\big|_{\boldsymbol{\mu}^B}\right)\Sigma^B\left(DS^G\big|_{\boldsymbol{\mu}^B}\right)^\dagger \\
&= \gamma_{2,2} M\left(S^G(\boldsymbol{\mu}^B)\right) + \gamma_{2,1}\left(DS^G\big|_{\boldsymbol{\mu}^B}\right) M(\boldsymbol{\mu}^B)\left(DS^G\big|_{\boldsymbol{\mu}^B}\right)^\dagger + \delta_2\left(DS^G\big|_{\boldsymbol{\mu}^B}\right)\Sigma^B\left(DS^G\big|_{\boldsymbol{\mu}^B}\right)^\dagger
\end{aligned} \tag{38}$$

where we defined $\gamma_{2,1} = \left(1 - \frac{1}{\lambda^2 N^T}\right)\gamma_{1,1}$, $\gamma_{2,2} = \frac{1}{\lambda^2 N^T}$ and $\delta_2 = \left(1 - \frac{1}{\lambda^2 N^T}\right)\delta_1$.

We notice that we can define the mean and variance of an arbitrary time step n as

$$\mathrm{E}[\boldsymbol{q}^{G_n}] = (S^G)^{n-1}(\boldsymbol{\mu}^B) \tag{39}$$

$$\mathrm{var}(\boldsymbol{q}^{G_n}) = \delta_n\left(DS^G\big|_{\boldsymbol{\mu}^B}\right)^{n-1}\Sigma^B\left(\left(DS^G\big|_{\boldsymbol{\mu}^B}\right)^\dagger\right)^{n-1} + \sum_{j=1}^{n}\gamma_{n,j}\left(DS^G\big|_{\boldsymbol{\mu}^B}\right)^{n-j} M\left((S^G)^{j-1}(\boldsymbol{\mu}^B)\right)\left(\left(DS^G\big|_{\boldsymbol{\mu}^B}\right)^\dagger\right)^{n-j} \tag{40}$$

where $(S^G)^k(\boldsymbol{\mu}^B)$ denotes k applications of S^G, e.g. $(S^G)^3(\boldsymbol{\mu}^B) = S^G(S^G(S^G(\boldsymbol{\mu}^B))) \neq (S^G(\boldsymbol{\mu}^B))^3$ and $\left(DS^G\big|_{\boldsymbol{\mu}^B}\right)^k$ describes the product of k Jacobian matrices: $\left(DS^G\big|_{\boldsymbol{\mu}^B}\right)^k = \prod_{j=1}^k\left(DS^G\big|_{(S^G)^{k-j}(\boldsymbol{\mu}^B)}\right)$, e.g. $\left(DS^G\big|_{\boldsymbol{\mu}^B}\right)^3 = \left(DS^G\big|_{(S^G)^2(\boldsymbol{\mu}^B)}\right)\left(DS^G\big|_{(S^G)(\boldsymbol{\mu}^B)}\right)\left(DS^G\big|_{\boldsymbol{\mu}^B}\right)$. We define $(S^G)^0(\boldsymbol{\mu}^B) = \boldsymbol{\mu}^B$ and $\left(DS^G\big|_{\boldsymbol{\mu}^B}\right)^0 = \mathbb{1}$ where $\mathbb{1}$ is the identity matrix.

The coefficients $\gamma_{n,j}$ and δ_n obey the recurrence relations:

$$\begin{aligned}
\gamma_{n,j} &= \begin{cases}\left(1 - \frac{1}{\lambda^n N^t}\right)\gamma_{n-1,j}, & \text{if } j < n \\ \frac{1}{\lambda^n N^T}, & \text{if } j = n\end{cases} \\
\delta_n &= \left(1 - \frac{1}{\lambda^n N^t}\right)\delta_{n-1}
\end{aligned} \tag{41}$$

for $n > 1$ with $\gamma_{1,1} = \frac{1}{\lambda N^T} + \left(1 - \frac{1}{\lambda N^T}\right)\frac{1}{N^T}$ and $\delta_1 = \left(1 - \frac{1}{\lambda N^T}\right)\left(1 - \frac{1}{N^T}\right)$.

5 Selection for Transmissibility and Within-Host Adaptation

Here we consider selection both for increased transmissibility and within-host adaptation. As previously we have $\boldsymbol{q}^F \sim N\left(S^T(\boldsymbol{q}^B), \frac{1}{N^T}M(S^T(\boldsymbol{q}^B))\right)$ where $\beta_i = \frac{N_i^B + C}{1 + C}$ and $\boldsymbol{q}^{G_1} \sim N\left(\boldsymbol{q}^F, \frac{1}{N^{G_1}}M(\boldsymbol{q}^F)\right) = N\left(\boldsymbol{q}^F, \frac{1}{\lambda N^T}M(\boldsymbol{q}^F)\right)$ with $N^{G_1} = \lambda N^T$. Next we have $\boldsymbol{q}^{G_2} \sim N\left(S^G(\boldsymbol{q}^{G_1}), \frac{1}{N^{G_2}}M(S^G(\boldsymbol{q}^{G_1}))\right) =$

$N\left(S^G(\boldsymbol{q}^{G_1}), \frac{1}{\lambda^2 N^T} M(S^G(\boldsymbol{q}^{G_1}))\right)$ where S^G is in 12-hour units and $N^{G_2} = \lambda^2 N^T$. In general we have that $\boldsymbol{q}^{G_n} \sim N\left(S^G(\boldsymbol{q}^{G_{n-1}}), \frac{1}{N^{G_n}} M(S^G(\boldsymbol{q}^{G_{n-1}}))\right) = N\left(S^G(\boldsymbol{q}^{G_{n-1}}), \frac{1}{\lambda^n N^T} M(S^G(\boldsymbol{q}^{G_{n-1}}))\right)$ with $N^{G_n} = \lambda^n N^T$ for $n > 1$. Assuming N steps in the growth process (e.g. if one step = 12 hours, then N=2 steps would correspond to a 24 hour difference between donor and recipient sampling times), then $\boldsymbol{q}^A \sim N\left(S^G(\boldsymbol{q}^{G_{N-1}}), \frac{1}{N^{G_N}} M(S^G(\boldsymbol{q}^{G_{N-1}}))\right)$. Finally, as always, $\boldsymbol{x}^A_{\mathrm{PH},i} \sim N\left(N_i^A T_i S^G(\boldsymbol{q}^A), \alpha_i N_i^A M(T_i S^G(\boldsymbol{q}^A)\right)$ where $\alpha_i = \frac{N_i^A + C}{1+C}$.

For the pre-transmission process, see main paper. For the post-transmission process, the marginalisation over $\boldsymbol{q}^B$ leads to

$$\mathrm{E}[\boldsymbol{q}^F] = \mathrm{E}[\mathrm{E}[\boldsymbol{q}^F|\boldsymbol{q}^B]] = \mathrm{E}[S^T(\boldsymbol{q}^B)] \approx S^T(\mathrm{E}[\boldsymbol{q}^B]) = S^T(\boldsymbol{\mu}^B) \tag{42}$$

where in the penultimate step we used the first-order second-moment approximation to a vector function acting on a random variable. The law of total variance yields

$$\begin{aligned}
\mathrm{var}(\boldsymbol{q}^F) &= \mathrm{E}[\mathrm{var}[\boldsymbol{q}^F|\boldsymbol{q}^B]] + \mathrm{var}[\mathrm{E}[\boldsymbol{q}^F|\boldsymbol{q}^B]] \\
&= \mathrm{E}\left[\frac{1}{N^T} M(S^T(\boldsymbol{q}^B))\right] + \mathrm{var}\left[S^T(\boldsymbol{q}^B)\right] \\
&= \frac{1}{N^T} M(\mathrm{E}[S^T(\boldsymbol{q}^B)]) + \left(1 - \frac{1}{N^T}\right) \mathrm{var}[S^T(\boldsymbol{q}^B)] \\
&\approx \frac{1}{N^T} M(S^T(\mathrm{E}[\boldsymbol{q}^B])) + \left(1 - \frac{1}{N^T}\right) \left(DS^T\big|_{\mathrm{E}[\boldsymbol{q}^B]}\right) \mathrm{var}[\boldsymbol{q}^B] \left(DS^T\big|_{\mathrm{E}[\boldsymbol{q}^B]}\right)^\dagger \\
&= \frac{1}{N^T} M(S^T(\boldsymbol{\mu}^B)) + \left(1 - \frac{1}{N^T}\right) \left(DS^T\big|_{\boldsymbol{\mu}^B}\right) \Sigma^B \left(DS^T\big|_{\boldsymbol{\mu}^B}\right)^\dagger \\
&= \gamma_0 M(S^T(\boldsymbol{\mu}^B)) + \delta_0 \left(DS^T\big|_{\boldsymbol{\mu}^B}\right) \Sigma^B \left(DS^T\big|_{\boldsymbol{\mu}^B}\right)^\dagger
\end{aligned} \tag{43}$$

where in the last step we defined $\gamma_0 = \frac{1}{N^T}$ and $\delta_0 = \left(1 - \frac{1}{N^T}\right)$. We also note that $(DS)_i^j = \frac{\partial S_i}{\partial q_j}$ is the Jacobian matrix arising from the first-order second-moment approximation.

Next, for the $\boldsymbol{q}^F$ integral, the law of total expectation yields

$$\mathrm{E}[\boldsymbol{q}^{G_1}] = \mathrm{E}[\mathrm{E}[\boldsymbol{q}^{G_1}|\boldsymbol{q}^F]] = \mathrm{E}[\boldsymbol{q}^F] = S^T(\boldsymbol{\mu}^B) \tag{44}$$

Next, under the law of total variance,

$$\begin{aligned}
\mathrm{var}(\boldsymbol{q}^{G_1}) &= \mathrm{E}[\mathrm{var}[\boldsymbol{q}^{G_1}|\boldsymbol{q}^F]] + \mathrm{var}[\mathrm{E}[\boldsymbol{q}^{G_1}|\boldsymbol{q}^F]] \\
&= \mathrm{E}\left[\frac{1}{\lambda N^T}\left(\mathrm{Diag}(\boldsymbol{q}^F) - \boldsymbol{q}^F(\boldsymbol{q}^F)^\dagger\right)\right] + \mathrm{var}[\boldsymbol{q}^F] \\
&= \frac{1}{\lambda N^T}\left(\mathrm{Diag}(\mathrm{E}[\boldsymbol{q}^F]) - \mathrm{E}[\boldsymbol{q}^F] E[\boldsymbol{q}^F]^\dagger\right) + \left(1 - \frac{1}{\lambda N^T}\right) \mathrm{var}[\boldsymbol{q}^F] \\
&= \frac{1}{\lambda N^T} M\left(S^T(\boldsymbol{\mu}^B)\right) + \left(1 - \frac{1}{\lambda N^T}\right)\left(\gamma_0 M(S^T(\boldsymbol{\mu}^B)) + \delta_0 \left(DS^T\big|_{\boldsymbol{\mu}^B}\right) \Sigma^B \left(DS^T\big|_{\boldsymbol{\mu}^B}\right)^\dagger\right) \\
&= \left(\frac{1}{\lambda N^T} + \left(1 - \frac{1}{\lambda N^T}\right)\gamma_0\right) M\left(S^T(\boldsymbol{\mu}^B)\right) + \left(1 - \frac{1}{\lambda N^T}\right)\delta_0 \left(DS^T\big|_{\boldsymbol{\mu}^B}\right) \Sigma^B \left(DS^T\big|_{\boldsymbol{\mu}^B}\right)^\dagger \\
&\equiv \gamma_{1,1} M\left(S^T(\boldsymbol{\mu}^B)\right) + \delta_1 \left(DS^T\big|_{\boldsymbol{\mu}^B}\right) \Sigma^B \left(DS^T\big|_{\boldsymbol{\mu}^B}\right)^\dagger
\end{aligned} \tag{45}$$

where in the last step we defined $\gamma_{1,1} = \frac{1}{\lambda N^T} + \left(1 - \frac{1}{\lambda N^T}\right)\gamma_0$ and $\delta_1 = \left(1 - \frac{1}{\lambda N^T}\right)\delta_0$ (to become clear later).

Continuing with the marginalisation over $\boldsymbol{q}^{\boldsymbol{G_1}}$:

$$\mathrm{E}[\boldsymbol{q}^{G_2}] = \mathrm{E}[\mathrm{E}[\boldsymbol{q}^{G_2}|\boldsymbol{q}^{G_1}]] = \mathrm{E}[S^G(\boldsymbol{q}^{G_1})] \approx S^G(\mathrm{E}[\boldsymbol{q}^{G_1}]) = S^G(S^T(\boldsymbol{\mu}^B)) \tag{46}$$

where the approximation in the penultimate step was due to the first-order second-moment method.

Next, under the law of total variance,

$$\begin{aligned}
\mathrm{var}(\boldsymbol{q}^{G_2}) &= \mathrm{E}[\mathrm{var}[\boldsymbol{q}^{G_2}|\boldsymbol{q}^{G_1}]] + \mathrm{var}[\mathrm{E}[\boldsymbol{q}^{G_2}|\boldsymbol{q}^{G_1}]] \\
&= \mathrm{E}\left[\frac{1}{\lambda^2 N^T}\left(\mathrm{Diag}(S^G(\boldsymbol{q}^{G_1})) - S^G(\boldsymbol{q}^{G_1})(S^G(\boldsymbol{q}^{G_1}))^\dagger\right)\right] + \mathrm{var}[S^G(\boldsymbol{q}^{G_1})] \\
&= \frac{1}{\lambda^2 N^T}\left(\mathrm{Diag}(\mathrm{E}[S^G(\boldsymbol{q}^{G_1})]) - \mathrm{E}[S^G(\boldsymbol{q}^{G_1})]E[S^G(\boldsymbol{q}^{G_1})]^\dagger\right) + \left(1 - \frac{1}{\lambda^2 N^T}\right)\mathrm{var}[S^G(\boldsymbol{q}^{G_1})] \\
&= \frac{1}{\lambda^2 N^T} M\left(\mathrm{E}[S^G(\boldsymbol{q}^{G_1})]\right) + \left(1 - \frac{1}{\lambda^2 N^T}\right)\mathrm{var}[S^G(\boldsymbol{q}^{G_1})] \\
&\approx \frac{1}{\lambda^2 N^T} M\left(S^G(\mathrm{E}[\boldsymbol{q}^{G_1}])\right) + \left(1 - \frac{1}{\lambda^2 N^T}\right)\left(DS^G\big|_{\mathrm{E}[\boldsymbol{q}^{G_1}]}\right)\mathrm{var}\left[\boldsymbol{q}^{G_1}\right]\left(DS^G\big|_{\mathrm{E}[\boldsymbol{q}^{G_1}]}\right)^\dagger \\
&= \frac{1}{\lambda^2 N^T} M\left(S^G(S^T(\boldsymbol{\mu}^B))\right) + \left(1 - \frac{1}{\lambda^2 N^T}\right)\left(DS^G\big|_{S^T(\boldsymbol{\mu}^B)}\right)\left(\gamma_{1,1} M\left(S^T(\boldsymbol{\mu}^B)\right) + \delta_1\left(DS^T\big|_{\boldsymbol{\mu}^B}\right)\Sigma^B\left(DS^T\big|_{\boldsymbol{\mu}^B}\right)^\dagger\right)\left(DS^G\big|_{S^T(\boldsymbol{\mu}^B)}\right)^\dagger \\
&= \frac{1}{\lambda^2 N^T} M\left(S^G(S^T(\boldsymbol{\mu}^B))\right) + \left(1 - \frac{1}{\lambda^2 N^T}\right)\gamma_{1,1}\left(DS^G\big|_{S^T(\boldsymbol{\mu}^B)}\right) M(S^T(\boldsymbol{\mu}^B)\left(DS^G\big|_{S^T(\boldsymbol{\mu}^B)}\right)^\dagger + \left(1 - \frac{1}{\lambda^2 N^T}\right)\delta_1\left(DS^G\big|_{S^T(\boldsymbol{\mu}^B)}\right)\left(DS^T\big|_{\boldsymbol{\mu}^B}\right)\Sigma^B\left(DS^T\big|_{\boldsymbol{\mu}^B}\right)^\dagger\left(DS^G\big|_{S^T(\boldsymbol{\mu}^B)}\right)^\dagger \\
&= \gamma_{2,2} M\left(S^G(S^T(\boldsymbol{\mu}^B))\right) + \gamma_{2,1}\left(DS^G\big|_{S^T(\boldsymbol{\mu}^B)}\right) M(S^T(\boldsymbol{\mu}^B))\left(DS^G\big|_{S^T(\boldsymbol{\mu}^B)}\right)^\dagger + \delta_2\left(DS^G\big|_{S^T(\boldsymbol{\mu}^B)}\right)\left(DS^T\big|_{\boldsymbol{\mu}^B}\right)\Sigma^B\left(DS^T\big|_{\boldsymbol{\mu}^B}\right)^\dagger\left(DS^G\big|_{S^T(\boldsymbol{\mu}^B)}\right)^\dagger
\end{aligned} \tag{47}$$

where we defined $\gamma_{2,1} = \left(1 - \frac{1}{\lambda^2 N^T}\right)\gamma_{1,1}$, $\gamma_{2,2} = \frac{1}{\lambda^2 N^T}$ and $\delta_2 = \left(1 - \frac{1}{\lambda^2 N^T}\right)\delta_1$.

We notice that we can define the mean and variance of an arbitrary time step n as

$$\mathrm{E}[\boldsymbol{q}^{G_n}] = (S^G)^{n-1}(S^T(\boldsymbol{\mu}^B)) \tag{48}$$

$$\mathrm{var}(\boldsymbol{q}^{G_n}) = \delta_n\left(DS^G\big|_{S^T(\boldsymbol{\mu}^B)}\right)^{n-1}\left(DS^T\big|_{\boldsymbol{\mu}^B}\right)\Sigma^B\left(DS^T\big|_{\boldsymbol{\mu}^B}\right)^\dagger\left(\left(DS^G\big|_{S^T(\boldsymbol{\mu}^B)}\right)^\dagger\right)^{n-1} + \sum_{j=1}^{n}\gamma_{n,j}\left(DS^G\big|_{S^T(\boldsymbol{\mu}^B)}\right)^{n-j} M\left((S^G)^{j-1}(S^T(\boldsymbol{\mu}^B))\right)\left(\left(DS^G\big|_{S^T(\boldsymbol{\mu}^B)}\right)^\dagger\right)^{n-j} \tag{49}$$

where $(S^G)^k(S^T(\boldsymbol{\mu}^B))$ denotes k applications of S^G, e.g. $(S^G)^3(S^T(\boldsymbol{\mu}^B)) = S^G(S^G(S^G(S^T(\boldsymbol{\mu}^B)))) \neq (S^G(S^T(\boldsymbol{\mu}^B))^3$ and $\left(DS^G\big|_{S^T(\boldsymbol{\mu}^B)}\right)^k$ describes the product of k Jacobian matrices: $\left(DS^G\big|_{S^T(\boldsymbol{\mu}^B)}\right)^k = \prod_{j=1}^k\left(DS^G\big|_{(S^G)^{k-j}(S^T(\boldsymbol{\mu}^B))}\right)$ e.g. $\left(DS^G\big|_{S^T(\boldsymbol{\mu}^B)}\right)^3 = \left(DS^G\big|_{(S^G)^2(S^T(\boldsymbol{\mu}^B))}\right)\left(DS^G\big|_{(S^G)(S^T(\boldsymbol{\mu}^B))}\right)\left(DS^G\big|_{S^T(\boldsymbol{\mu}^B)}\right)$. We define $(S^G)^0(S^T(\boldsymbol{\mu}^B)) = S^T(\boldsymbol{\mu}^B)$ and $\left(DS^G\big|_{S^T(\boldsymbol{\mu}^B)}\right)^0 = \mathbb{1}$ where $\mathbb{1}$ is the identity matrix.

The coefficients $\gamma_{n,j}$ and δ_n obey the recurrence relations:

$$\gamma_{n,j} = \begin{cases}\left(1 - \frac{1}{\lambda^n N^t}\right)\gamma_{n-1,j}, & \text{if } j < n \\ \frac{1}{\lambda^n N^T}, & \text{if } j = n\end{cases}$$

$$\delta_n = \left(1 - \frac{1}{\lambda^n N^t}\right)\delta_{n-1} \tag{50}$$

for $n > 1$ with $\gamma_{1,1} = \frac{1}{\lambda N^T} + \left(1 - \frac{1}{\lambda N^T}\right)\frac{1}{N^T}$ and $\delta_1 = \left(1 - \frac{1}{\lambda N^T}\right)\left(1 - \frac{1}{N^T}\right)$.
