# Supplementary material for: A novel framework for inferring parameters of transmission from viral sequence data
Source: PLoS Genet. 2018 Oct 16;14(10):e1007718. doi: 10.1371/journal.pgen.1007718 (PMC6203404; doi:10.1371/journal.pgen.1007718)
Supplement: S2 Text — (PDF) [file pgen.1007718.s017.pdf]

Supplementary Text 2

Proof that $T\text{Diag}(\boldsymbol{q})T^{\dagger} = \text{Diag}(T\boldsymbol{q})$

1 Introduction

In our derivations of compound distributions we use the identity that $T\text{Diag}(\boldsymbol{q})T^{\dagger} = \text{Diag}(T\boldsymbol{q})$ is true for a $J \times K$ matrix T and a K dimensional vector $\boldsymbol{q}$ if T consists of zeroes and ones and if every column of T contains a single non-zero element, i.e. if a full haplotype can only contribute to a single partial haplotype in the partial haplotype set. Here we have suppressed the subscripts denoting partial haplotype sets to avoid confusion in the subsequent derivation.

2 Proof

Considering first the right hand side of the identity, we see that

$$\text{Diag}(T\boldsymbol{q})_{i,j} = \begin{cases} T_{i,k}q_k, & \text{if } i = j \\ 0, & \text{if } i \neq j \end{cases}$$

where we have used implicit summation over the k index.

Considering the left hand side of the identity, we may examine the cases of $i = j$ and $i \neq j$ separately. For $i = j$ we can write the left hand side as

$$\left(T\text{Diag}(\boldsymbol{q})T^{\dagger}\right)_{i,i} = T_{i,k}\text{Diag}(\boldsymbol{q})_{k,l}T^{\dagger}_{l,i} = T_{i,k}\delta_{k,l}q_l T^{\dagger}_{l,i} \tag{1}$$

where there is no summation over the i indices. Additionally we have represented $\text{Diag}(\boldsymbol{q})_{k,l}$ as $\delta_{k,l}q_l$ where $\delta_{k,l}$ is the Kronecker delta/identity matrix. Here there is no summation over l, even though, slightly confusingly, the summation over l is implied in $\text{Diag}(\boldsymbol{q})_{k,l}T^{\dagger}_{l,i}$. In other words, q_l may be considered an index-valued scaling factor to the summed over matrix $\delta_{k,l}$.

Using $T^{\dagger}_{l,i} = T_{i,l}$ and the replacement properties of the Kronecker delta yields

$$\left(T\text{Diag}(\boldsymbol{q})T^{\dagger}\right)_{i,i} = T_{i,k}\delta_{k,l}q_l T_{i,l} = T_{i,k}q_k T_{i,k} = q_k \left(T_{i,k}\right)^2 \tag{2}$$

As all entries of T are zeroes and ones, we must have that $\left(T_{i,k}\right)^2 = T_{i,k}$. Thus,

$$\left(T\text{Diag}(\boldsymbol{q})T^{\dagger}\right)_{i,i} = T_{i,k}q_k \tag{3}$$

as required.

Considering the case of $i \neq j$:

$$\left(T\text{Diag}(\boldsymbol{q})T^\dagger\right)_{i,j} = T_{i,k}\text{Diag}(\boldsymbol{q})_{k,l}T^\dagger_{l,j} = T_{i,k}\delta_{k,l}q_l T^\dagger_{l,j} = T_{i,k}\delta_{k,l}q_l T_{j,l} = T_{i,k}q_k T_{j,k} \tag{4}$$

Now we use the property that each column of T must have exactly one non-zero entry. This implies that $T_{i,k}T_{j,k} = 0$ if $i \neq j$. Thus, for $i \neq j$,

$$\left(T\text{Diag}(\boldsymbol{q})T^\dagger\right)_{i,j} = 0 \tag{5}$$

as required. This proves the identity.
