# Supplementary material for: A novel framework for inferring parameters of transmission from viral sequence data
Source: PLoS Genet. 2018 Oct 16;14(10):e1007718. doi: 10.1371/journal.pgen.1007718 (PMC6203404; doi:10.1371/journal.pgen.1007718)
Supplement: S1 Table — Parameters were inferred across all index and contact ferrets within each experiment and are reported to a single decimal place. Only polymorphisms at which within-host selection was identified are listed. The parameter χ denotes an epistatic interaction between variant alleles. We note that our method infers the approximate shape of a fitness landscape based upon a reconstruction of whole viral segments; individual selection coefficients may be subject to variance between similar fitness landscapes. (PDF) [file pgen.1007718.s018.pdf]

Segment	Variant	Mut	HA190D220D
PB2	A1199G	-0.9	
PB2	T1537C		0.5
PB2	G2193C		-0.3
PB1	C65T		-0.4
PB1	C90A	-0.3	-0.3
PB1	C835A		0.3
PB1	C982T		0.4
PB1	T1151G		0.3
PB1	G2250T	-1	
PB1	$\chi_{90,982}$		1
PA	A781G	-0.2	
PA	G1500T		0.5
PA	C1651T	-0.7	
PA	G1880T	-0.8	
HA	G14T	-0.0	0.4
HA	A400G	0.2	-0.3
HA	A507C	0.5	0.4
HA	C550A	0.3	
HA	T634C	0.4	
HA	A649G		0.3
HA	A651C	0.6	
HA	T653G	0.5	
HA	G741A		-0.1
HA	G747A	0.2	
HA	A748G	0.3	
HA	A868T	0.2	0.3
HA	T1036C		-2.1
HA	C1263A		0.5
HA	C1762T	-0.7	
HA	$\chi_{14,400}$	-0.3	
HA	$\chi_{14,507}$	0.2	
HA	$\chi_{400,507}$	-36.5	
HA	$\chi_{400,550}$	-32.2	
HA	$\chi_{400,1036}$		2.2
HA	$\chi_{868,1263}$		-0.8
NP	G600A		0.4
NA	G440A		0.4
NA	G649A		0.2
NS	G289A		0.4
